# Cycloaddition Chemistry of a Silylene‐Nickel Complex toward Organic π‐Systems: From Reversibility to C−H Activation

**DOI:** 10.1002/chem.202000009

**Published:** 2020-01-31

**Authors:** Terrance J. Hadlington, Arseni Kostenko, Matthias Driess

**Affiliations:** ^1^ Department of Chemistry, Metalorganics and Inorganic Materials Technische Universität Berlin Strasse des 17. Juni 135, Sekr. C2 10623 Berlin Germany

**Keywords:** carbene analogues, bond activation, metal complexes, silametallacycles, silicon

## Abstract

The versatile cycloaddition chemistry of the Si−Ni multiple bond in the acyclic (amido)(chloro)silylene→Ni^0^ complex **1**, [(^TMS^L)ClSi→Ni(NHC)_2_] (^TMS^L=N(SiMe_3_)Dipp; Dipp=2,6‐*i*Pr_2_C_6_H_4_; NHC=C[(*i*Pr)NC(Me)]_2_), toward unsaturated organic substrates is reported, which is both reminiscent of and expanding on the reactivity patterns of classical Fischer and Schrock carbene–metal complexes. Thus, 1:1 reaction of **1** with aldehydes, imines, alkynes, and even alkenes proceed to yield [2+2] cycloaddition products, leading to a range of four‐membered metallasilacycles. This cycloaddition is in fact reversible for ethylene, whereas addition of an excess of this olefin leads to quantitative sp^2^‐CH bond activation, via a 1‐nickela‐4‐silacyclohexane intermediate. These results have been supported by DFT calculations giving insights into key mechanistic aspects.

## Graphical abstract

**2+2=score**! The diverse [2+2] cycloaddition of organic π‐systems with a silicon–metal double bond is demonstrated, leading to a range of four‐membered metallasilacycles. Notably, this process is reversible for ethylene, with the mechanism for both alkene and alkyne addition found to mimic that for the reaction of olefins with classical carbene–metal complexes.

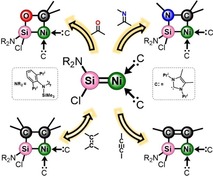

The importance of cycloaddition reactions of carbon–metal π‐bonds in catalysis cannot be overstated, paramount in processes such as alkene metathesis and cyclopropanation.^1^ In these systems, highly reactive carbon–metal multiple bonds can undergo formal [2+2] cycloaddition reactions with carbon–carbon or carbon–heteroatom multiple bonds, typically through a [2+1] addition of the unsaturated species at the metal center, with subsequent chemistry leading to cyclopropyl or metathesized products.1d, 2 Thus, such systems have been studied extensively since the seminal discovery of a stable carbene–metal complex by Fischer et al.1a, 3 Notably, N‐heterocyclic carbenes (NHCs), as well as other stable carbene systems, are broadly utilized as ligands in transition‐metal chemistry, but their carbene–metal bonds are typically unreactive toward C−X π‐bonds (X=C, heteroatoms).^4^ More recently, N‐heterocyclic silylene (NHSi)–transition‐metal complexes, which contain a dative Si^II^→M σ‐bond, have seen considerable attention,^5^ with examples in which the divalent silicon center is in fact directly involved in bond activation processes.^6^ Examples of Si:→M multiple bonds have also seen considerable precedent in the literature.^7^, ^8^ Nevertheless, examples of cycloaddition chemistry of these moieties are somewhat sparse. Addition of alkynes and phosphalkynes to threefold‐bonded Si−Os,^9^ and ketones and carbodiimides to threefold‐bonded Si−W species have been reported,^10^ somewhat comparable with the wide‐ranging and versatile cycloaddition chemistry of homonuclear E−E multiple bonds (E=Si, Ge, Sn).^11^, ^12^ Well‐defined examples of the cycloaddition chemistry of Si−M double bonds are limited to reports from Sekiguchi et al. (Figure 1), in the [2+2] addition of alkynes and benzonitrile to a Si=Ti bond.^13^ These remarkable reports are reminiscent of key steps in the metathesis reactions of classical Schrock‐type carbene complexes.^2^ The exciting synthetic utility of Si−M multiple bonds in this regard thus warrants considerable further investigation, and could pave the way to new functional silicon‐containing organic molecules which are otherwise difficult to prepare. Indeed, metal–silylene complexes have been highlighted as potential key intermediates in important catalytic processes such as hydrosilylation,7c, 14 whereas unsaturated four‐membered sila‐metallacycles have also been inferred as intermediates in the catalytic ring‐expansion of silacyclopropanes.^15^ We wished to gain further insights into the chemistry of such metallacycles by employing the previously reported acyclic silylene–Ni^0^ complex, ^TMS^L(Cl)Si:→Ni(NHC)_2_ **1** (^TMS^L=[(Dipp)(SiMe_3_)N]^−^; Dipp=C_6_H_3_‐*i*Pr‐2,6; NHC=[:C{N(*i*Pr)C(Me)}_2_]), which possesses a degree of Si−Ni multiple‐bond character.^16^ We envisaged that cycloaddition chemistry with unsaturated organic compounds may be possible utilizing **1**. Herein, we demonstrate that the Si−Ni multiple bond in **1** readily undergoes [2+2] cycloaddition reactions with a range of unsaturated C−X bonds (X=C, N, O) The further chemistry of isolated four‐membered nickelasilacycles reveals both reversibility in this cycloaddition process for ethylene, as well as the facile and stoichiometric activation of inert C−H bonds. The computationally derived mechanism for the latter process with ethylene operates via a reactive 1‐nickela‐4‐silacyclohexane intermediate, formed through a formal [2+2+2] cycloaddition reaction of two ethylene molecules with **1**.

**Figure 1:**
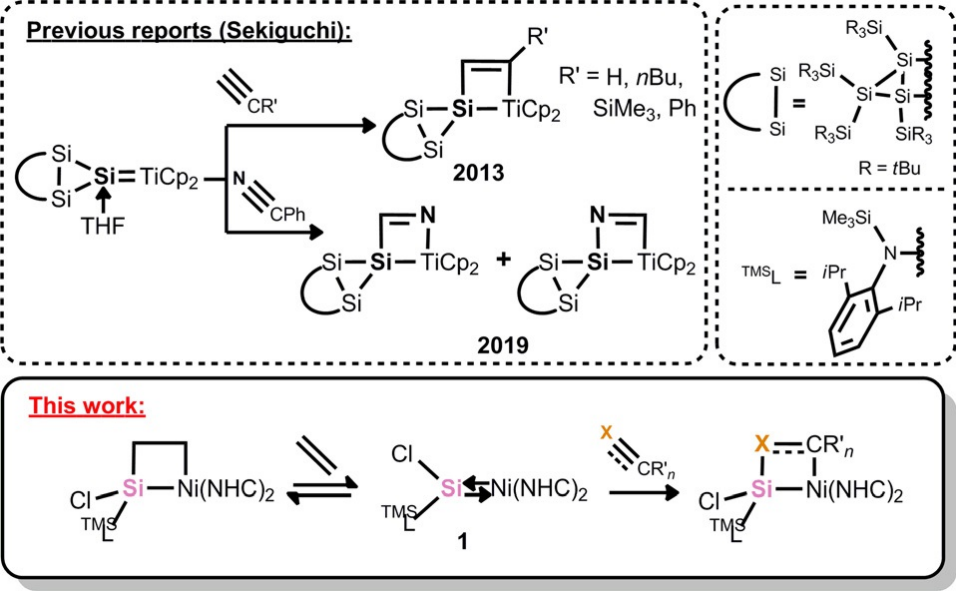
Top: Reported cycloaddition reactions of a Ti−Si double bond. Bottom: General scheme for the cycloaddition reactivity of the Si−Ni bond in **1**. R’=organic group; ^TMS^L=[(Dipp)(SiMe_3_)N]; Dipp=C_6_H_3_‐*i*Pr‐2,6; NHC=[:C{N(*i*Pr)C(Me)}_2_]; X = O, NR′, or CR′_2_.

As mentioned, **1** shows some degree of backbonding from nickel to silicon, resulting in a Si−Ni interaction with some multiple bond character (WBI=MBO=1.29; WBI: Wiberg bond indices; MBO: Mayer bond order).^16^ This, alongside the relative polarity in this bond, led us to hypothesize that **1** should be reactive towards unsaturated C−X bonds (X=C, N, O), given the prominence of such chemistry in reactive carbene–transition‐metal complexes. Initial efforts towards this end focused on phenyl acetylene, and related reports for Ti=Si bonds from Sekiguchi et al.^13^ Deeply red‐purple‐colored solutions are immediately obtained upon addition of one molar equiv of phenyl acetylene to **1**, with quantitative formation of a single product suggested by ^1^H NMR analysis of the reaction mixtures. However, X‐ray analysis of suitable single crystals obtained from reaction mixtures indicated that C−H activation of the acidic acetylene proton had in fact occurred at Si^II^, yielding a Ni^0^ π‐complex of a (phenyl)(silyl)acetylene derivative (**2**, Scheme 1).^17^ Similarly, the C−H activation product **3** was also obtained in the reaction of **1** with acetophenone, due to enolization of this ketone (Figure S40, Supporting Information), indicating that the tolerance of **1** towards relatively acidic C−H moieties is low.

**Scheme 1:**
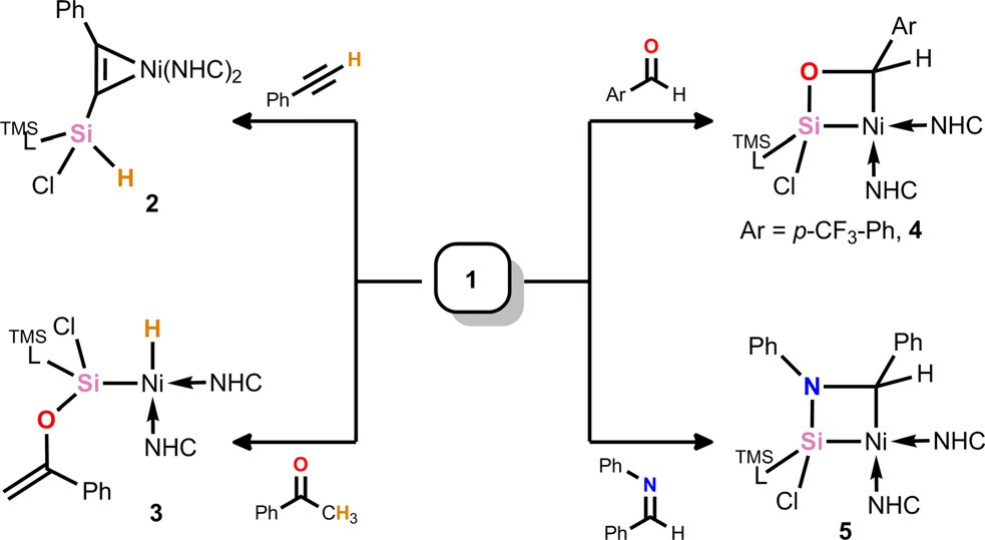
C−H activation chemistry in the reactions of **1** with acetophenone and phenyl acetylene versus cycloaddition reactions with benzaldehyde and *N*‐benzylideneaniline derivatives.

To circumvent formation of an enolized product, **1** was reacted with *p*‐CF_3_‐benzaldehyde, as well as the related imine, *N*‐benzylideneaniline. We found that in both cases the desired [2+2] cycloaddition products **4** and **5** were quantitatively formed, respectively. Both compounds show highly unsymmetrical environments for their NHC and ^TMS^L ligands in their ^1^H NMR spectra, due to the rigid four‐membered ring at their core. The molecular structure of each species shows activation of their formerly C−O/N multiple bonds, and Ni−Si bond lengths in keeping with single bonds (Figures S40 and S41, Supporting Information), considerably lengthened relative to that in **1**. Although meaningful ^29^Si NMR data could not be obtained for **4**, due to solubility issues in solvents with which **4** does not react, the ^29^Si NMR spectrum of **5** shows a markedly highfield shift for its formerly Si^II^ center (**1**: *δ*=123.2; **4**: *δ*=−65.4 ppm).

Given that relative atomic charges derived from an NPA (natural population analysis) of **1** indicates a positive relative charge at silicon (NPA_Si_=+1.14; NPA_Ni_=−0.59), it's not surprising that in the aforementioned cases the heteroatom binds silicon, forming planar and cyclic [SiNiCX] cores (X=O or N). This is in contrast to the reactivity of Sekiguchi's titanium–silylene complex (Figure 1), which forms both regio‐isomers in the reaction with benzonitrile.13c Observing the frontier orbitals of **1**, one can see that the LUMO represents the π*‐orbital of the Si−Ni bond, considerably weighted towards Si^II^, whereas the HOMO is a filled 3 d orbital at Ni^0^. Thus, a mechanism of initial oxygen/nitrogen donation to silicon, followed by Ni→C nucleophilic attack can be proposed. This was corroborated by a DFT analysis, in which the most favorable reaction coordinate involves a concerted [2+2] cycloaddition, directly leading to **4** and **5** in a single step (Figure S45, Supporting Information). Interestingly, observing the HOMO−1 and the LUMO+1 of **1** (−3.05 and −0.38 eV, respectively), which are close in energy to the HOMO and LUMO (−2.84 and −0.45 eV, respectively),^16^ it is clear that these orbitals may too be involved in the reactivity of **1**, both being of π‐symmetry; these are notably similar to those orbitals in a previously reported silylene–Pt complex.^18^

Compound **1** was treated with acetylene and ethylene to generate nickelasila‐cyclobutene and ‐cyclobutane derivatives. Indeed, the former is particularly interesting given previous investigations into the metallacyclobutene–vinyl carbene equilibrium for the ‘all‐carbon’ system.^19^ Addition of approx. one molar equiv of either ethylene or acetylene to solutions of **1** in diethyl ether or toluene, respectively, at −78 °C led to an immediate color change to bright yellow. ^1^H NMR experiments carried out in parallel indicated that a single highly unsymmetrical species is formed in both cases. Similar to compounds **4** and **5**, we proposed that the source of this asymmetry was the formation of metallacycles, locking the formed species in a single conformer with an asymmetrical Si‐center. Structural analysis of the products from these reaction mixtures confirmed that cycloaddition of acetylene and ethylene had occurred, forming nickelasila‐cyclobutene and ‐cyclobutane derivatives **6** and **7**, respectively (Scheme 2, Figure 2). Although no such species have been crystallographically characterized for Ni,^20^ metallosila‐cyclobutenes are known for Ti and Pd,^13^, ^21^ the Ti derivatives being generated through [2+2] cycloaddition (see above). The four‐membered core of both **6** and **7** is planar, as in **4** and **5**. The C−C distance in the core of **6** (1.344(3) Å), however, is considerably contracted relative to that in **7** (1.556(3) Å), indicative of double‐bond character. Further analysis of bond lengths in nickelasila‐cyclobutene **6** indicates typical C−Si, Si−Ni, and C−Ni single bonds, thus indicating that there is no degree of formation of the vinyl carbene complex **6’**, which was found to be 32.8 kcal mol^−1^ higher in energy than cyclic **6** (Scheme 2). The Si−C and C−C bond lengths in the cyclic core of **6** also compare well with the acetylene derived titanasila‐cyclobutene reported by Sekiguchi,^[13(b)]^ pertaining to a formal metallacyclic structure, whereas related distances in both the SiMe_3_ and *n*Bu substituted derivatives reported by the same group pertain to a degree of metal–silylidene alkyne π‐complex character (that is a contracted Ti−Si distance and an elongated Si−C^alkyne^ distance).13a This observation is most likely caused by the increased steric profile of the alkyne substrates in the latter. In an attempt to observe a similar trend for our system, **1** was reacted with 1,4‐dimethoxy‐2‐butyne, generating a much more sterically crowded derivate of **6**, namely **6‐OMe** (Scheme 2). However, we found that **6‐OMe** is essentially isostructural to **6** (see Figure S43 in the Supporting Information). Bulkier alkynes did not show any reaction with **1**, even after heating.

**Scheme 2:**
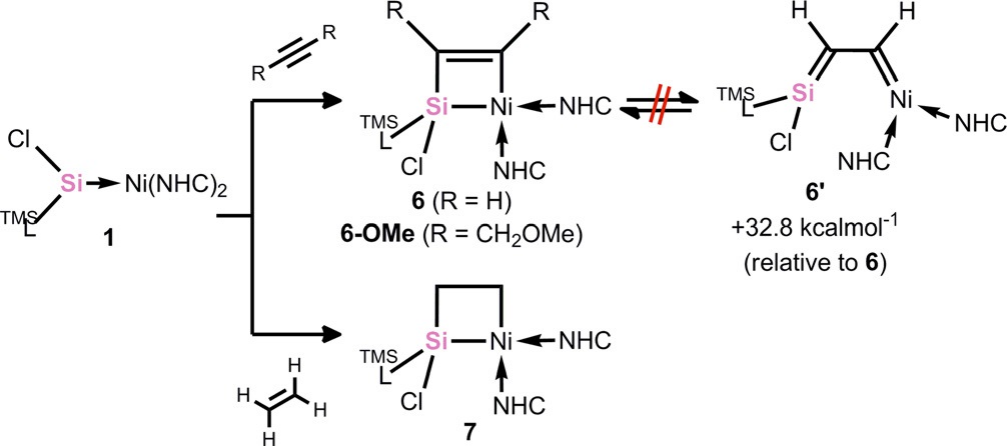
The [2+2] cycloaddition reactions of ethylene and acetylene derivatives with **1**, and the calculated energy for the isomerization of metallacyclobutene derivative **6** to silene–carbene complex **6’**.

**Figure 2:**
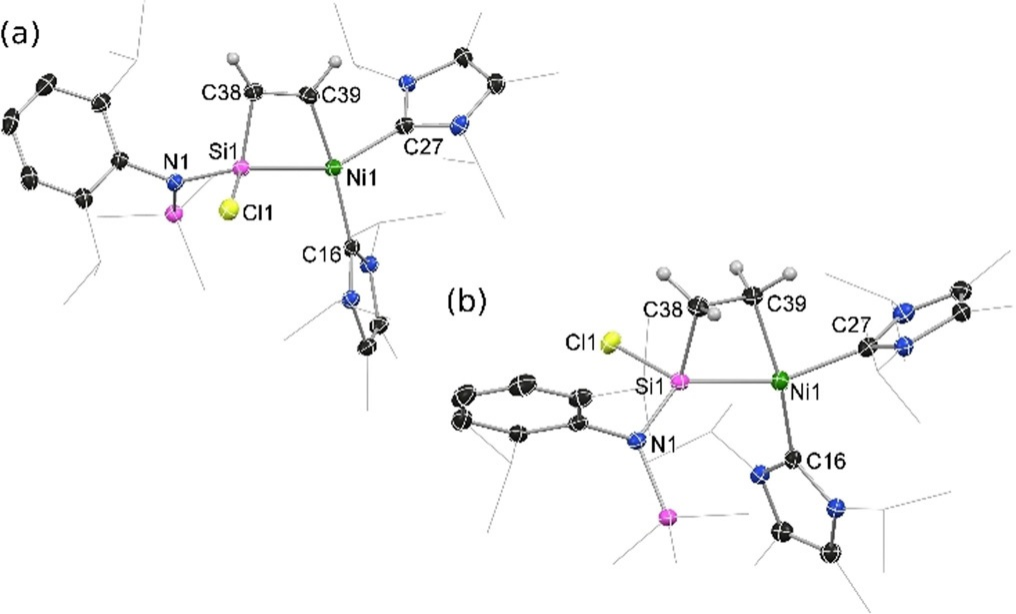
The molecular structures of (a) **6**, and (b) **7**, with thermal ellipsoids at 30 % probability. Selected bond lengths (Å) and angles (°) for **6**: Ni1−Si1 2.2654(6); C38−C39 1.344(3); Si1−C38 1.825(2); Ni1−C39 1.937(2); Ni1−C16 1.971(2); Ni1−C27 1.962(2); Ni1−Si1−C38 87.03(6); Si1‐Ni1‐C39 64.64(6); C39‐C38‐Si1 89.8(1); C38‐C39‐Ni1 118.4(2). Selected bond lengths (Å) and angles (°) for **7**: Ni1−Si1 2.2556(8); C38−C39 1.556(3); Si1−C38 1.870(2); Ni1−C39 2.015(2); Ni1−C16 1.953(2); Ni1−C27 1.945(2); Ni1‐Si1‐C38 89.83(8); Si1‐Ni1‐C39 66.19(7); C39‐C38‐Si1 85.7(1); C38‐C39‐Ni1 109.4(2).

The facile reaction of **1** with both ethylene and acetylene is reliant on the aforementioned LUMO+1 in this complex. That is, a DFT mechanistic analysis based on model complexes of **6** (Figure S46, Supporting Information) and **7** (Figure 3) suggests that both are formed through an initial η^2^‐complex at this nickel‐centered frontier orbital. This initial step is in fact reminiscent of that for the reaction of carbene–transition‐metal complexes with alkenes and alkynes in now well‐established multiple bond metathesis processes,^2^ and contrasts with the concerted [2+2] mechanism for reactions with polar substrates in the formation of **4** and **5**. Following this initial [2+1] cycloaddition, a spontaneous ring expansion to Si proceeds, generating the metallasila‐cyclobutane and ‐cyclobutene complexes **6** and **7**.^22^ Notably, complex **7** is only 2.5 kcal mol^−1^ lower in energy than **1**, with a 44.9 cal mol^−1^ K^−1^ entropic barrier to the formation of intermediary **IM1** (Figure 3). This is born out experimentally: despite the apparent C−C single bond present in the cyclic core of **7**, dissolution of pure crystals of this compound in C_6_D_6_ led to the generation of small amounts of both **1** and C_2_H_4_. Thus, to our surprise, the cycloaddition of ethylene to **1** is in fact reversible. Allowing a sample of **7** dissolved in C_6_D_6_ to stand for 24 h at ambient temperature led to the formation of a 1:1 mixture of **1** and a single new compound, **9** (see below), resulting from **1** reacting with two molar equiv of ethylene, given that no free ethylene could be observed in the ^1^H NMR spectrum of this mixture. Although a computational investigation for the reaction of **1** with ethylene suggested that **7** may readily undergo a further cycloaddition event with ethylene to form the six‐membered metallasilacycle **8** (Scheme 3, Figure 3), we were surprised to find that the experimentally observed product from the reaction of **1** with excess ethylene is the alkene–Ni^0^ π‐complex **9**.^23^ It seems likely that compound **9** is formed through a sequential Ni‐mediated β‐hydride elimination/reductive elimination reaction from intermediary **8** (Scheme 3).^24^ Circumstantial evidence for the intermediate generation of **8** came from the isolation of one or two crystals of this compound by the low‐temperature reaction of **1** with excess ethylene, followed by storage at −30 °C for two weeks. Although this compound was highly unstable, allowing only for the collection of preliminary crystallographic data, the molecular structure, ascertaining the connectivity in **8**, is shown in Figure S22 in the Supporting Information.

**Figure 3:**
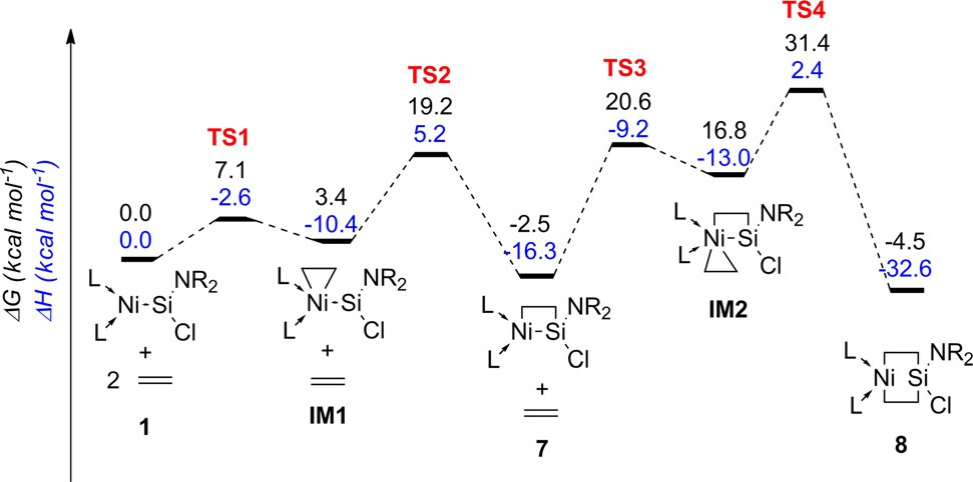
DFT‐derived reaction pathway for the addition of two molar equiv ethylene to **1**. L=[:C{N(Me)CH}_2_]; NR_2_=[(Me)(SiMe_3_)N].

**Scheme 3:**
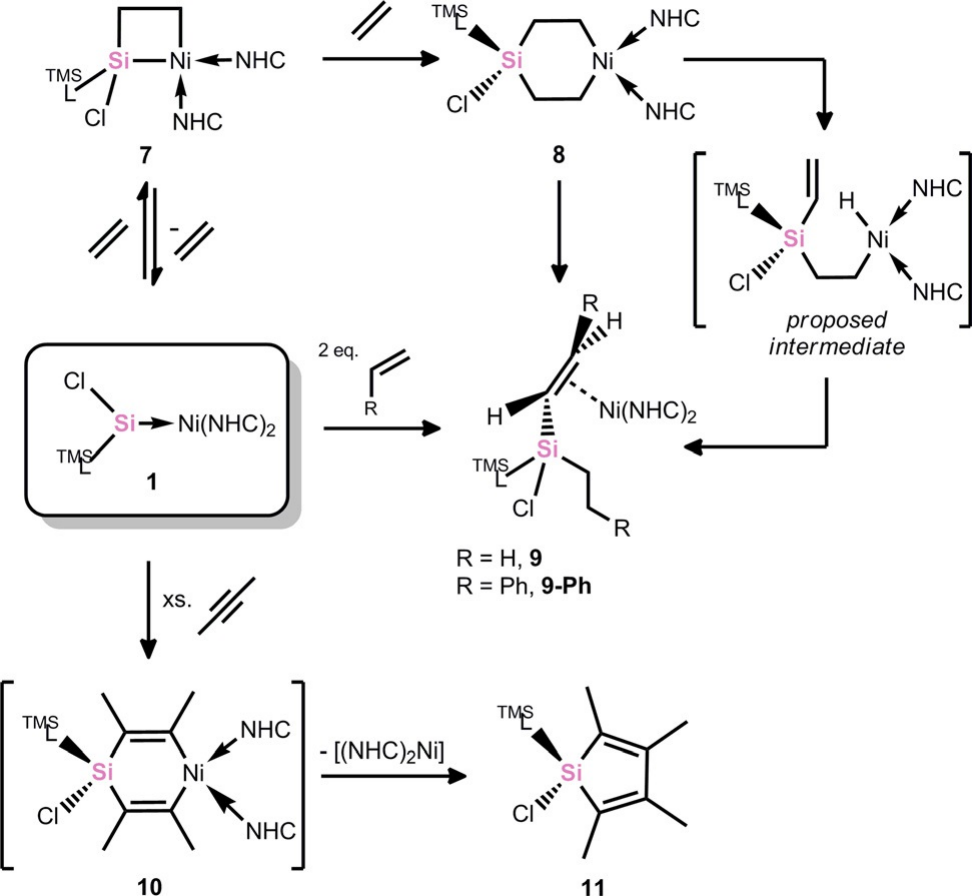
The reversible addition of ethylene to **1**, and C−H activation reactions upon double addition of alkenes to **1**.

The molecular structure of **9** contains a silyl‐substituted ethylene unit in the coordination sphere of Ni^0^, the silyl group bearing the ^TMS^L, Cl, and Et ligands. The C−C and Ni−C bonds in the cyclic core of **9** are in keeping with those in related alkene–Ni π‐complexes. The ^1^H NMR spectrum of **9** in C_6_D_6_ is very complex, both due to the asymmetrical substitution at the alkene and the silyl center, leading to diasterotopic proton couplings (Figures S26 and S27, Supporting Information). This does, however, further confirm the connectivity in this species. The two Si^IV^ centers yield very similar resonances in the ^29^Si NMR spectrum of **9** at *δ*=5.2 and 7.8 ppm, the latter corresponding to the formerly Si^II^ center as shown through a two‐dimensional ^1^H,^29^Si HMQC NMR experiment (Figure S31). Notably, this reaction was shown to be reproducible for other alkenes, as shown by the formation of alkene π‐complex **9‐Ph** in the reaction of **1** with two molar equivs. of styrene. The molecular structure of **9‐Ph** is essentially isostructural to that for **9** (Figure S44), and is therefore also similar to previously reported alkene–Ni π‐complexes. It is worthy of note that the *trans*‐conformation in **9‐Ph** is exclusively formed, most likely due to steric interactions between its silyl and phenyl substituents. These reactions, and particularly that with ethylene, whose C−H bonds are relatively inert, perhaps point towards potential synthetic applications for these remarkable cycloaddition reactions, particularly when one notes that an asymmetric silicon center is generated.

Synthetic utility of the described cycloaddition reactions was further displayed when **1** was reacted with an excess of 2‐butyne, which proceeded through the reductive elimination of silole **11** (Scheme 3, Figure 4), similarly to a previously reported 6‐membered platinasilacycle which eliminates silole when heated to 120 °C.^25^

**Figure 4:**
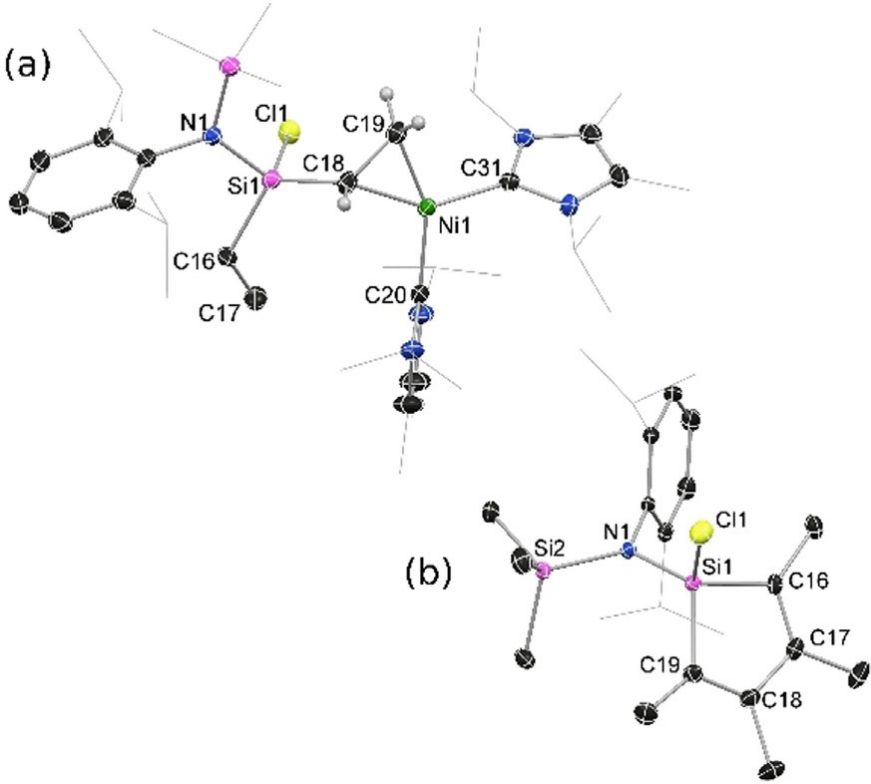
The molecular structure of (a) **9**, and (b) **10**, with thermal ellipsoids at 30 % probability. Selected bond lengths (Å) and angles (°) for **9**: Ni1−C18 1.988(3); Ni1−C19 1.924(3); C18−C19 1.448(4); Si1−C16 1.915(5); Si1−C18 1.809(3); C16−C17 1.519(7); C18‐Ni1‐C19 43.4(1); C18‐C19‐Ni1 70.6(2); C19‐C18‐Ni1 66.0(1); C19‐C18‐Si1 124.9(2). Selected bond lengths (Å) and angles (°) for **10**: Si1−C16 1.847(2); Si1−C19 1.854(2); C16−C17 1.352(2); C17−C18 1.504(3); C18−C19 1.347(2); C16‐Si1‐C19 95.27(8); C17‐C16‐Si1 105.4(1); C18‐C19‐Si1 105.1(1); C16‐C17‐C18 116.8(2); C17‐C18‐C19 117.4(2).

Nevertheless, such heterocycles are typically not obtained in the direct reactions of silylenes with acetylene derivatives, for which the [2+1] reaction products are more commonly encountered.^26^, ^27^ The molecular structure of **11** is in agreement with previously reported siloles, containing a planar SiC_4_ ring and two short C=C bonds (*d*(C16−C17)=1.352(2) Å; *d*(C18−C19)=1.347(2) Å). As with the formation of C−H activation products **9** and **9‐Ph**, heterocycle **11** is likely formed through a [2+2+2] cycloaddition reaction of the Si−Ni bond in **1** with two molar equivalents of 2‐butyne, proceeding via 1‐metalla‐4‐sila‐cyclohexadiene derivative **10**. Indeed, it has previously been shown that platina‐sila‐cyclobutene species can undergo such a ring‐expansion reaction in the presence of excess alkyne, albeit without silole elimination.21c A DFT investigation employing acetylene in place of 2‐butyne suggests that this is the most energetically favored reaction coordinate, with the acetylene derivative of **10** (i.e. **IM3“**, Figure S46 in the Supporting Information) lying 55.6 kcal mol^−1^ lower in energy than **1** and free acetylene. Reductive elimination and formation of a π‐complex of the liberated silole derivative (**IM4“**, Figure S46) is further favored by 27.9 kcal mol^−1^.

In summary, we have investigated the [2+2] cycloaddition chemistry of the Si−Ni multiple bond in **1** towards unsaturated organic compounds, leading to a range of four‐membered nickelasilacycles, including rare examples of metallasilacyclobutene species. Markedly, we have found that the addition of ethylene is reversible, whereas the reaction with excess ethylene proceeds through a [2+2+2] cycloaddition reaction, leading finally to sp^2^‐CH bond activation. Further, the liberation of fragments containing newly formed C−C bonds has also been shown possible, reminiscent of key intermediary steps in established catalytic processes.

## Conflict of interest

The authors declare no conflict of interest.

## Supporting information

As a service to our authors and readers, this journal provides supporting information supplied by the authors. Such materials are peer reviewed and may be re‐organized for online delivery, but are not copy‐edited or typeset. Technical support issues arising from supporting information (other than missing files) should be addressed to the authors.

Supplementary
