# Supplementary material for: Cycloaddition Chemistry of a Silylene‐Nickel Complex toward Organic π‐Systems: From Reversibility to C−H Activation
Source: Chemistry. 2020 Jan 31;26(9):1958–62. doi: 10.1002/chem.202000009 (PMC7028101; doi:10.1002/chem.202000009)
Supplement: Supplementary file 1 — Supplementary [file CHEM-26-1958-s001.pdf]

CHEMISTRY
A European Journal

Supporting Information

Cycloaddition Chemistry of a Silylene-Nickel Complex toward Organic π-Systems: From Reversibility to C–H Activation

Terrance J. Hadlington,* Arseni Kostenko, and Matthias Driess*[a]

chem_202000009_sm_miscellaneous_information.pdf

Supporting Information

Contents:

1. **Experimental methods and data…………………2**
2. **X-Ray crystallographic data……………………...29**
3. **Computational methods and data……………….33**

1. Experimental methods and data

General considerations. All experiments and manipulations were carried out under dry oxygen free dinitrogen using standard Schlenk techniques or in an MBraun inert atmosphere glovebox containing an atmosphere of high purity dinitrogen. Hexane, diethylether, toluene and THF were dried by standard methods. C_6D_6 and D_8-THF were stirred over a sonicated potassium mirror for a period of 48 hr and recondensed into a Schlenk tube containing activated 4 Å mol sieves. DCM-d_2 was stirred over CaH_2 for 24 hr and distilled into a Schlenk tube containing activated 4 Å mol sieves. NMR spectra were recorded on a Bruker AV 200, 400, or 500 Spectrometer. The ^{1}H and ^{13}C{^{1}H} NMR spectra were referenced to the residual solvent signals as internal standards. ^{29}Si NMR spectra were externally calibrated with $SiMe_4$. The starting material [{N(Dipp)($SiMe_3$)}ClSi:→Ni(NHC)$_2$] (**1**; ^{TMS}L = [(Dipp)($SiMe_3$)N]$^-$; Dipp = C_6H_3-Pr^i-2,6; NHC = [:C{N(Pr^i)C(Me)}$_2$]) was synthesized according to the known literature procedure.[1] All other reagents were used as received.

Synthesis of 2. A solution of **1** (0.2 g, 0.27 mmol) in toluene (10 mL) was cooled to -78 °C, and phenyl acetylene added *via* pipette (30 μL, 0.27 mmol). An immediate color change to deep purple-brown was observed. The reaction mixture was subsequently warmed to ambient temperature, whereupon all volatiles were removed *in vacuo*. The reaction mixture was extracted in warm hexane (15mL), concentrated to ~2 mL, and stored at 4 °C for 2 weeks to afford a crop of deep red-purple crystalline **2** (160 mg, 70 %). ^{1}H NMR (C_6D_6, 400 MHz, 298 K): δ = 0.41 (s, 9H, Si*Me*$_3$), 1.09 (br, 18H, NHC-Pr^i-C*H*$_3$), 1.35 (overlapping d, 9H, Dipp-Pr^i-C*H*$_3$), 1.45 (d, $^3J_{HH}$ = 6.8 Hz, 3H, Dipp-Pr^i-C*H*$_3$), 1.53 (br, 6H, NHC-Pr^i-C*H*$_3$) 1.76 (s, 6H, NHC-NC*Me*), 1.81 (s, 6H, NHC-NC*Me*), 3.98 (sept, $^3J_{HH}$ = 6.8 Hz, 1H, Dipp-Pr^i-C*H*), 4.09 (sept, $^3J_{HH}$ = 6.8 Hz, 1H, Dipp-Pr^i-C*H*), 5.81 (br, 2H, NHC-Pr^i-C*H*), 5.85 (sept, $^3J_{HH}$ = 7.2 Hz, 2H, NHC-Pr^i-C*H*), 6.89 (m, 1H, Ar-C*H*), 7.06 (m, 4H, Ar-C*H*), 7.21 (m, 3H, Ar-C*H*); ^{13}C{^{1}H} NMR (C_6D_6, 75.5 MHz, 298 K): δ = 2.6 (Si*Me*$_3$), 10.4 and 10.5 (NHC-NC*Me*), 21.9, 22.7, and 23.1 (br, NHC-Pr^i-CH$_3$), 24.8, 25.4, 25.7, and 26.5 (Dipp-Pr^i-*C*H$_3$), 28.3 and 28.4 (Dipp-Pr^i-*C*H), 52.4 and 52.5 (NHC-Pr^i-*C*H), 124.3, 124.4, 125.5, and 125.6 (NHC-N*C*Me), 123.8, 127.1, 127.7, 128.4, 131.0, 135.5, 141.7, 148.2, and 168.2 (Ar-*C*), 200.7 and 200.8 (NHC-*C*:); ^{29}Si{^{1}H} NMR (C_6D_6, 80 MHz, 298 K): δ = 6.4 (*Si*Me$_3$), -32.9 (H-*Si*-L^{TMS}); anal. calcd. for $C_{45}H_{72}ClN_5NiSi_2$: C, 64.85 %; H, 8.71 %; N, 8.40 %; found: C, 64.58 %; H, 9.08%; N, 8.30 %.

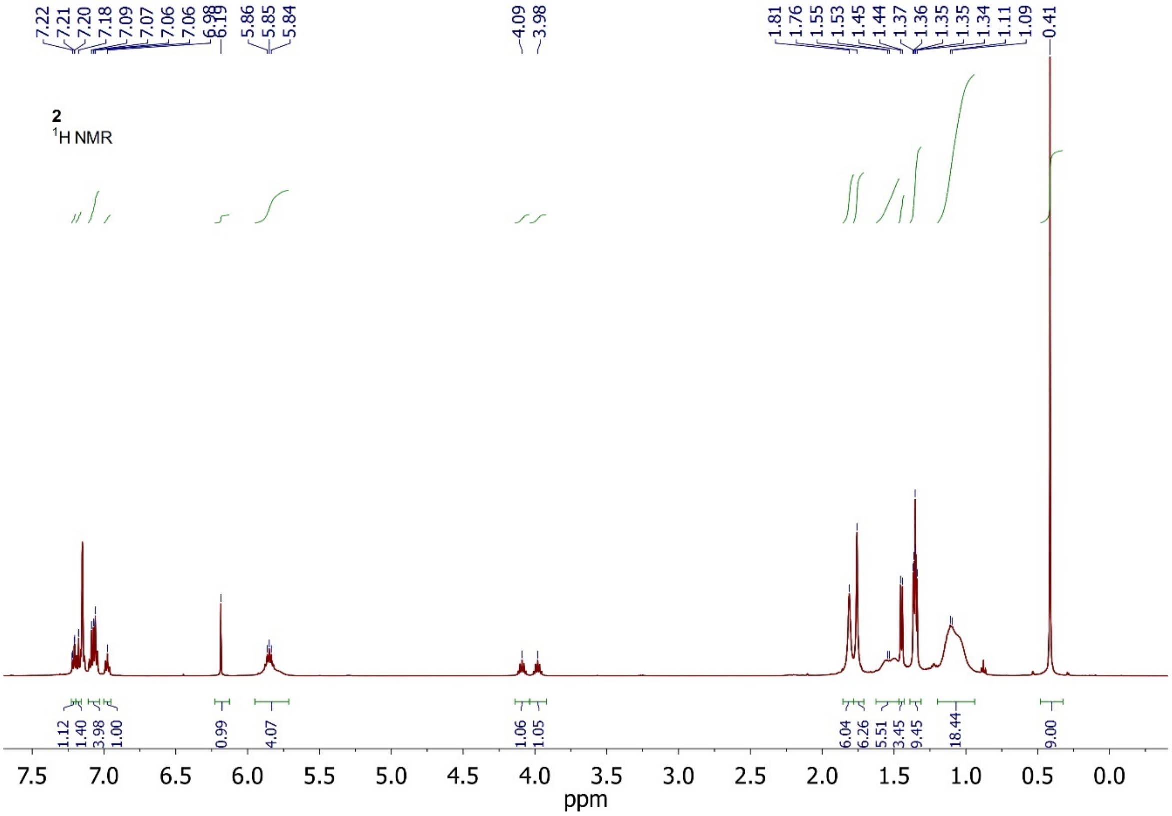

Figure S1. ^{1}H NMR spectrum of **2** dissolved in C_6D_6, at 298K.

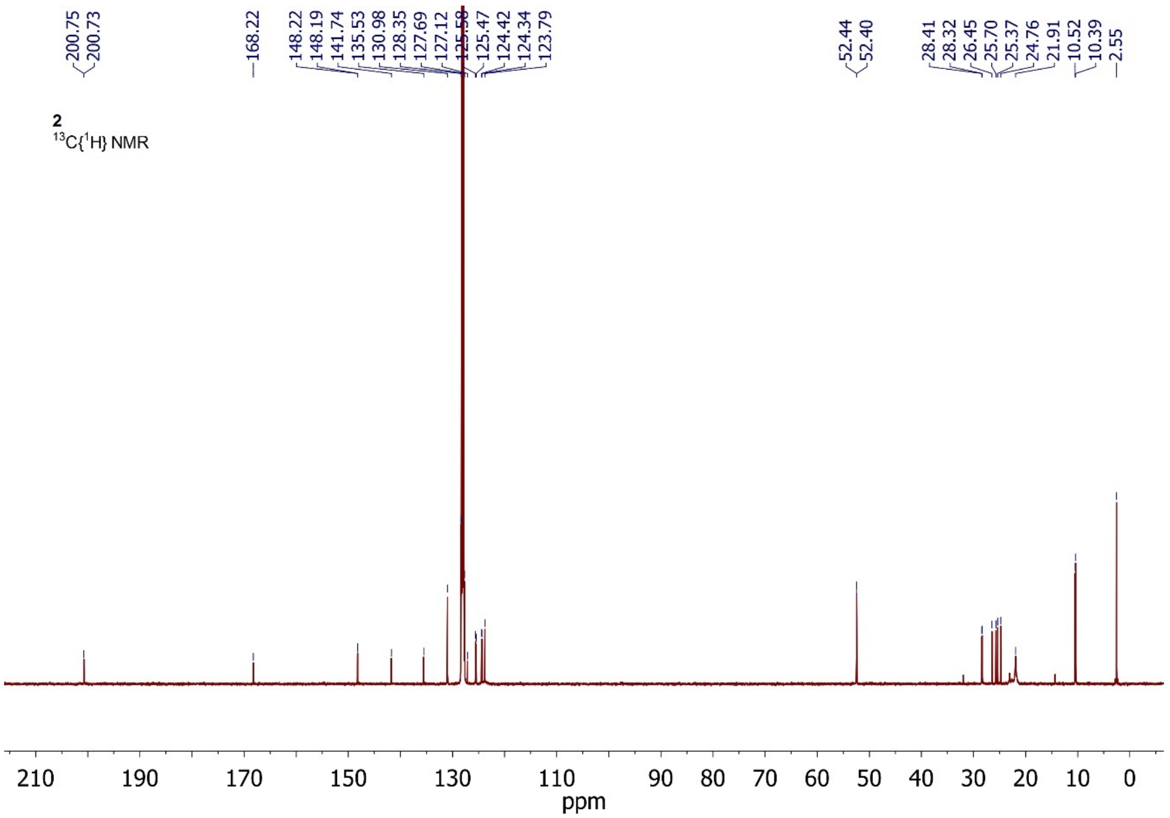

Figure S2. $^{13}C\{^1H\}$ NMR spectrum of **2** dissolved in C_6D_6, at 298K.

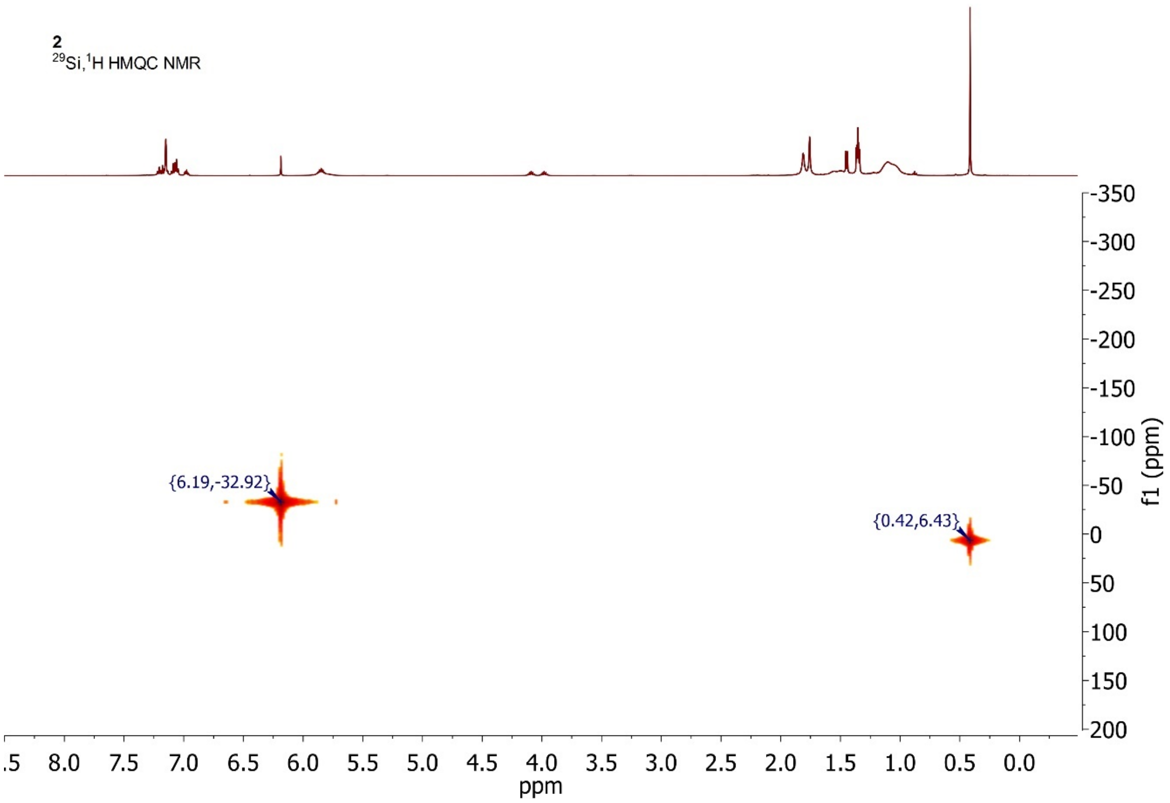

Figure S3. $^{29}Si,^{1}H$ HMQC NMR spectrum of **2** dissolved in C_6D_6, at 298K.

Synthesis of 3. A solution of **1** (0.2 g, 0.27 mmol) in toluene (10 mL), was cooled to -78 °C, and acetophenone added *via* pipette (32 μL, 0.27 mmol), leading to an immediate color change to orange-yellow. The reaction was warmed to 0 °C, and all volatiles removed *in vacuo*. The residue was extracted in hexane, filtered, and concentrated to 5 mL. Storage of this solution at ambient temperature led to the formation of a large crop of yellow crystals of **3** over the course of 1 h (150 mg, 65 %). Storage of the solution at this temperature for >2 h periods leads to decomposition of the product. ^{1}H NMR (D_8-THF , 400 MHz, 243 K): δ = -11.71 (s, 1H, Ni-*H*), 0.25 (s, 9H, Si*Me₃*), 0.94 (d, $^{3}J_{HH}$ = 6.8 Hz, 6H, Dipp-Pr^{i}-CH_3), 0.98-1.34 (overlapping d/br, 27H, Pr^{i}-CH_3), 1.47 (br d, $^{3}J_{HH}$ = 6.8 Hz, 3H, Dipp-Pr^{i}-CH_3), 1.55 (br d, $^{3}J_{HH}$ = 6.8 Hz, 3H, Dipp-Pr^{i}-CH_3), 2.16 (s, 6H, NHC-NC*Me*), 2.18 (s, 6H, NHC-NC*Me*), 2.22 (s, 6H, NHC-NC*Me*), 3.97 (br, 2H, Dipp-Pr^{i}-C*H*), 4.57 (br, 1H, Ni-η^{2}-(CH_2C*H*-Si)), 5.20-6.00 (br, 4H, NHC-Pr^{i}-C*H*), 5.63 (br, 2H, Ni-η^{2}-(CH_2C*H*-Si)), 6.88-7.11 (m, 8H, Ar-C*H*); $^{13}C\{^{1}H\}$ NMR (D_8-THF, 75.5 MHz, 243 K): δ = 5.0 (Si*Me₃*), 10.5, and 10.6 (NHC-NC*Me*), 21.5, 21.7, 22.4, and 23.07 (br/overlapping, NHC-Pr^{i}-CH_3), 25.9, 26.2, 26.5, and 26.9 (Dipp-Pr^{i}-CH_3), 28.4, and 28.7 (Dipp-Pr^{i}-*C*H), 53.8, 53.9, and 54.3 (br, NHC-Pr^{i}-*C*H), 91.3 (Ph-C=CH_2), 125.5, and 127.3 (NHC-N*C*Me), 124.2, 124.3, 125.6, 126.6, 127.9, 140.6, 145.4, 148.1, and 148.2 (Ar-*C*), 156.6 (Ph-*C*=CH_2), 192.1, and 194.2 (NHC-*C*:); $^{29}Si\{^{1}H\}$ NMR (D_8-THF, 80 MHz, 243 K): δ = 1.6 (*Si*Me₃), -18.7 (O-*Si*-Ni); anal. calcd. for $C_{45}H_{74}ClN_5NiOSi_2$: C, 63.48 %; H, 8.76 %; N, 8.23 %; found: C, 63.36 %; H, 8.76 %; N, 8.03 %.

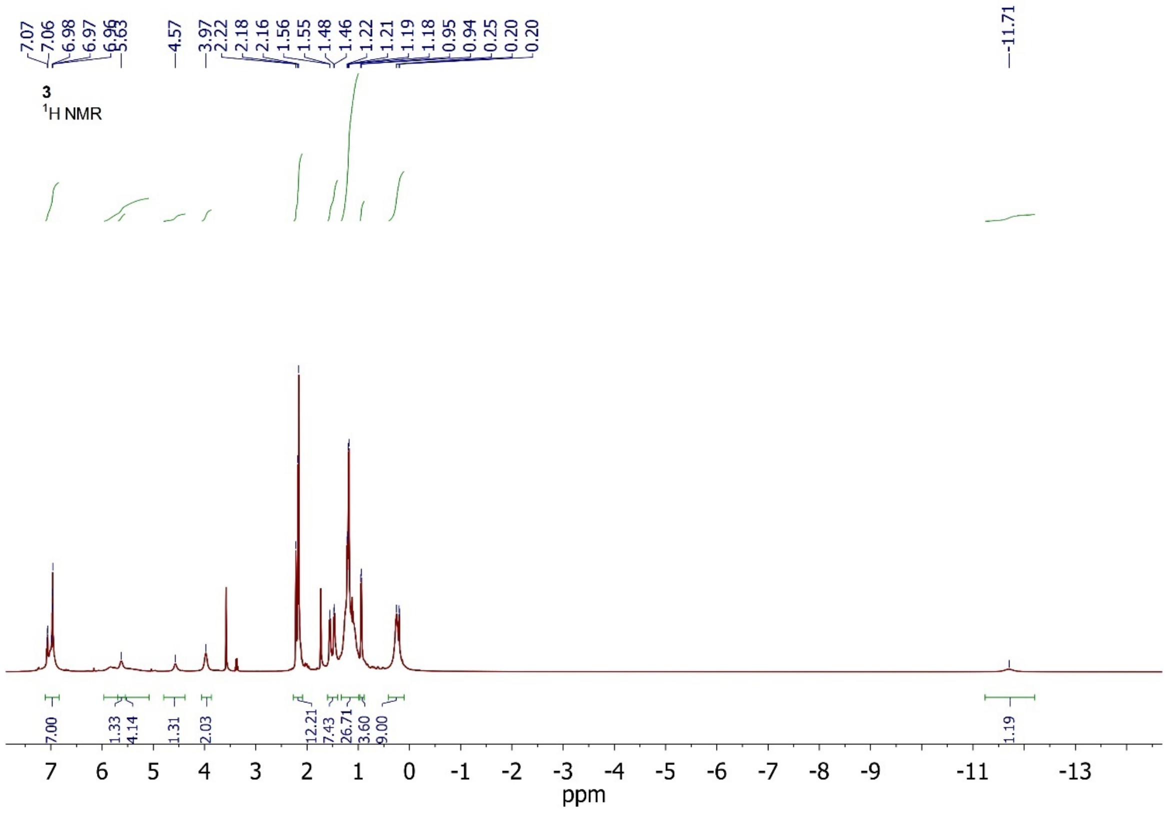

Figure S4. ^{1}H NMR spectrum of **3** dissolved in D_8-THF, at 243K.

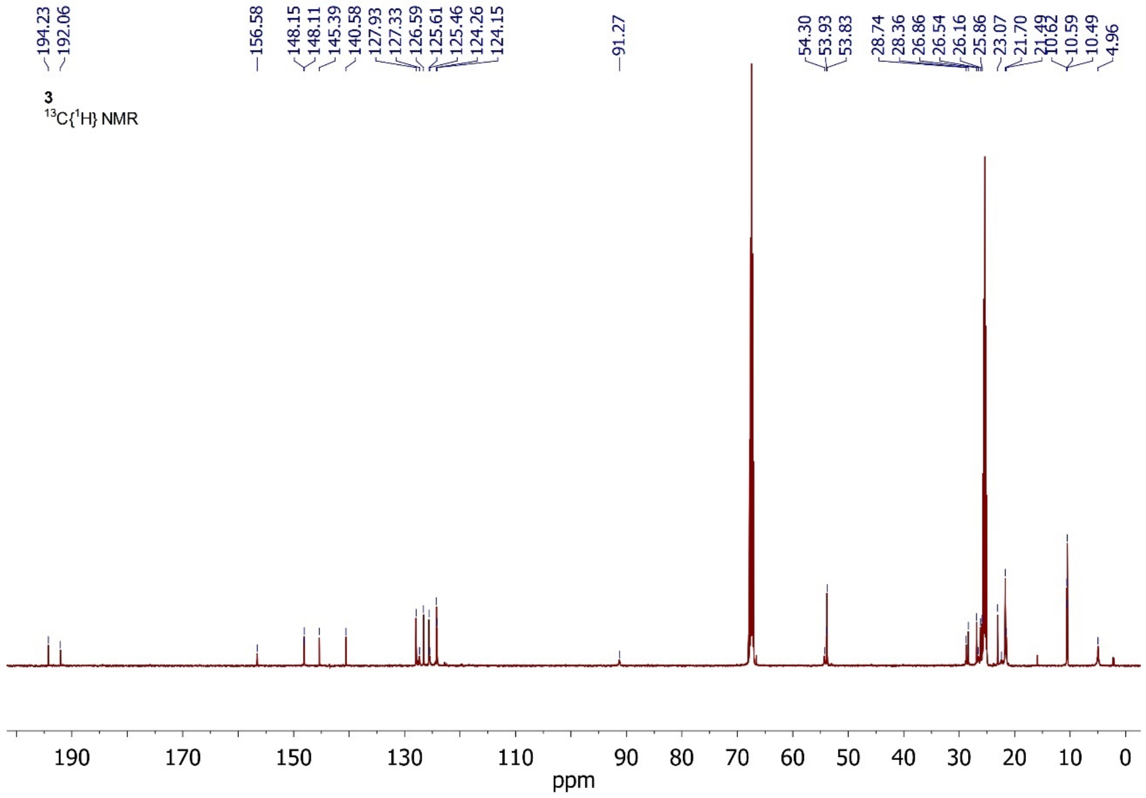

Figure S5. $^{13}C\{^{1}H\}$ NMR spectrum of **3** dissolved in D_8-THF, at 243K.

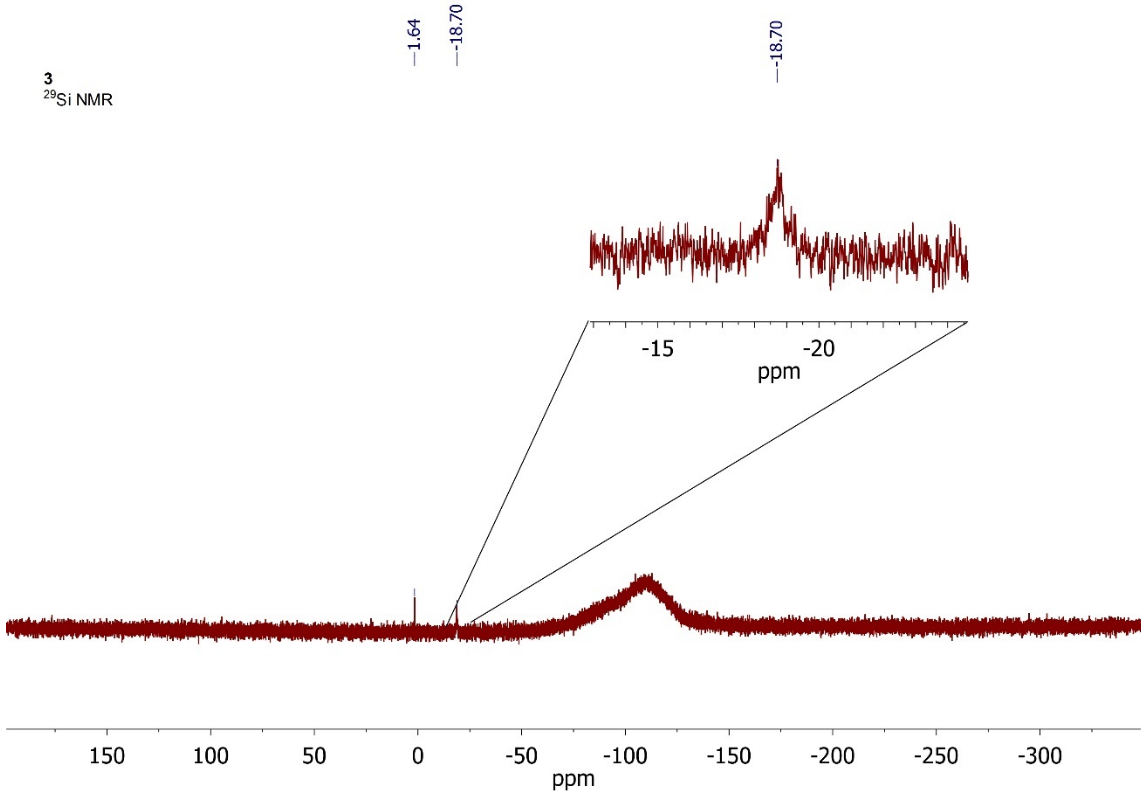

Figure S6. $^{29}Si\{^1H\}$ NMR spectrum of **3** dissolved in D_8-THF, at 243K.

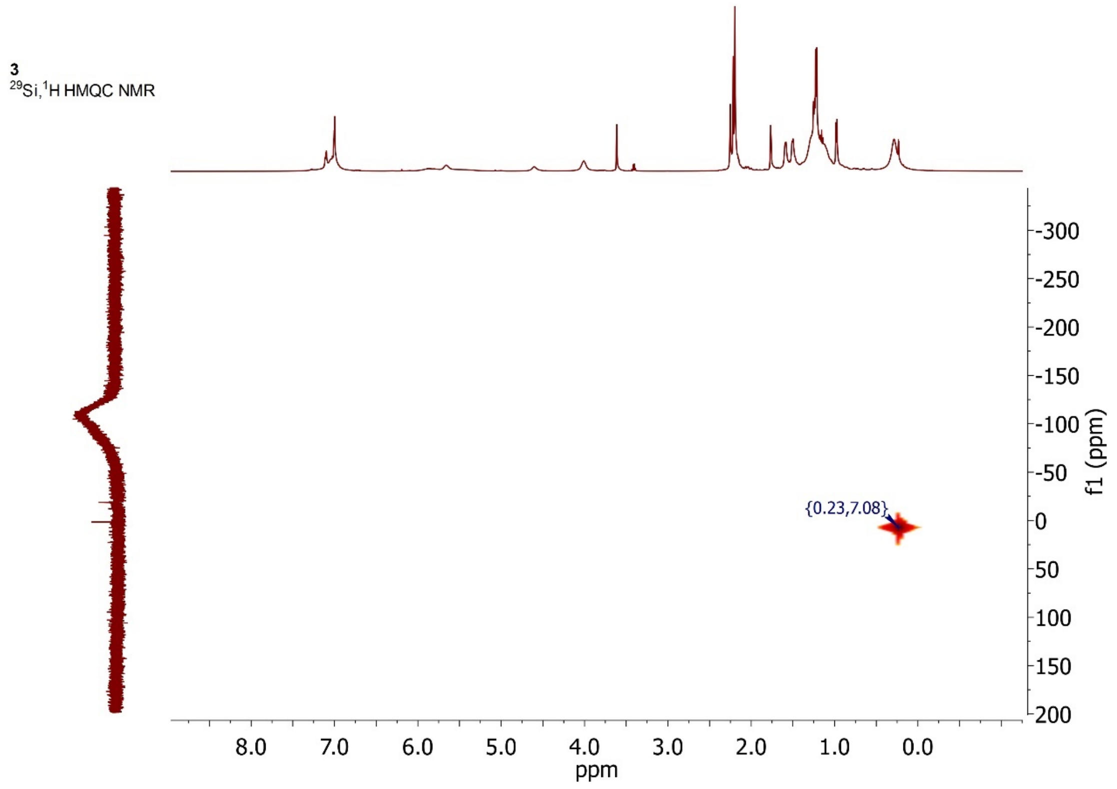

Figure S7. $^{29}Si,^{1}H$ HMQC NMR spectrum of **3** dissolved in D_8-THF, at 243K.

Synthesis of 4. A solution of **1** (0.15 g, 0.21 mmol) in toluene (10 mL) was cooled to -78 °C, and 4-trifluoromethyl benzaldehyde added *via* pipette (25 μL, 0.21 mmol), leading to an immediate color change to orange-yellow. The reaction was warmed to 0 °C, and all volatiles removed *in vacuo*. The residue was extracted in hexane, filtered, and concentrated to 5 mL. Storage of this solution at ambient temperature overnight led to the formation of a large crop of orange crystals of **4** (80 mg, 46 %). ^{1}H NMR (D_8-THF, 400 MHz, 298 K): δ = 0.35 (s, 9H, Si*Me$_3$*), 0.39 (d, $^{3}J_{HH}$ = 7.2 Hz, 3H, NHC-Pr^{i}-C*H*$_3$), 0.59 (overlapping d, $^{3}J_{HH}$ = 7.2 Hz, 6H, NHC-Pr^{i}-C*H*$_3$), 0.82 (d, $^{3}J_{HH}$ = 6.8 Hz, 3H, Dipp-Pr^{i}-C*H*$_3$), 1.11 (d, $^{3}J_{HH}$ = 6.8 Hz, 3H, Dipp-Pr^{i}-C*H*$_3$), 1.16 (d, $^{3}J_{HH}$ = 6.8 Hz, 3H, Dipp-Pr^{i}-C*H*$_3$), 1.21 (d, $^{3}J_{HH}$ = 6.8 Hz, 3H, Dipp-Pr^{i}-C*H*$_3$), 1.40 (d, $^{3}J_{HH}$ = 7.2 Hz, 3H, NHC-Pr^{i}-C*H*$_3$), 1.47 (overlapping d, $^{3}J_{HH}$ = 7.2 Hz, 6H, NHC-Pr^{i}-C*H*$_3$), 1.74 (d, $^{3}J_{HH}$ = 7.2 Hz, 3H, NHC-Pr^{i}-C*H*$_3$), 1.85 (d, $^{3}J_{HH}$ = 7.2 Hz, 3H, NHC-Pr^{i}-C*H*$_3$), 2.04 (s, 12H, NHC-NC*Me*), 2.08 (s, 12H, NHC-NC*Me*), 2.15 (s, 12H, NHC-NC*Me*), 2.34 (s, 12H, NHC-NC*Me*), 3.57 (sept, $^{3}J_{HH}$ = 6.8 Hz, 1H, Dipp-Pr^{i}-C*H*), 3.78 (sept, $^{3}J_{HH}$ = 6.8 Hz, 1H, Dipp-Pr^{i}-C*H*), 4.52 (sept, $^{3}J_{HH}$ = 7.2 Hz, 1H, NHC-Pr^{i}-C*H*), 5.08 (s, 1H, Si-OC(*H*)(Ph)-Ni), 5.28 (sept, $^{3}J_{HH}$ = 7.2 Hz, 1H, NHC-Pr^{i}-C*H*), 6.53 (sept, $^{3}J_{HH}$ = 7.2 Hz, 1H, NHC-Pr^{i}-C*H*), 6.80 (sept, $^{3}J_{HH}$ = 7.2 Hz, 1H, NHC-Pr^{i}-C*H*), 6.90 (m, 2H, Dipp-*m*-Ar-C*H*), 6.95 (m, 1H, Dipp-*p*-Ar-C*H*), 7.12 and 7.21 (m, 4H, CF_3-Benzlald.-Ar-C*H*); ^{13}C{^{1}H} NMR (D_8-THF, 75.5 MHz, 298 K): δ = 4.0 (Si*Me*$_3$), 10.3, 10.6, 10.7 and 10.8 (NHC-NC*Me*), 20.3, 20.9, 21.0, 21.3, 22.6, and 23.1 (NHC-Pr^{i}-*C*H$_3$), 24.1 and 25.6 (Dipp-Pr^{i}-*C*H$_3$), 28.5 and 28.7 (Dipp-Pr^{i}-*C*H), 53.0, 53.5, 53.6, and 53.9 (NHC-Pr^{i}-*C*H), 123.4, 123.9, 124.3, and 125.3 (NHC-N*C*Me), 124.8 (q, Ar-*C*F$_3$), 125.9, 126.0, 126.9, 127.8, 144.6, 147.1, 148.7, and 159.1 (Ar-*C*), 187.5 and 190.5 (NHC-*C*:); ^{19}F NMR (D_8-THF, 188 MHz, 298 K): δ = 64.3; anal. calcd. for $C_{45}H_{71}ClF_3N_5NiOSi_2$: C, 59.70 %; H, 7.90 %; N, 7.74 %; found: C, 59.84 %; H, 8.19 %; N, 7.43 %.

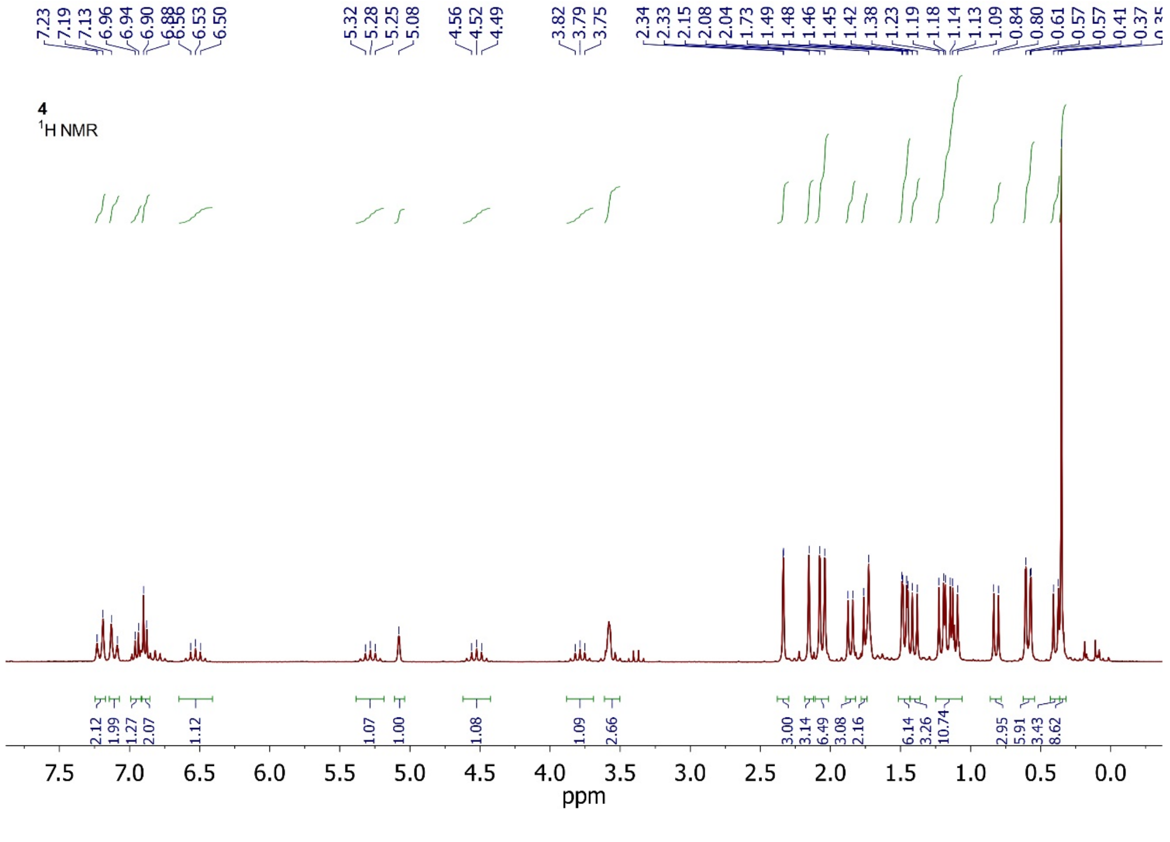

Figure S8. ^{1}H NMR spectrum of **4** dissolved in D_8-THF, at 298K.

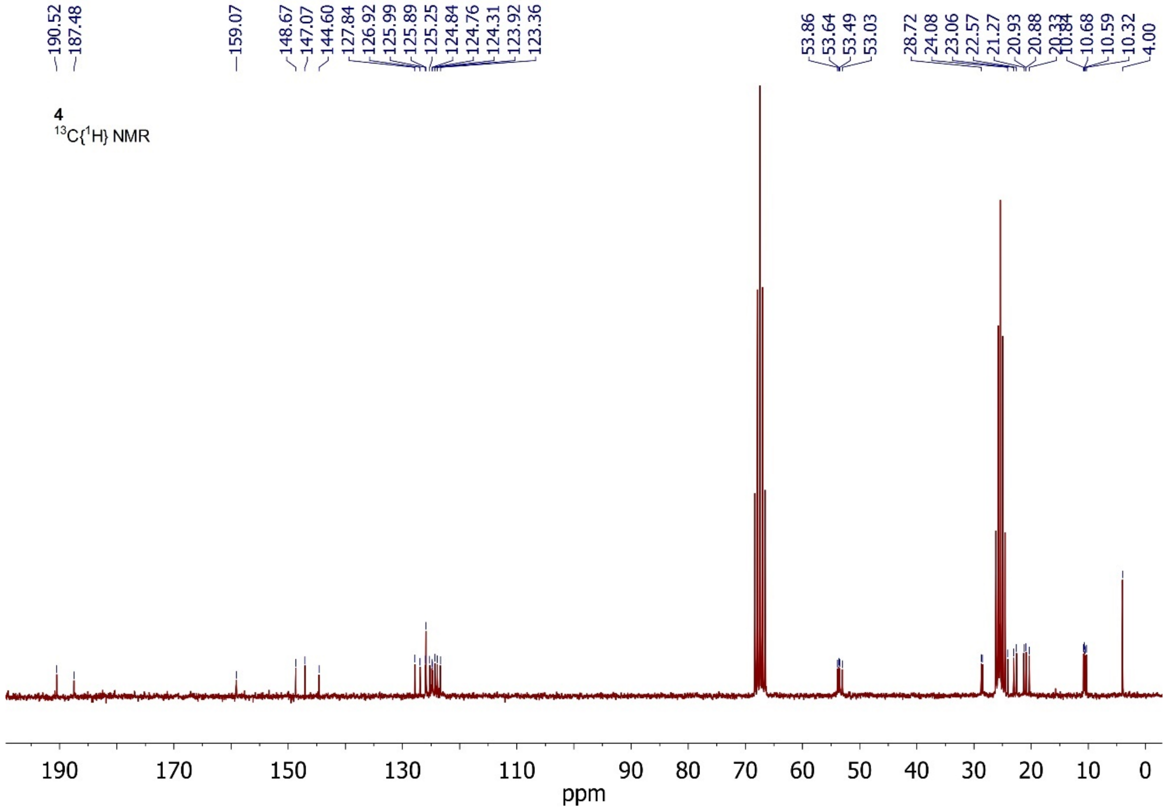

Figure S9. ^{13}C{^{1}H} NMR spectrum of **4** dissolved in D_8-THF, at 298K.

Synthesis of 5. A solution of **1** (0.2 g, 0.27 mmol) in toluene (10 mL), was cooled to -78 °C, and N-benzylideneaniline added as a solid (49.7 mg, 0.27 mmol). A color change to orange-yellow was observed after stirring for 2 h with warming to ambient temperature. At this stage, all volatiles were removed from the reaction mixture *in vacuo*, the solid residue extracted in diethyl ether (25 mL), and filtered. Concentration of this solution to 15 mL and storage at 4 °C overnight resulted in the formation of large crop of red-orange crystals of **5** (110 mg, 45 %). ^{1}H NMR (D_8-THF, 400 MHz, 298 K): δ = 0.02 (br, 3H, NHC-Pr^{i}-CH_3), 0.26 (s, 9H, SiMe_3), 0.32 (d, $^{3}J_{HH}$ = 7.2 Hz, 3H, NHC-Pr^{i}-CH_3), 0.45 (d, $^{3}J_{HH}$ = 7.2 Hz, 3H, NHC-Pr^{i}-CH_3), 0.51 (d, $^{3}J_{HH}$ = 7.2 Hz, 3H, NHC-Pr^{i}-CH_3), 0.88 (d, $^{3}J_{HH}$ = 6.8 Hz, 3H, Dipp-Pr^{i}-CH_3), 1.00 (d, $^{3}J_{HH}$ = 6.8 Hz, 3H, Dipp-Pr^{i}-CH_3), 1.14 (d, $^{3}J_{HH}$ = 6.8 Hz, 3H, Dipp-Pr^{i}-CH_3), 1.46 (d, $^{3}J_{HH}$ = 6.8 Hz, 3H, Dipp-Pr^{i}-CH_3), 1.50 (d, $^{3}J_{HH}$ = 7.2 Hz, 3H, NHC-Pr^{i}-CH_3), 1.55 (d, $^{3}J_{HH}$ = 7.2 Hz, 3H, NHC-Pr^{i}-CH_3), 1.77 (d, $^{3}J_{HH}$ = 7.2 Hz, 3H, NHC-Pr^{i}-CH_3), 1.99 (d, $^{3}J_{HH}$ = 7.2 Hz, 3H, NHC-Pr^{i}-CH_3), 2.01 (s, 3H, NHC-NC*Me*), 2.19 (s, 3H, NHC-NC*Me*), 2.27 (s, 3H, NHC-NC*Me*), 2.36 (s, 3H, NHC-NC*Me*), 3.70 (sept, $^{3}J_{HH}$ = 6.8 Hz, 1H, Dipp-Pr^{i}-C*H*), 3.89 (sept, $^{3}J_{HH}$ = 6.8 Hz, 1H, Dipp-Pr^{i}-C*H*), 4.31 (s, 1H, Si-N(Ph)C(*H*)(Ph)-Ni), 4.43 (sept, $^{3}J_{HH}$ = 7.2 Hz, 1H, NHC-Pr^{i}-C*H*), 5.29 (sept, $^{3}J_{HH}$ = 7.2 Hz, 1H, NHC-Pr^{i}-C*H*), 6.18 (m, 1H, Ar-C*H*), 6.56 (m, 5H, Ar-C*H*), 6.78 (overlapping sept, 1H, NHC-Pr^{i}-CH_3), 6.79-6.94 (m, 7H, Ar-C*H*), 7.00 (overlapping sept, 1H, NHC-Pr^{i}-CH_3); ^{13}C{^{1}H} NMR (D_8-THF, 75.5 MHz, 298 K): δ = 4.1 (SiMe_3), 10.4, 10.7, 10.8, and 10.9 (NHC-NC*Me*), 20.6, 20.9, 21.0, 21.5, 22.0, 22.6, 23.5, and 24.6 (NHC-Pr^{i}-*C*H_3), 25.9, 26.0, 26.2, and 26.8 (Dipp-Pr^{i}-*C*H_3), 28.1 and 28.5 (Dipp-Pr^{i}-*C*H), 45.6 (PhN-*C*(H)(Ph)), 53.1, 53.6, 53.7, and 54.0 (NHC-Pr^{i}-

*C*H), 125.6, 125.8, 126.6, and 126.8 (NHC-N*C*Me), 116.6, 119.3, 122.3, 124.1, 124.4, 124.5, 124.7, 127.8, 127.9, 128.0, 145.0, 147.5, 148.4, 148.7, 150.6, and 153.4 (Ar-*C*), 188.8 and 191.7 (NHC-*C*:); ^{29}Si{^{1}H} NMR (D_8-THF, 80 MHz, 298 K): δ = 2.4 (*Si*Me_3), -65.4 (Ni-C(H)(Ph)-N(Ph)-*Si*); anal. calcd. for $C_{50}H_{77}ClN_6NiSi_2$: C, 65.81 %; H, 8.51 %; N, 9.21 %; found: C, 64.65 %; H, 8.35 %; N, 9.12 %.

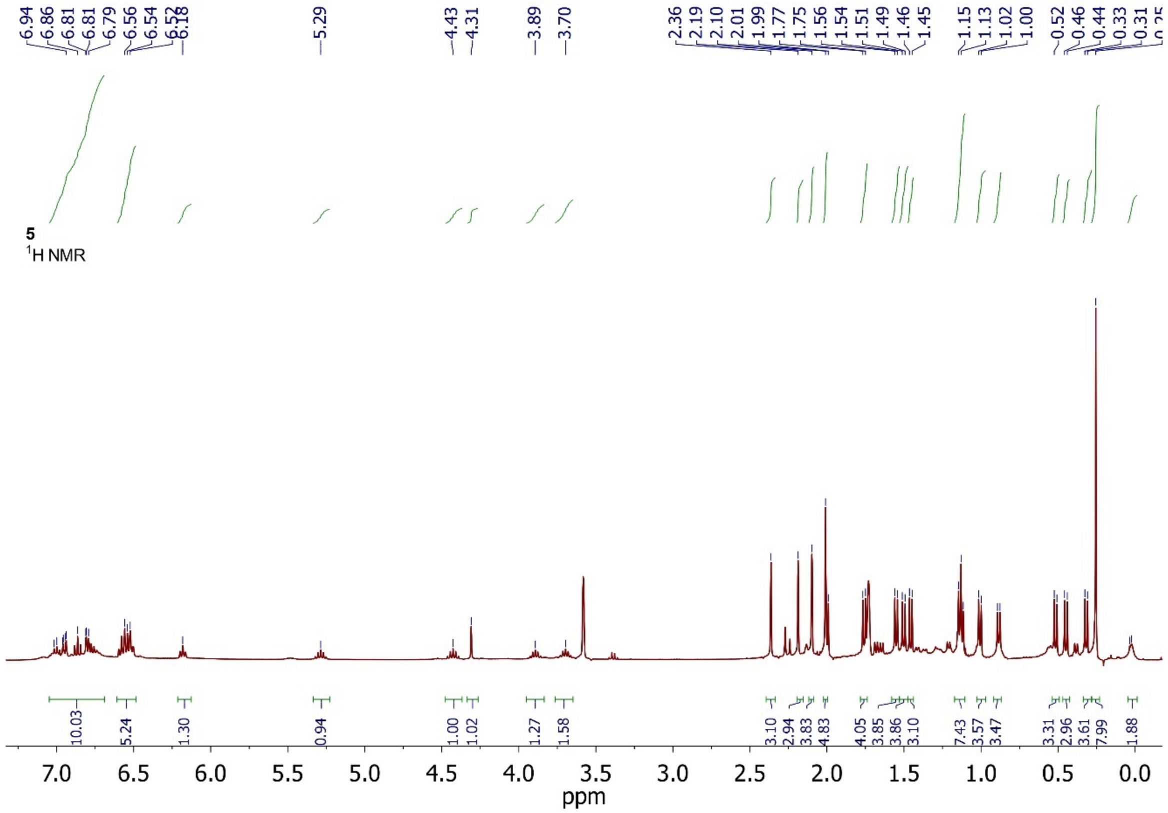

Figure S10. ^{1}H NMR spectrum of **5** dissolved in D_8-THF, at 298K.

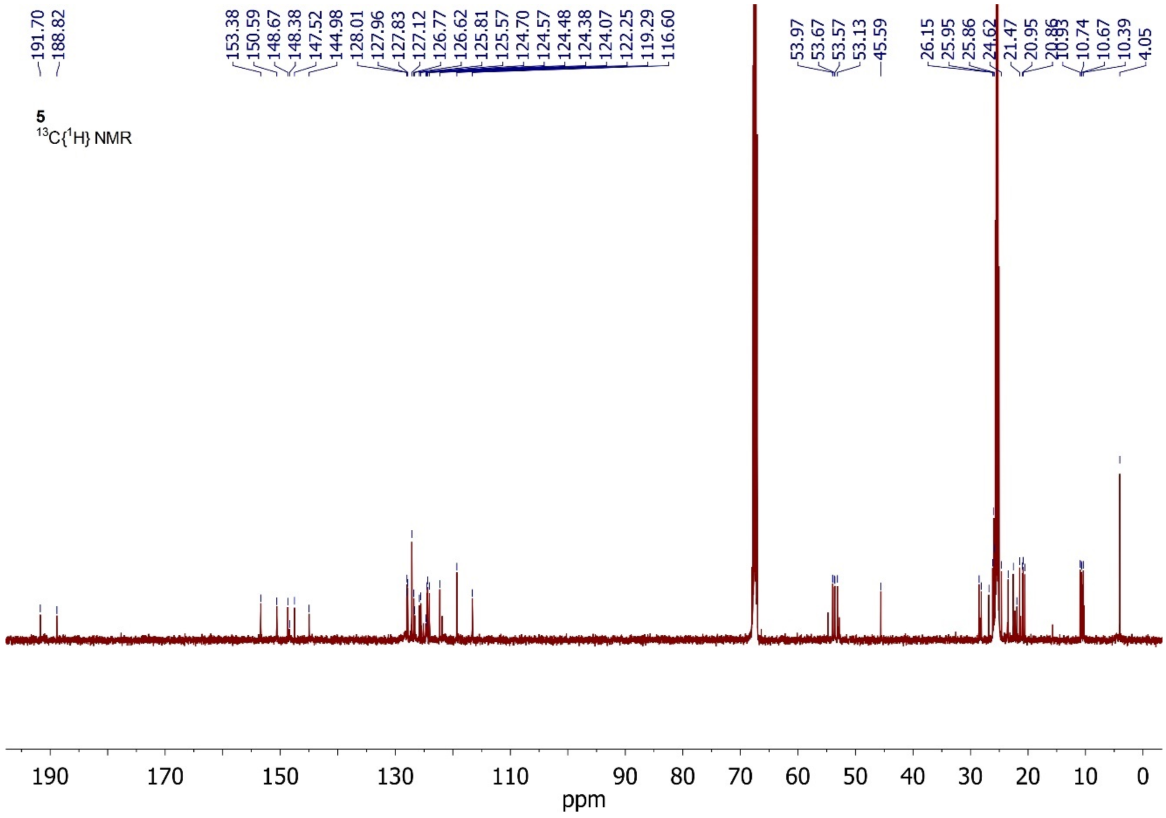

Figure S11. $^{13}C\{^1H\}$ NMR spectrum of **5** dissolved in D_8-THF, at 298K.

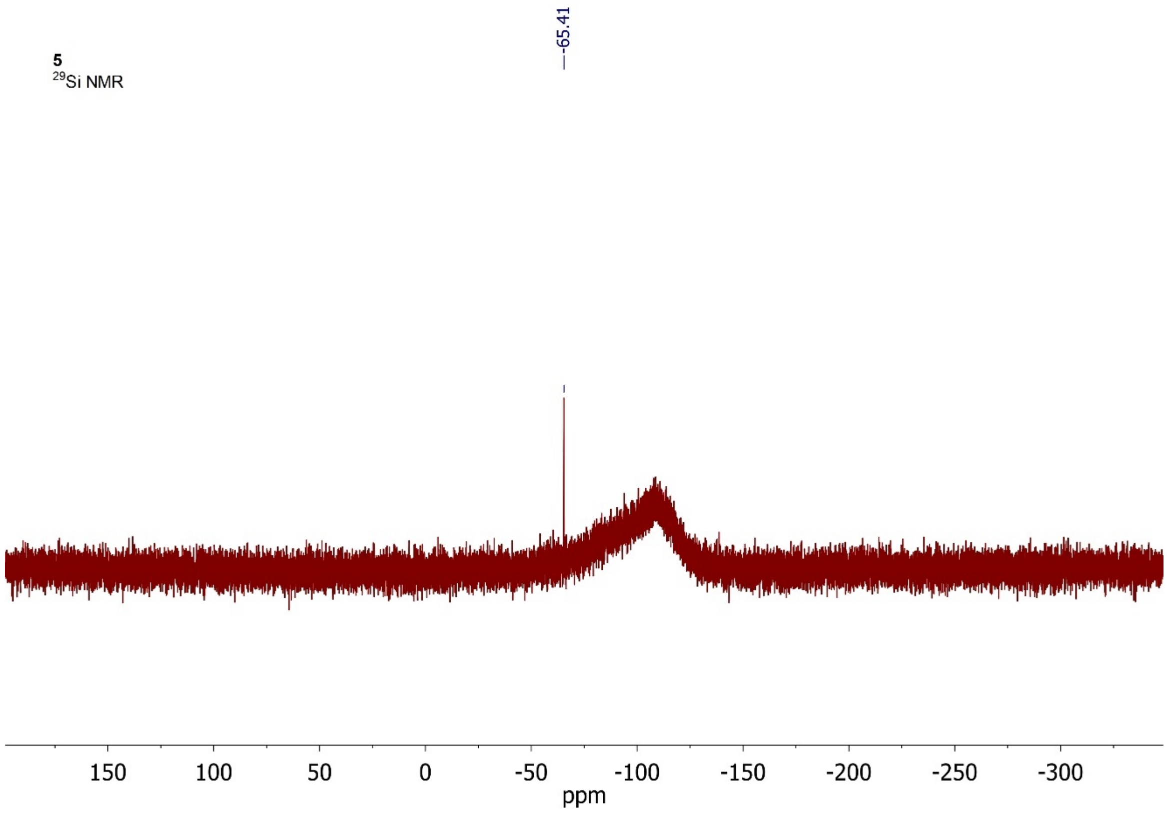

Figure S12. $^{29}Si\{^1H\}$ NMR spectrum of **5** dissolved in D_8-THF, at 298K.

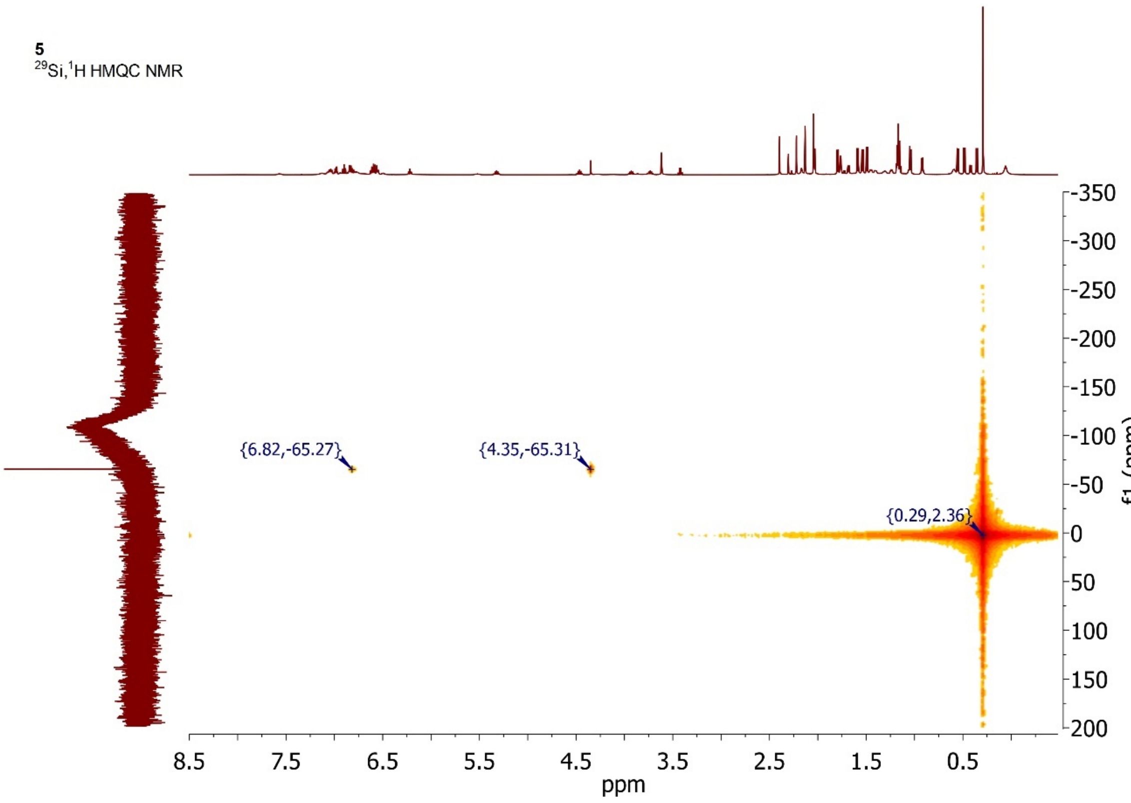

Figure S13. ^{29}Si,^{1}H HMQC NMR spectrum of **5** dissolved in D_8-THF, at 298K.

Synthesis of 6. A solution of **1** (0.15 g, 0.21 mmol) in toluene (10 mL) was cooled to -78 °C, and the atmosphere of the reaction vessel exchanged for acetylene. The color of the reaction mixture immediately became yellow, whereupon the atmosphere of the flask was exchanged for nitrogen gas through several vacuum cycles. If excess acetylene is not entirely removed from the reaction, insoluble dark purple material is formed upon warming the reaction mixture. The reaction mixture was subsequently warmed to ambient temperature, and all volatiles removed *in vacuo*. The yellow residue was extracted in warm hexane (15 mL), filtered, and concentrated to 5 mL. Storage of this solution at ambient temperature for one day resulted in the formation of a small crop of yellow crystals of **6** (90 mg, 59 %). ^{1}H NMR (C_6D_6, 400 MHz, 298 K): δ = 0.39 (s, 9H, Si*Me*$_3$), 0.74 (d, $^{3}J_{HH}$ = 7.2 Hz, 3H, NHC-Pr^{i}-C*H*$_3$), 0.85 (d, $^{3}J_{HH}$ = 7.2 Hz, 3H, NHC-Pr^{i}-C*H*$_3$), 1.18 (d, $^{3}J_{HH}$ = 7.2 Hz, 3H, NHC-Pr^{i}-C*H*$_3$), 1.12 (d, $^{3}J_{HH}$ = 7.2 Hz, 3H, NHC-Pr^{i}-C*H*$_3$), 1.25 (virt. t, $^{3}J_{HH}$ = 7.2 Hz, 6H, NHC-Pr^{i}-C*H*$_3$) 1.43 (d, $^{3}J_{HH}$ = 7.2 Hz, 3H, NHC-Pr^{i}-C*H*$_3$), 1.44 (d, $^{3}J_{HH}$ = 6.8 Hz, 3H, Dipp-Pr^{i}-C*H*$_3$), 1.46 (d, $^{3}J_{HH}$ = 6.8 Hz, 3H, Dipp-Pr^{i}-C*H*$_3$), 1.53 (d, $^{3}J_{HH}$ = 6.8 Hz, 3H, Dipp-Pr^{i}-C*H*$_3$), 1.62 (s, 3H, NHC-NC*Me*), 1.65 (d, $^{3}J_{HH}$ = 6.8 Hz, 3H, Dipp-Pr^{i}-C*H*$_3$), 1.70 (s, 3H, NHC-NC*Me*), 1.72 62 (s, 3H, NHC-NC*Me*), 1.76 (d, $^{3}J_{HH}$ = 7.2 Hz, 3H, NHC-Pr^{i}-C*H*$_3$), 1.80 (s, 3H, NHC-NC*Me*), 4.15 (sept, $^{3}J_{HH}$ = 6.8 Hz, 1H, Dipp-Pr^{i}-C*H*), 4.40 (sept, $^{3}J_{HH}$ = 6.8 Hz, 1H, Dipp-Pr^{i}-C*H*), 5.73 (sept, $^{3}J_{HH}$ = 7.2 Hz, 1H, NHC-Pr^{i}-C*H*), 5.90 (sept, $^{3}J_{HH}$ = 7.2 Hz, 1H, NHC-Pr^{i}-C*H*), 5.97 (sept, $^{3}J_{HH}$ = 7.2 Hz, 1H, NHC-Pr^{i}-C*H*), 6.61 (sept, $^{3}J_{HH}$ = 7.2 Hz, 1H, NHC-Pr^{i}-C*H*), 7.19 (m, 1H, *p*-Ar-C*H*), 7.27 (m, 2H, *m*-Ar-C*H*), 7.73 (d, $^{3}J_{HH}$ = 12.50 Hz, 1H, Si-C(*H*)=C(H)-Ni), 8.25 (d, $^{3}J_{HH}$ = 12.50 Hz, 1H, Si-C(H)=C(*H*)-Ni); ^{13}C{^{1}H} NMR (C_6D_6, 75.5 MHz, 298 K): δ = 3.4 (Si*Me*$_3$), 10.1, 10.2, 10.4 and 10.5 (NHC-NC*Me*), 21.1, 21.5, 21.6, 21.7, 22.2, 22.4, 23.2, and 24.3

(NHC-Pr^i-CH_3), 25.4, 25.8, 26.5, and 26.6 (Dipp-Pr^i-CH_3), 28.1 and 28.2 (Dipp-Pr^i-*C*H), 52.2, 52.5, 52.6, and 52.7 (NHC-Pr^i-*C*H), 124.2 and 124.3 (4 overlapping peaks, NHC-N*C*Me), 123.6, 124.8, 125.7, 144.4, 147.3, and 148.8 (Ar-*C*), 169.1 and 171.2 (Si-*C*(H)=*C*(H)-Ni), 190.8 and 194.6 (NHC-*C*:); ^{29}Si{^{1}H} NMR (C_6D_6, 80 MHz, 298 K): δ = 2.3 (*Si*Me_3), -59.8 (Si-C(H)=C(H)-Ni); anal. calcd. for $C_{39}H_{68}ClN_5NiSi_2$: C, 61.85 %; H, 9.05 %; N, 9.25 %; found: C, 61.45 %; H, 8.85 %; N, 9.07 %.

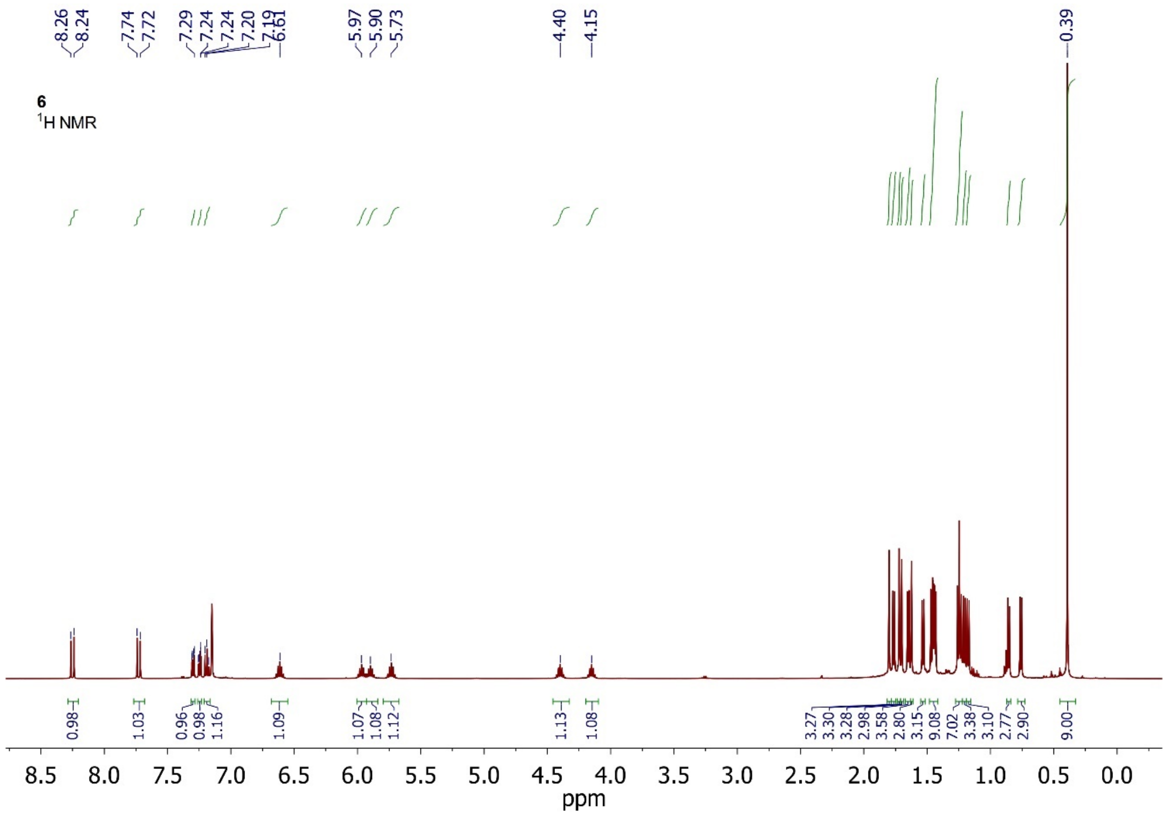

Figure S14. ^{1}H NMR spectrum of **6** dissolved in C_6D_6, at 298K.

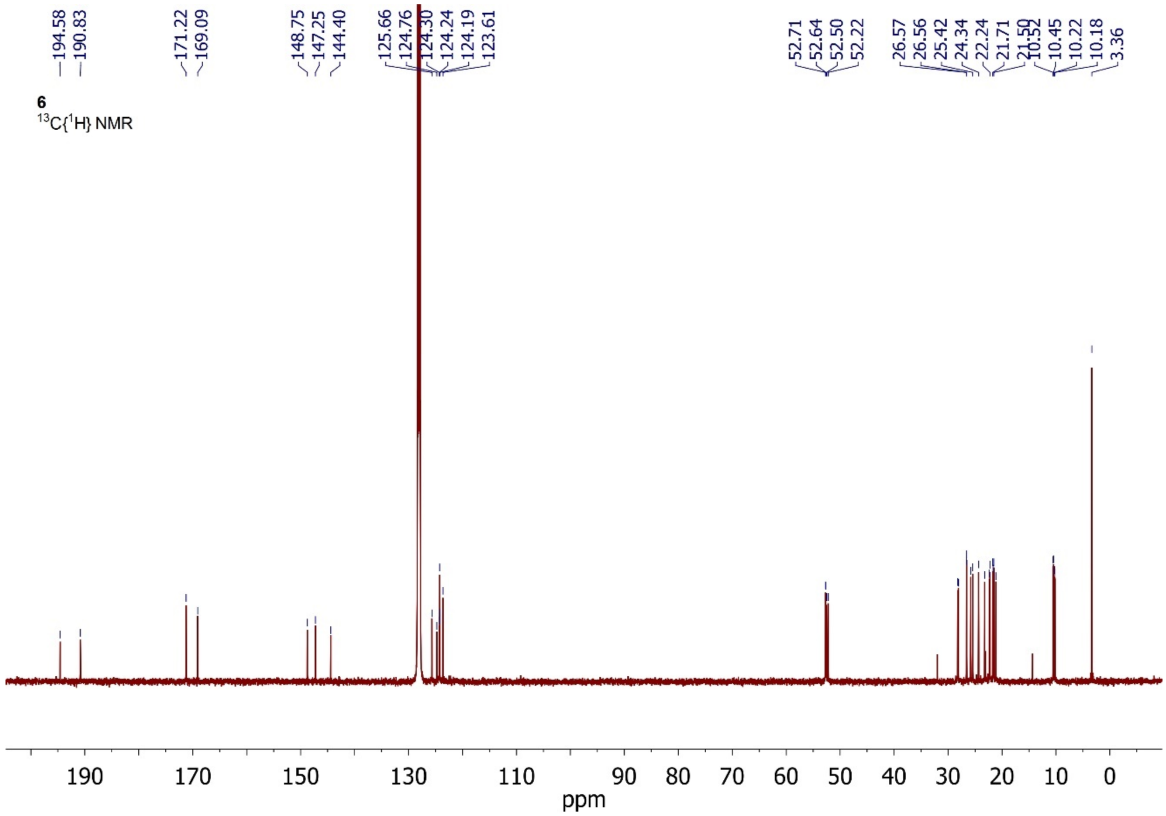

Figure S15. $^{13}C\{^1H\}$ NMR spectrum of **6** dissolved in C_6D_6, at 298K.

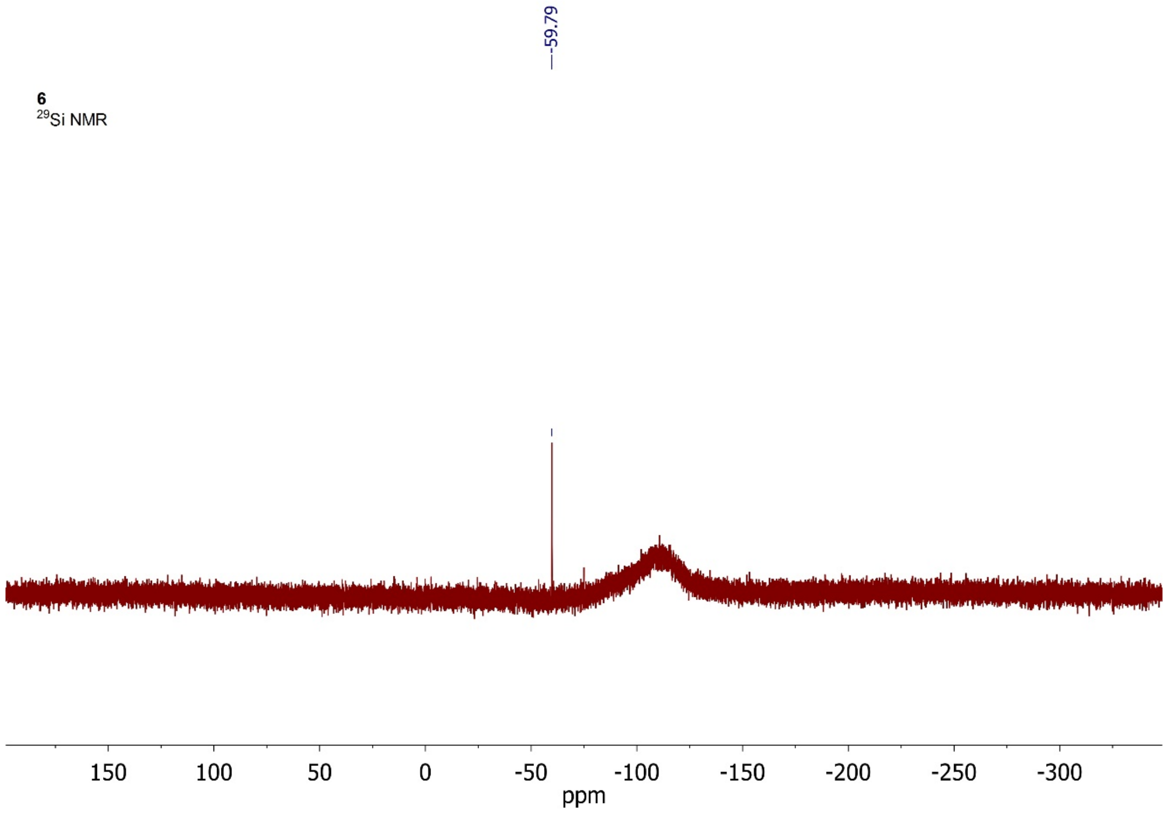

Figure S16. $^{29}Si\{^1H\}$ NMR spectrum of **6** dissolved in C_6D_6, at 298K.

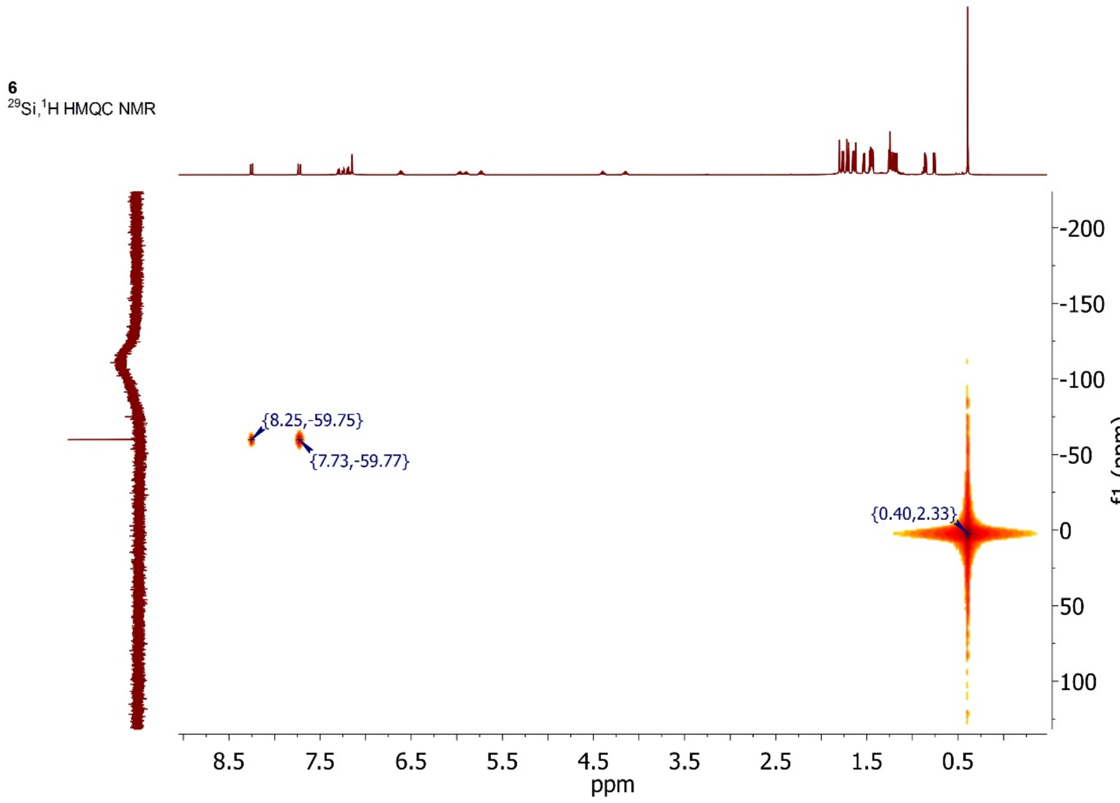

Figure S17. ^{29}Si,^{1}H HMQC NMR spectrum of **6** dissolved in C_6D_6, at 298K.

Synthesis of 6-OMe. To a solution of **1** (0.15 g, 0.21 mmol) in toluene (10 mL) was added 1,4-dimethoxy-2-butyne added *via* pipette (27 µL, 0.21 mmol) at ambient temperature. After stirring for 1 h, a color change to bright orange was observed. All volatiles were subsequently removed from the reaction mixture *in vacuo*, the solid residue extracted in hexane (10 mL), and filtered. Concentration of this solution to 5 mL and storage at 4 °C overnight resulted in the formation of large crop of orange crystals of **6-OMe** (85 mg, 49 %). ^{1}H NMR (C_6D_6, 400 MHz, 298 K): δ = 0.28 (s, 9H, Si*Me*$_3$), 0.76 (br dd, 6H, NHC-Pr^i-C*H*$_3$), 1.32 (br, 6H, NHC-Pr^i-C*H*$_3$), 1.38 (d, $^3J_{HH}$ = 7.2 Hz, 3H, NHC-Pr^i-C*H*$_3$), 1.41 (d, $^3J_{HH}$ = 6.8 Hz, 3H, Dipp-Pr^i-C*H*$_3$), 1.43 (d, $^3J_{HH}$ = 6.8 Hz, 3H, Dipp-Pr^i-C*H*$_3$), 1.46 (d, $^3J_{HH}$ = 6.8 Hz, 3H, Dipp-Pr^i-C*H*$_3$), 1.50 (br, 3H, NHC-Pr^i-C*H*$_3$), 1.59 (br, 3H, NHC-Pr^i-C*H*$_3$), 1.62 (d, $^3J_{HH}$ = 7.2 Hz, 3H, NHC-Pr^i-C*H*$_3$), 1.68 (s, 3H, NHC-NC*Me*), 1.69 (d, $^3J_{HH}$ = 6.8 Hz, 3H, Dipp-Pr^i-C*H*$_3$), 1.73 (s, 6H, NHC-NC*Me*), 1.80 (s, 3H, NHC-NC*Me*), 3.12 (s, 3H, CH$_2$-O*Me*), 3.41 (s, 3H, CH$_2$-O*Me*), 3.81 (m, 1H, C*H*$_2$-OMe), 3.94 (m, 1H, C*H*$_2$-OMe), 3.96 (sept, $^3J_{HH}$ = 6.8 Hz, 2H, Dipp-Pr^i-C*H*), 4.51 (sept, $^3J_{HH}$ = 6.8 Hz, 2H, Dipp-Pr^i-C*H*),5.37 (br, 1H, NHC-Pr^i-C*H*), 5.94 (br, 1H, NHC-Pr^i-C*H*), 6.49 (br, 1H, NHC-Pr^i-C*H*), 6.93 (br, 1H, NHC-Pr^i-C*H*), 7.20 (m, 2H, *m*-Ar-C*H*), 7.29 (m, 1H, *p*-Ar-C*H*); ^{13}C{^{1}H} NMR (C_6D_6, 75.5 MHz, 298 K): δ = 2.6 (Si*Me*$_3$), 9.8, 9.9, 10.1 (4 signals, NHC-NC*Me*), 20.8, 21.0, 21.3, 21.5, 21.7, 21.9, 22.5, and 22.6 (NHC-Pr^i-*C*H$_3$), 25.2, 25.6, 25.7, and 26.6 (Dipp-Pr^i-*C*H$_3$), 27.7 and 27.8 (Dipp-Pr^i-*C*H), 51.6, 52.0, 52.3, and 52.6 (NHC-Pr^i-*C*H), 57.1 and 57.4 (CH$_2$O*Me*), 69.8 and 77.6 (*C*H$_2$OMe), 123.0 and 125.0 (NHC-N*C*Me), 123.6, 124.1, 124.4, 124.6, 142.5, 145.0, 147.3, and 147.8 (Ar-*C*), 182.7 and 191.7 (MeOCH$_2$*C*=*C*CH$_2$OMe), 192.7 (NHC-*C*:); ^{29}Si{^{1}H} NMR (C_6D_6, 80 MHz, 298 K): δ = 2.2 (*Si*Me$_3$), -63.3 (*Si*-C=C-Ni); anal. calcd. for $C_{43}H_{76}ClN_5NiO_2Si_2$: C, 61.09 %; H, 9.06 %; N, 8.28 %; found: C, 61.01 %; H, 8.14%; N, 8.91 %.

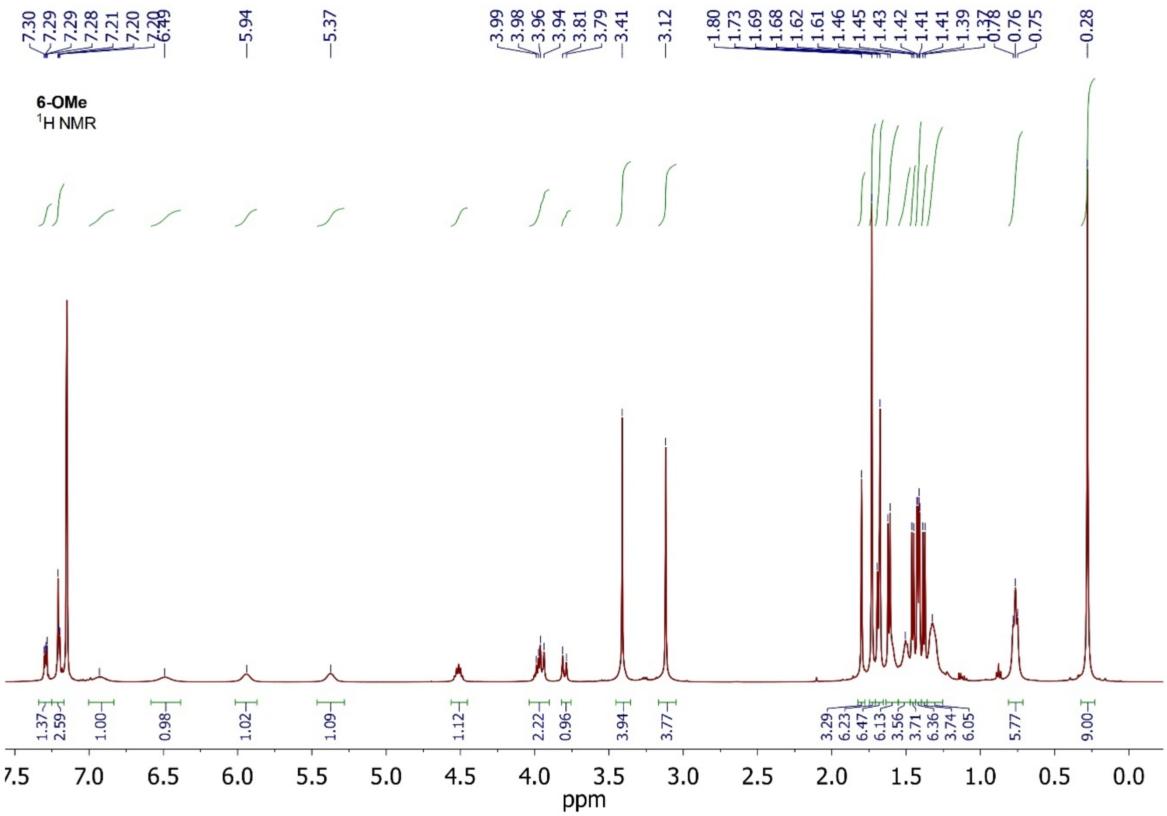

Figure S18. ^{1}H NMR spectrum of **6-OMe** dissolved in C_6D_6, at 298K.

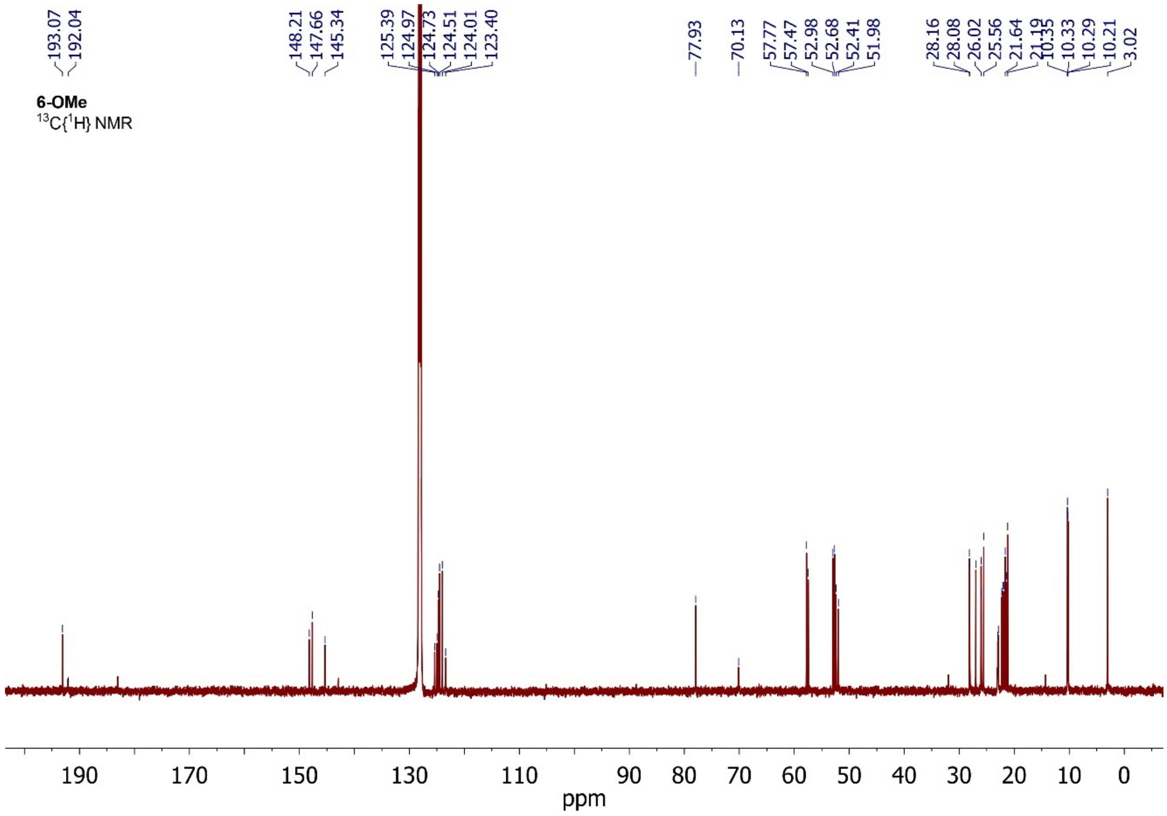

Figure S19. ^{13}C{^{1}H} NMR spectrum of **6-OMe** dissolved in C_6D_6, at 298K.

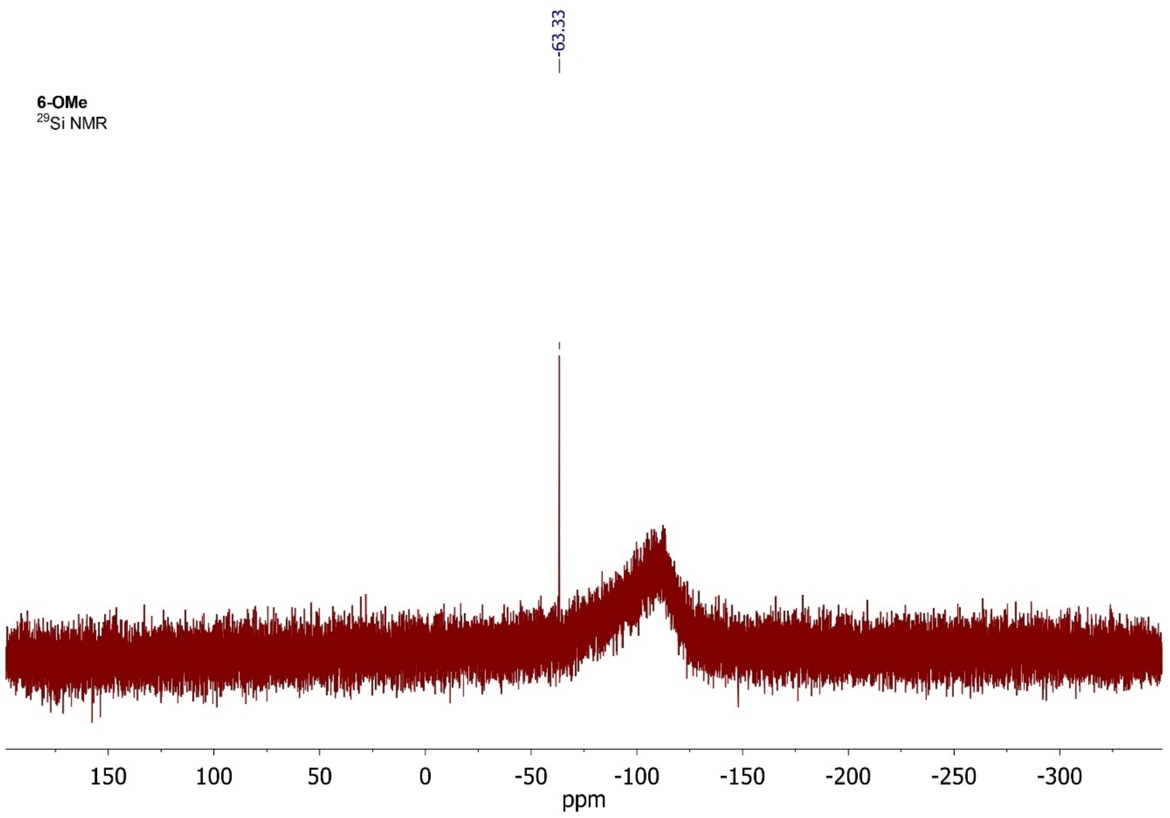

Figure S20. $^{29}Si\{^1H\}$ NMR spectrum of **6-OMe** dissolved in C_6D_6, at 298K.

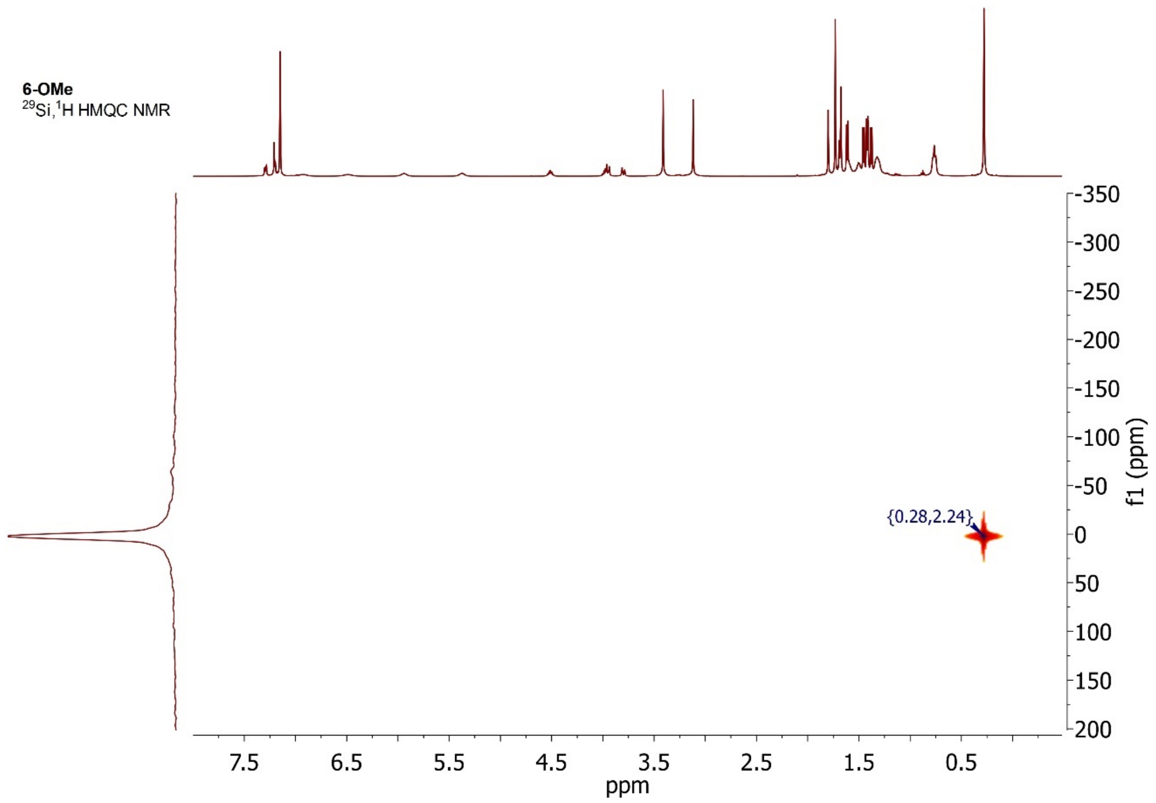

Figure S21. $^{29}Si,^1H$ HMQC NMR spectrum of **6-OMe** dissolved in C_6D_6, at 298K.

Synthesis of 7. A solution of **1** (0.15 g, 0.21 mmol) in diethyl ether (25 mL) was cooled to -78 °C, and treated with an atmosphere of ethylene. The color of the reaction mixture immediately became yellow, whereupon the atmosphere of the flask was exchanged for nitrogen gas through several vacuum cycles. The solution was warmed to 0 °C, filtered into a precooled Schlenk flask, and concentrated to ~10 mL by blowing a stream of nitrogen gas over the solution. The reaction mixture was stored at -30 °C overnight to yield a small crop of yellow plate-like crystals (45 mg, 29 %). ^{1}H NMR (D_8-THF, 400 MHz, 298 K): δ = -0.06 (s, 9H, Si*Me*$_3$), 0.57 (m, 2H, Ni-CH_2C*H*$_2$-Si), 0.80 (d, $^{3}J_{HH}$ = 6.8 Hz, 3H, Dipp-Pr^{i}-C*H*$_3$), 0.95 (d, $^{3}J_{HH}$ = 6.8 Hz, 3H, Dipp-Pr^{i}-C*H*$_3$), 1.06 (d, $^{3}J_{HH}$ = 6.8 Hz, 3H, Dipp-Pr^{i}-C*H*$_3$), 1.19 (overlapping d, 6H, NHC-iPr-C*H*$_3$), 1.34 (d, $^{3}J_{HH}$ = 6.8 Hz, 3H, Dipp-Pr^{i}-C*H*$_3$), 1.34 (m, 2H, Ni-C*H*$_2$$CH_2$-Si) 1.38 (d, $^{3}J_{HH}$ = 6.8 Hz, 3H, Dipp-Pr^{i}-C*H*$_3$), 1.50 (d, $^{3}J_{HH}$ = 7.2 Hz, 6H, NHC-Pr^{i}-C*H*$_3$), 1.53 (d, $^{3}J_{HH}$ = 7.2 Hz, 6H, NHC-Pr^{i}-C*H*$_3$), 1.66 (d, $^{3}J_{HH}$ = 7.2 Hz, 3H, Dipp-Pr^{i}-C*H*$_3$), 2.10 (s, 6H, NHC-NC*Me*), 2.19 (s, 6H, NHC-NC*Me*), 2.21 (s, 6H, NHC-NC*Me*), 3.79 (sept, $^{3}J_{HH}$ = 6.8 Hz, 1H, Dipp-Pr^{i}-C*H*), 3.91 (sept, $^{3}J_{HH}$ = 6.8 Hz, 2H, Dipp-Pr^{i}-C*H*), 5.50 (sept, $^{3}J_{HH}$ = 7.2 Hz, 1H, NHC-Pr^{i}-C*H*), 5.57 (sept, $^{3}J_{HH}$ = 7.2 Hz, 1H, NHC-Pr^{i}-C*H*), 6.31 (sept, $^{3}J_{HH}$ = 7.2 Hz, 1H, NHC-Pr^{i}-C*H*), 6.78 (sept, $^{3}J_{HH}$ = 7.2 Hz, 1H, NHC-Pr^{i}-C*H*), 6.87 (m, 1H, *p*-Ar-C*H*), 6.94 (m, 2H, *m*-Ar-C*H*); ^{13}C{^{1}H} NMR (D_8-THF, 75.5 MHz, 233 K): δ = 1.7 (Ni-$CH_2$$CH_2$-Si), 3.2 (Si*Me*$_3$), 10.3, 10.4, 10.6, and 10.8 (NHC-NC*Me*), 21.0 (Ni-*C*$H_2$$CH_2$-Si), 21.2, 21.7, 21.9, 22.0, 22.9, 24.3, 25.9, and 26.0 (NHC-Pr^{i}-*C*H_3), 26.2, 26.8, 28.0, and 28.5 (Dipp-Pr^{i}-*C*H_3), 30.8 (br, Dipp-Pr^{i}-*C*H), 53.0, 53.2, 53.3, and 53.4 (NHC-Pr^{i}-*C*H), 123.5, 124.0, 124.7, and 126.7 (NHC-N*C*Me), 123.9, 124.7, 126.7, 144.6, 147.0, and 148.3 (Ar-*C*), 191.6, and 194.3 (NHC-*C*:); ^{29}Si{^{1}H} NMR (D_8-THF, 80 MHz, 298 K): δ = 1.8 (*Si*Me$_3$), -26.0 (Ni-C_2H_4-*Si*); anal. calcd. for $C_{39}H_{70}ClN_5NiSi_2$: C, 61.69 %; H, 9.29 %; N, 9.22 %; found: C, 62.01 %; H, 9.25%; N, 9.44 %.

N.B. Keeping an NMR sample of **7** at ambient temperature for 24 h results in complete disproportionation to **1** and **9**. However, a few crystals of **8** could be isolated when reacting a hexane solution of **1** with excess ethylene followed by storage at low-temperature for several days, from which a poor-quality structure could be obtained (see below), confirming its connectivity. No further data was obtained for this compound, however.

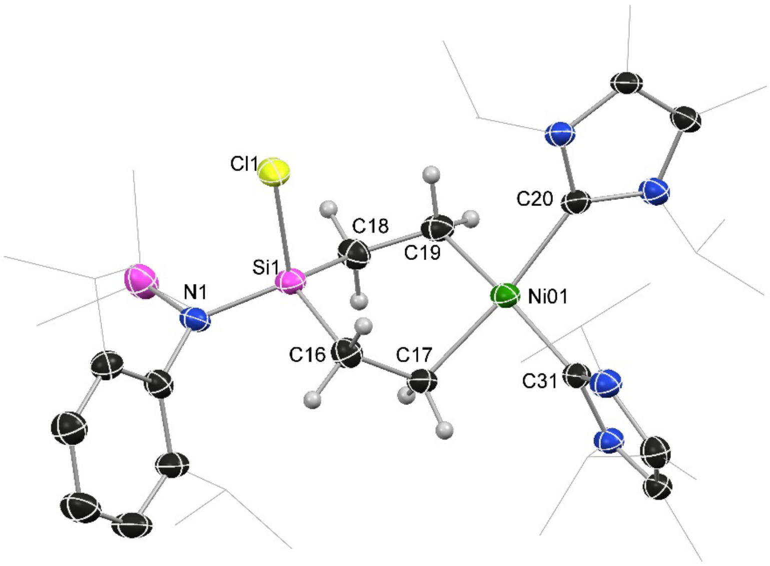

Figure S22. Molecular structure of **8**, with thermal ellipsoids at 30% probability.

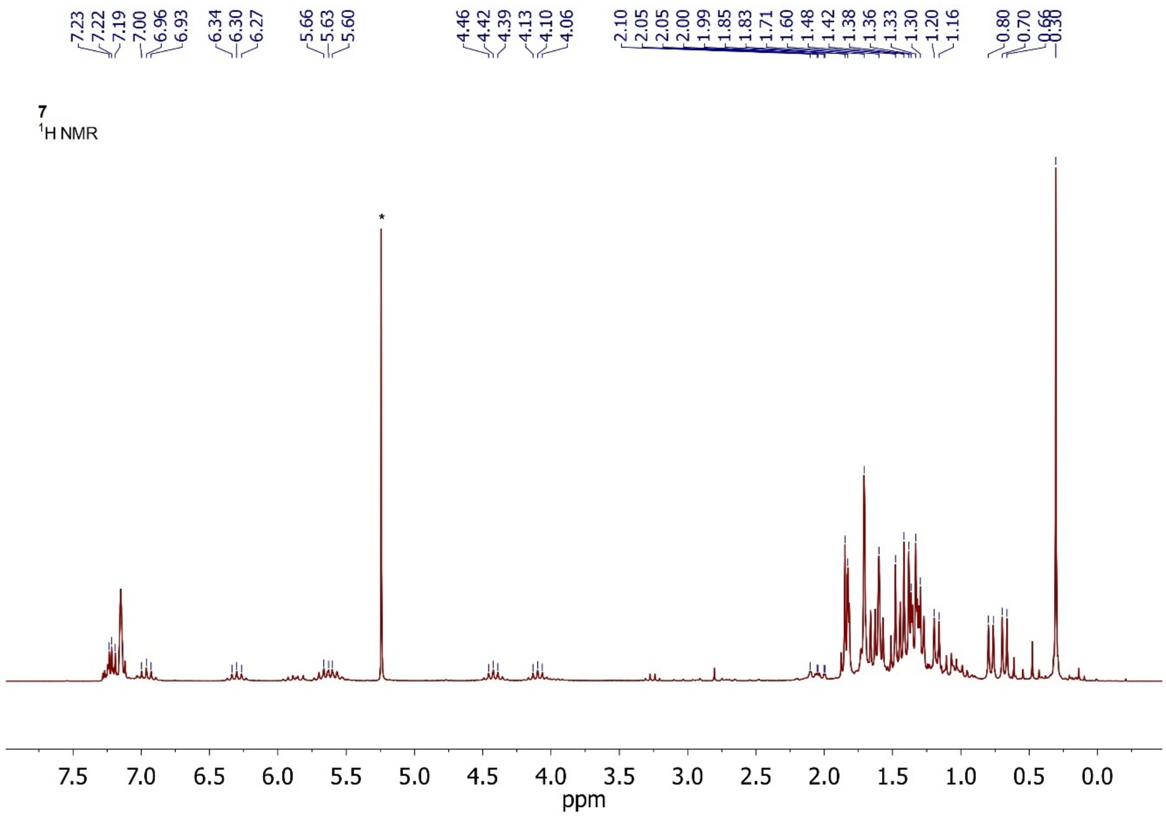

Figure S23. ^{1}H NMR spectrum of **7** dissolved in C_6D_6, at 298K. An asterisk indicates excess ethylene, required to prevent regeneration of starting material **1**.

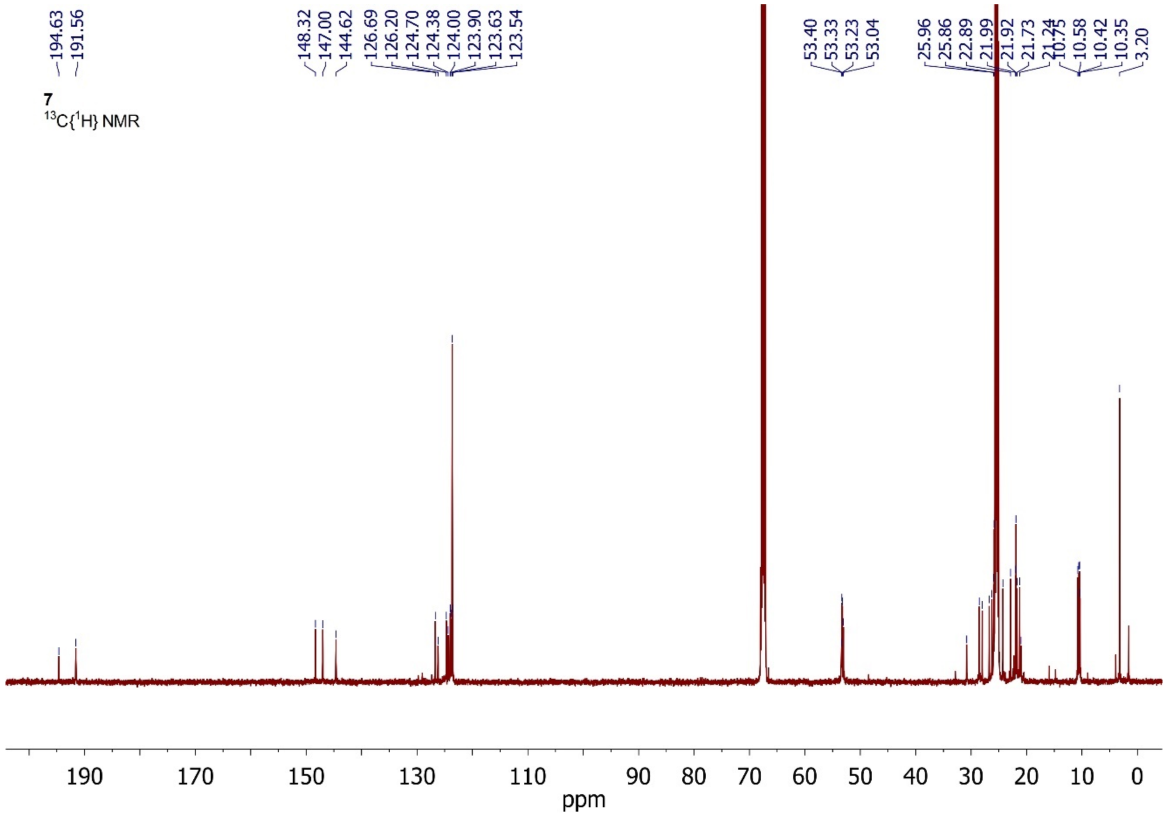

Figure S24. $^{13}C\{^{1}H\}$ NMR spectrum of **7** dissolved in C_6D_6, at 298K.

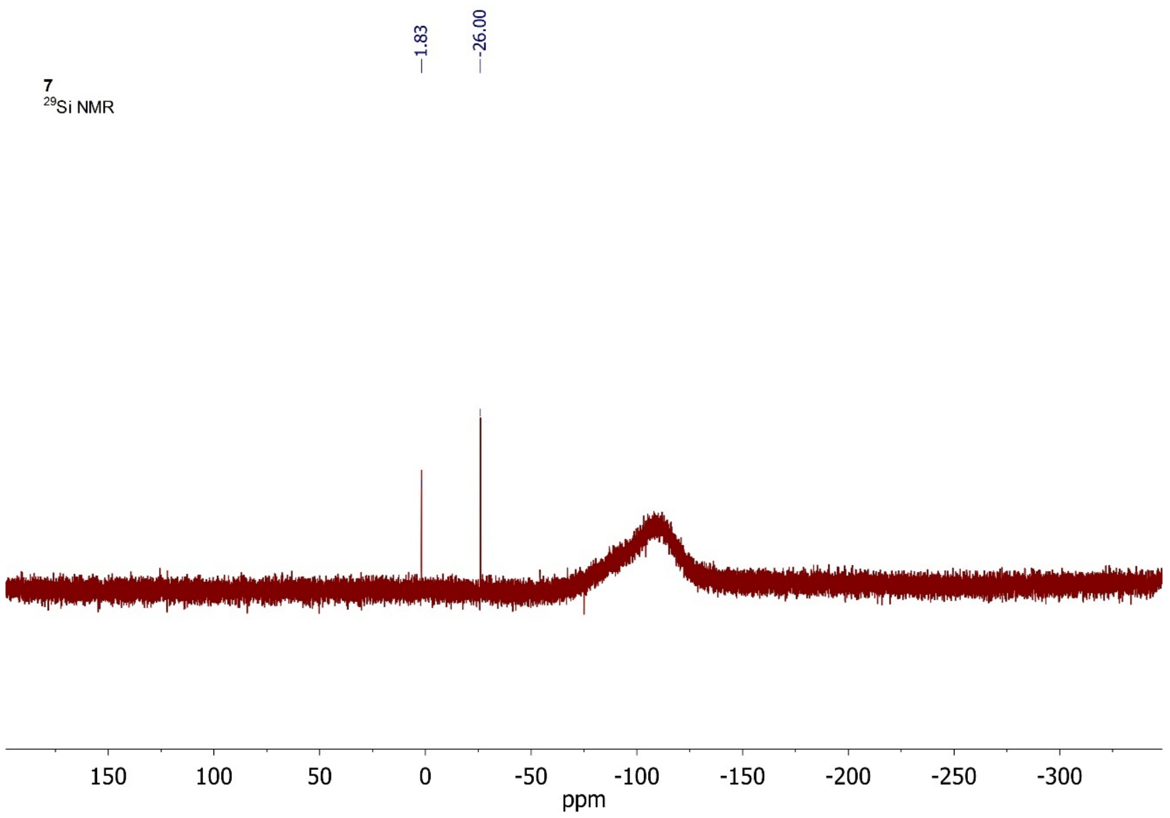

Figure S25. $^{29}Si\{^{1}H\}$ NMR spectrum of **7** dissolved in C_6D_6, at 298K.

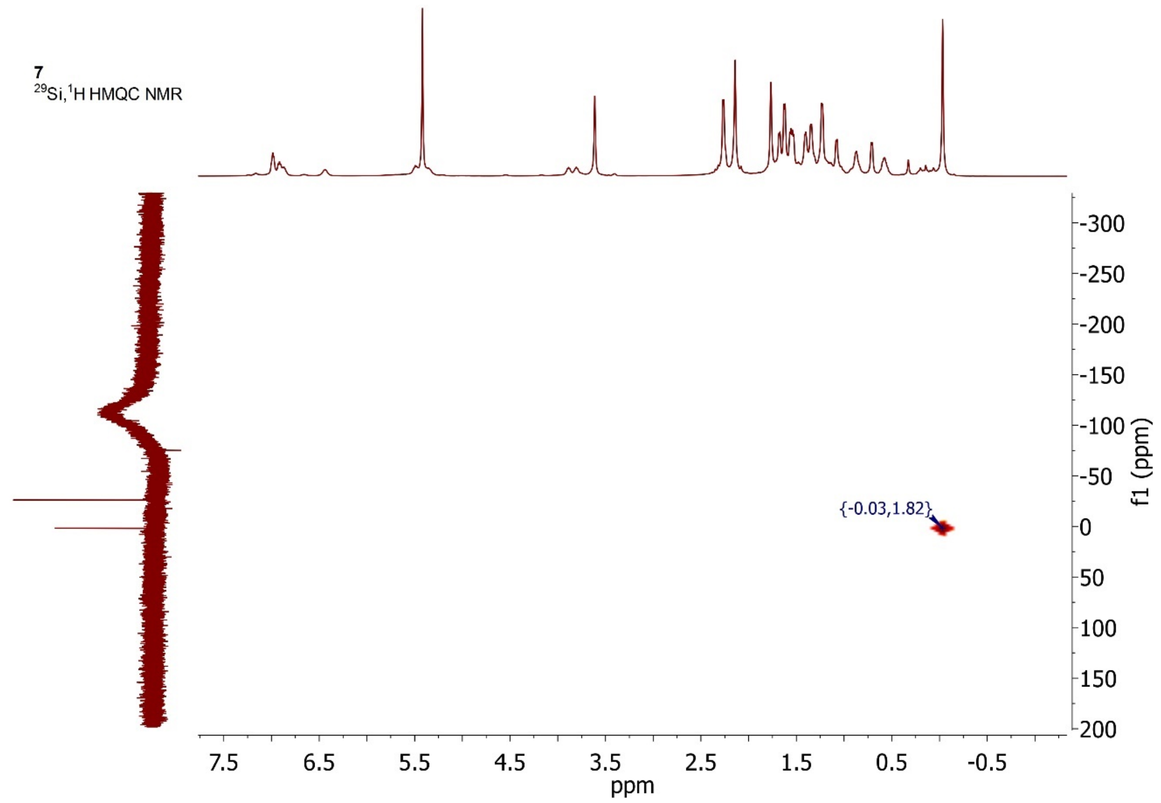

Figure S26. $^{29}Si,^{1}H$ HMQC NMR spectrum of **7** dissolved in C_6D_6, at 298K.

Synthesis of 9. A solution of **1** (0.15 g, 0.21 mmol) in toluene (10 mL) was stirred under an atmosphere of ethylene for 18 h at ambient temperature, resulting in a pale yellow solution. All volatiles were subsequently removed from the reaction mixture *in vacuo*, the residue extracted in hexane (10 mL), and filtered. Concentration of the filtrate to ~5 mL and storage at ambient temperature overnight resulted in the formation of a large crop of yellow crystalline material. (105 mg, 65 %). ^{1}H NMR (C_6D_6, 400 MHz, 298 K): δ = 0.50 (m, 1H, Si-CH_2CH$_3$), 0.57 (m, 3H, Si-CH$_2$CH_3), 0.65 (s, 9H, SiMe_3), 0.70 (m, 1H, Si-CH_2CH$_3$), 0.81 (d, $^{3}J_{HH}$ = 6.8 Hz, 3H, NHC-Pr^{i}-CH_3), 1.01 (d, $^{3}J_{HH}$ = 6.8 Hz, 3H, NHC-Pr^{i}-CH_3), 1.12 (m, 1H, Ni-(H$_2$C=C(*H*)Si)), 1.23 (d, $^{3}J_{HH}$ = 6.8 Hz, 3H, NHC-Pr^{i}-CH_3), 1.25 (d, $^{3}J_{HH}$ = 6.8 Hz, 3H, NHC-Pr^{i}-CH_3), 1.38 (d, $^{3}J_{HH}$ = 6.8 Hz, 6H, Dipp-Pr^{i}-CH_3), 1.40 (d, $^{3}J_{HH}$ = 7.2 Hz, 6H, NHC-Pr^{i}-CH_3), 1.44 (d, $^{3}J_{HH}$ = 7.2 Hz, 3H, NHC-Pr^{i}-CH_3), 1.45 (d, $^{3}J_{HH}$ = 7.2 Hz, 3H, NHC-Pr^{i}-CH_3), 1.55 (d, $^{3}J_{HH}$ = 6.8 Hz, 6H, Dipp-Pr^{i}-CH_3), 1.68 (s, 3H, NHC-NC*Me*), 1.74 (s, 3H, NHC-NC*Me*), 1.75 (s, 3H, NHC-NC*Me*), 1.87 (s, 3H, NHC-NC*Me*), 1.95 (m, 1H, Ni-(H_2C=C(H)Si), 2.04 (m, 1H, Ni-(H_2C=C(H)Si), 4.05 (sept, $^{3}J_{HH}$ = 6.8 Hz, 1H, Dipp-Pr^{i}-C*H*), 4.25 (sept, $^{3}J_{HH}$ = 6.8 Hz, 1H, Dipp-Pr^{i}-C*H*), 5.52 (sept, $^{3}J_{HH}$ = 7.2 Hz, 1H, NHC-Pr^{i}-C*H*), 5.74 (sept, $^{3}J_{HH}$ = 7.2 Hz, 1H, NHC-Pr^{i}-C*H*), 6.00 (sept, $^{3}J_{HH}$ = 7.2 Hz, 1H, NHC-Pr^{i}-C*H*), 6.40 (sept, $^{3}J_{HH}$ = 7.2 Hz, 1H, NHC-Pr^{i}-C*H*), 7.11 (m, 3H, Ar-C*H*); ^{13}C{^{1}H} NMR (C_6D_6, 75.5 MHz, 298 K): δ = 3.7 (Si*Me*$_3$), 8.9 (Si-CH$_2$CH_3), 10.2, 10.3, and 10.4 (NHC-NC*Me*), 12.2 (Si-CH$_2$CH$_3$), 21.6, 21.7, 21.9, 22.1, 22.7, 23.4, 24.9, and 25.3 (NHC-Pr^{i}-CH$_3$), 25.1 (Ni-(H$_2$C=*C*(H)Si), 26.5 and 26.6 (Dipp-Pr^{i}-*C*H$_3$), 27.9 (Ni-(H$_2$*C*=C(H)Si), 30.1 (Dipp-Pr^{i}-*C*H), 51.6, 52.1, and 52.5 (NHC-Pr^{i}-*C*H), 122.9, 123.1, 123.5, and 123.7 (NHC-N*C*Me), 123.7, 124.6, 124.9, 144.2, 146.8, and 148.2 (Ar-*C*), 202.1 and 202.3 (NHC-*C*:); ^{29}Si{^{1}H} NMR (C_6D_6, 80 MHz, 298 K): δ = 5.2 (*Si*Me$_3$), 7.8 (^{TMS}L(Cl)*Si*Et); anal. calcd. for $C_{41}H_{74}ClN_5NiSi_2$: C, 62.54 %; H, 9.47 %; N, 8.89 %; found: C, 62.18 %; H, 9.29%; N, 8.66 %.

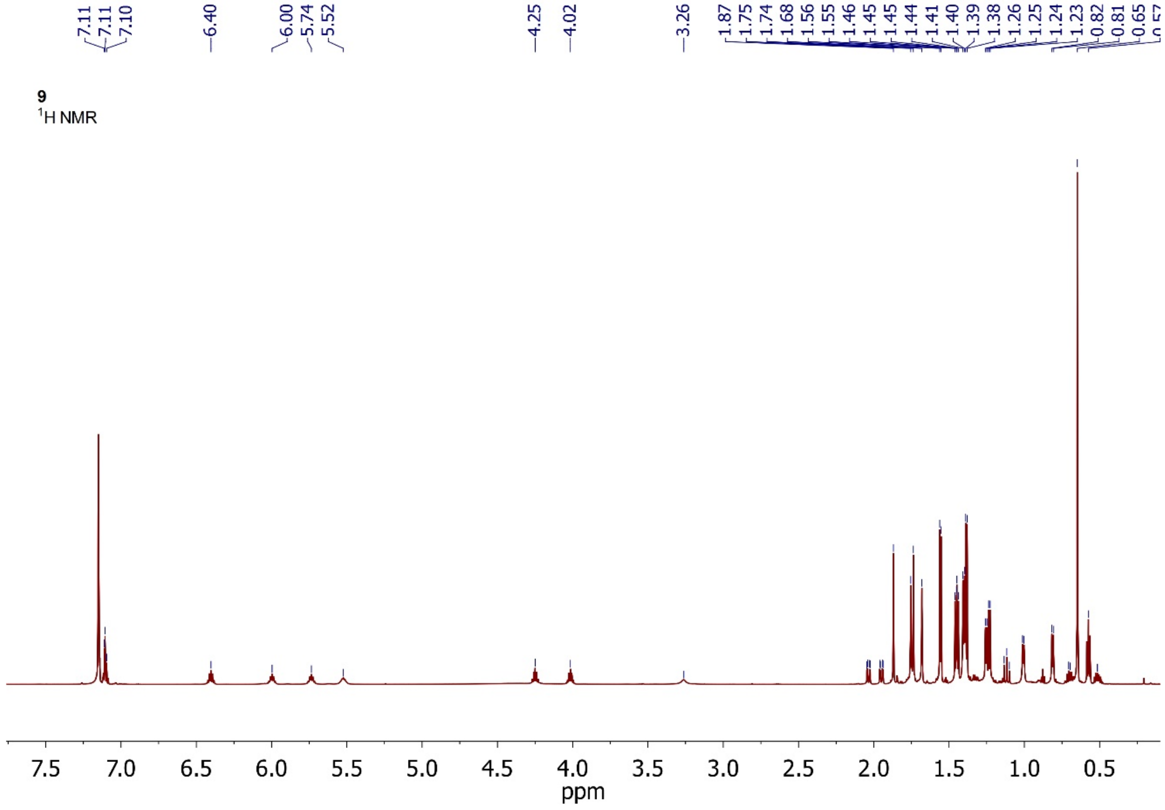

Figure S27. ^{1}H NMR spectrum of **9** dissolved in C_6D_6, at 298K.

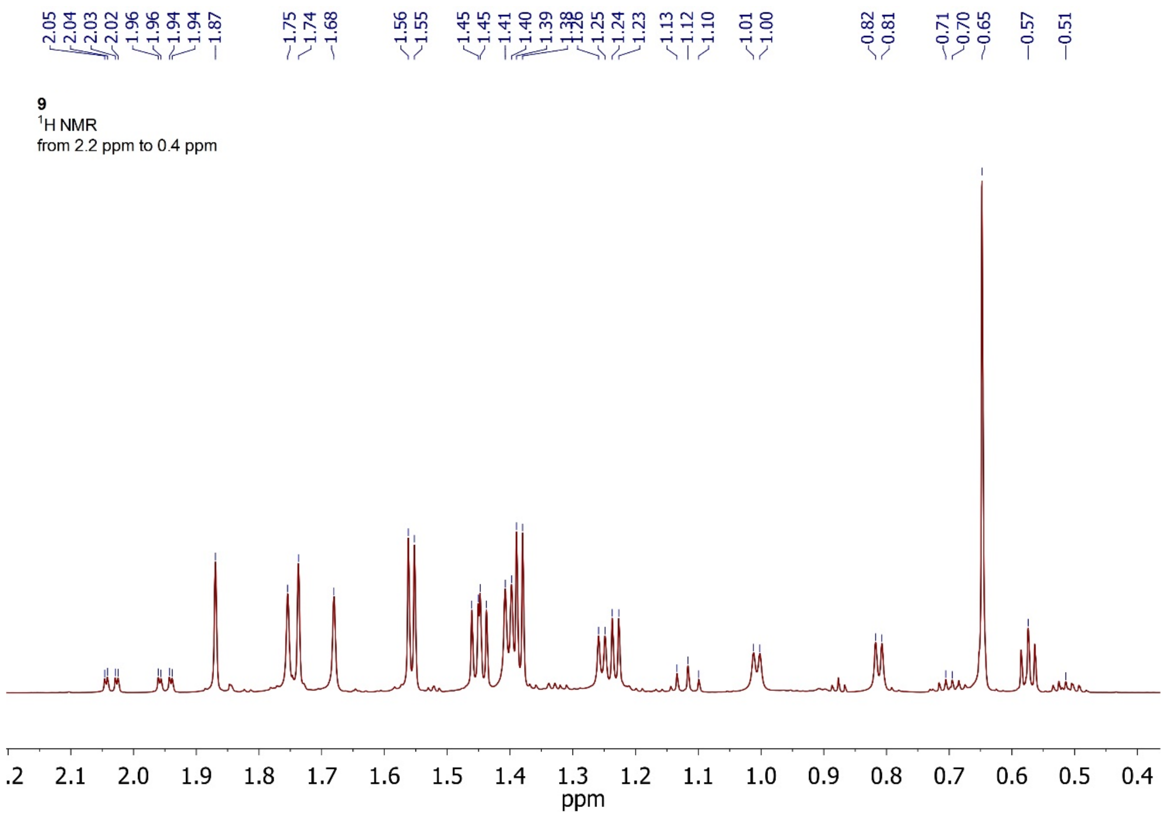

Figure S28. Zoom of the ^{1}H NMR spectrum of **9** dissolved in C_6D_6, from 0.4 ppm to 2.2 ppm, at 298K.

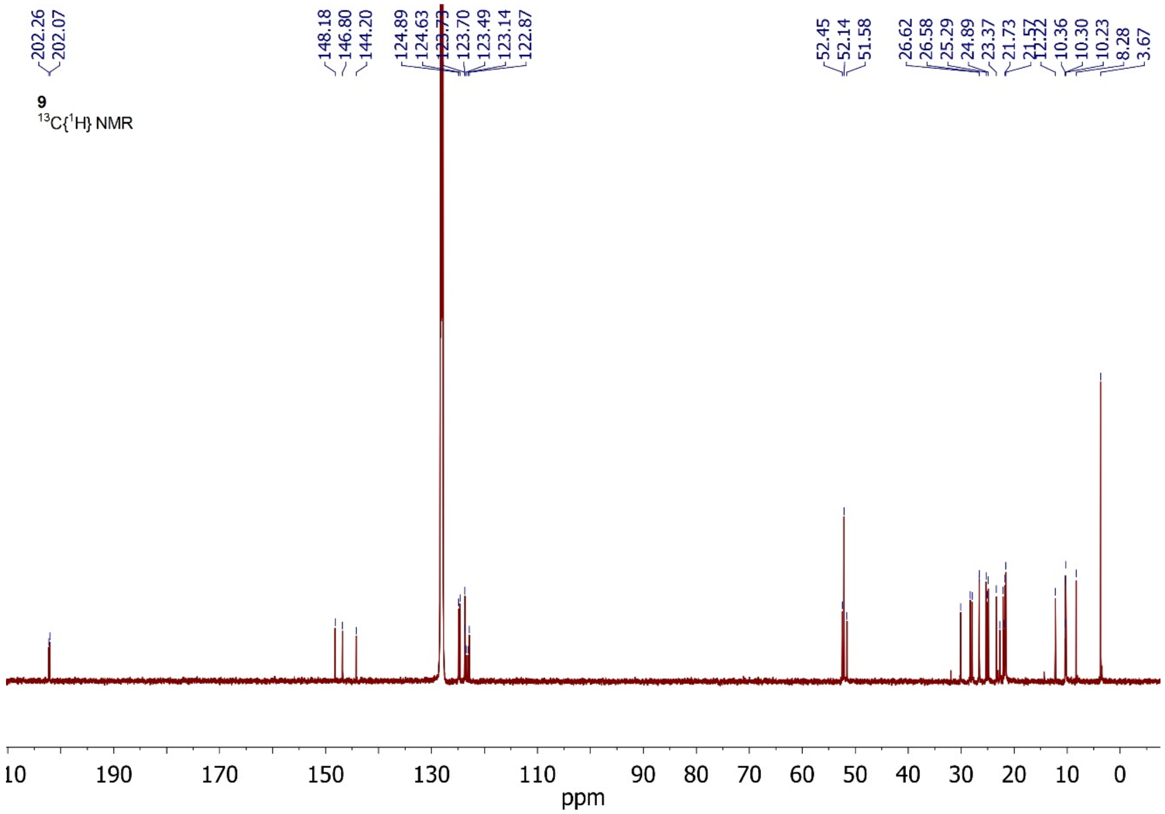

Figure S29. $^{13}C\{^1H\}$ NMR spectrum of **9** dissolved in C_6D_6, at 298K.

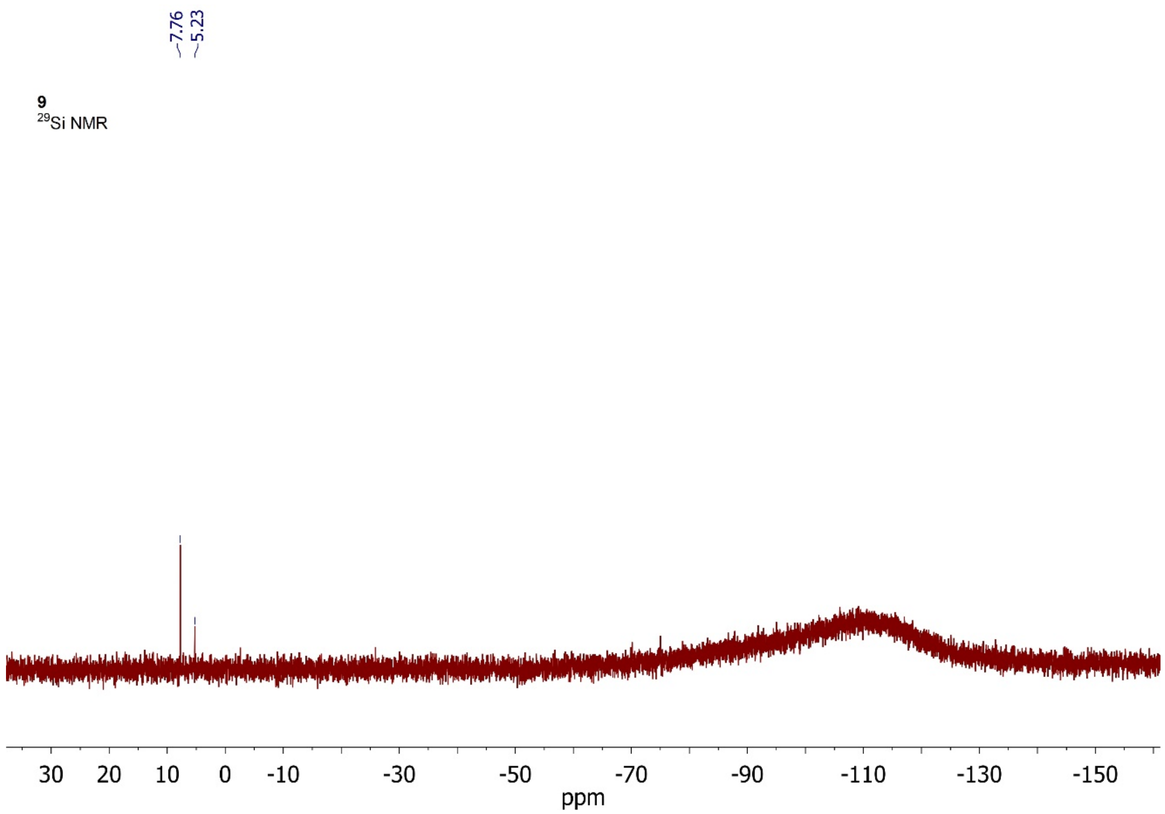

Figure S30. $^{29}Si\{^1H\}$ NMR spectrum of **9** dissolved in C_6D_6, at 298K.

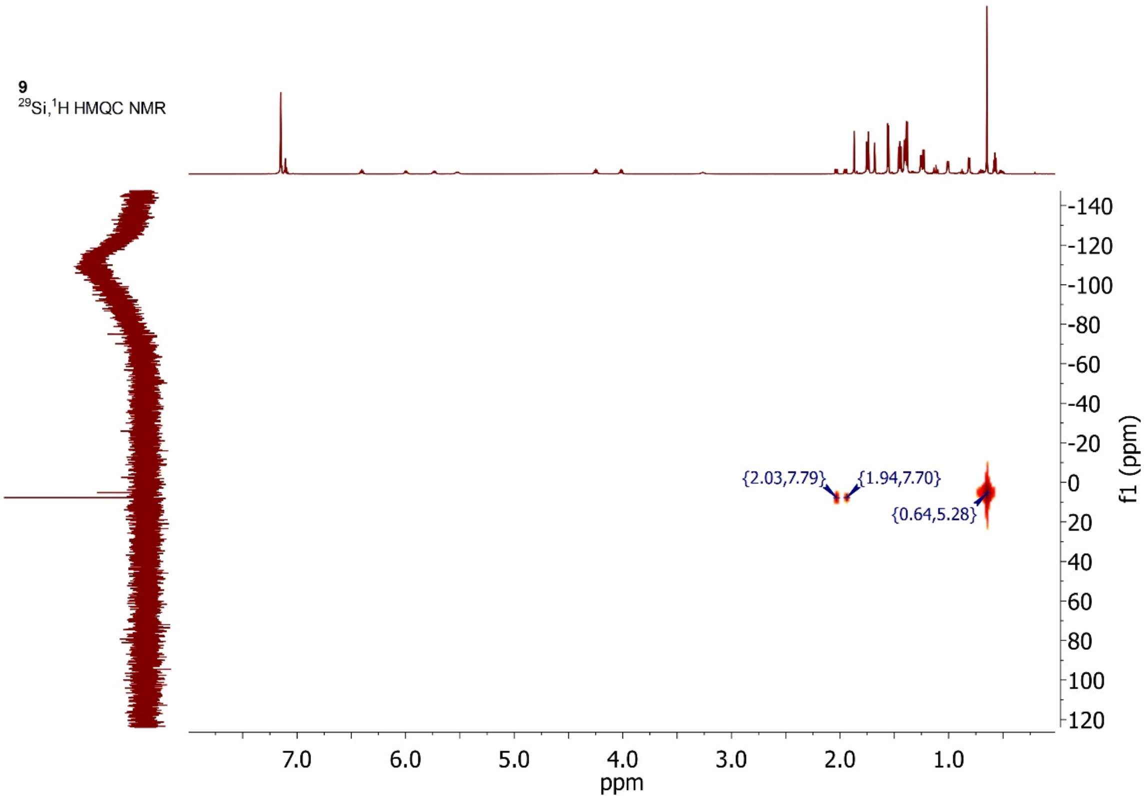

Figure S31. $^{29}Si,^1H$ HMQC NMR spectrum of **9** dissolved in C_6D_6, at 298K.

Synthesis of 9-Ph. To a solution of **1** (0.15 g, 0.21 mmol) in toluene (10 mL) was added styrene (52 μL, 0,45 mmol) at -78 °C. The reaction was warmed to ambient temperature and stirred for 18 h, resulting in a yellow-brown solution. All volatiles were removed from this mixture *in vacuo*, and the residue extracted in hexane (10 mL). Filtration, concentration to 4 mL, and storage at ambient temperature resulted in the formation of a large crop of yellow crystalline material (100 mg, 52 %). ^{1}H NMR (C_6D_6, 400 MHz, 298 K): δ = 0.41 (s, 9H, Si*Me*$_3$), 1.09 (br, 18H, NHC-Pr^i-C*H*$_3$), 1.35 (overlapping d, 9H, Dipp-Pr^i-C*H*$_3$), 1.45 (d, $^3J_{HH}$ = 6.8 Hz, 3H, Dipp-Pr^i-C*H*$_3$), 1.53 (br, 6H, NHC-Pr^i-C*H*$_3$) 1.76 (s, 6H, NHC-NC*Me*), 1.81 (s, 6H, NHC-NC*Me*), 3.98 (sept, $^3J_{HH}$ = 6.8 Hz, 1H, Dipp-Pr^i-C*H*), 4.09 (sept, $^3J_{HH}$ = 6.8 Hz, 1H, Dipp-Pr^i-C*H*), 5.81 (br, 2H, NHC-Pr^i-C*H*), 5.85 (sept, $^3J_{HH}$ = 7.2 Hz, 2H, NHC-Pr^i-C*H*), 6.89 (m, 1H, Ar-C*H*), 7.06 (m, 4H, Ar-C*H*), 7.21 (m, 3H, Ar-C*H*); ^{13}C{^{1}H} NMR (C_6D_6, 75.5 MHz, 298 K): δ = 2.6 (Si*Me*$_3$), 10.4 and 10.5 (NHC-NC*Me*), 21.9, 22.7, and 23.1 (br, NHC-Pr^i-*C*H$_3$), 24.8, 25.4, 25.7, and 26.5 (Dipp-Pr^i-*C*H$_3$), 28.3 and 28.4 (Dipp-Pr^i-*C*H), 52.4 and 52.5 (NHC-Pr^i-*C*H), 124.3, 124.4, 125.5, and 125.6 (NHC-N*C*Me), 123.8, 127.1, 127.7, 128.4, 131.0, 135.5, 141.7, 148.2, and 168.2 (Ar-*C*), 200.7 and 200.8 (NHC-*C*:); ^{29}Si{^{1}H} NMR (C_6D_6, 80 MHz, 298 K): δ = 6.4 (*Si*Me$_3$), -32.9 (H-*Si*-L^{TMS}); anal. calcd. for $C_{53}H_{82}ClN_5NiSi_2$: C, 67.75 %; H, 8.80 %; N, 7.45 %; found: C, 67.89 %; H, 8.93%; N, 7.30 %.

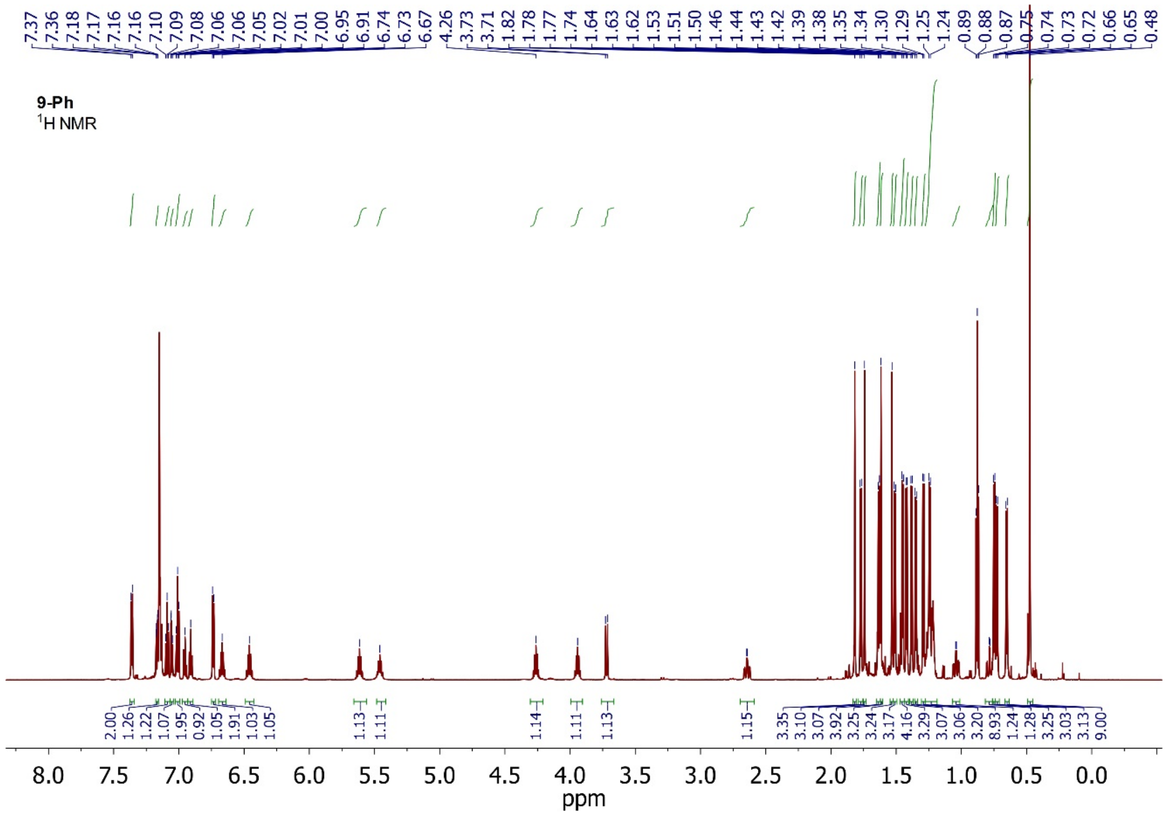

Figure S32. ^{1}H NMR spectrum of **9-Ph** dissolved in C_6D_6, at 298K.

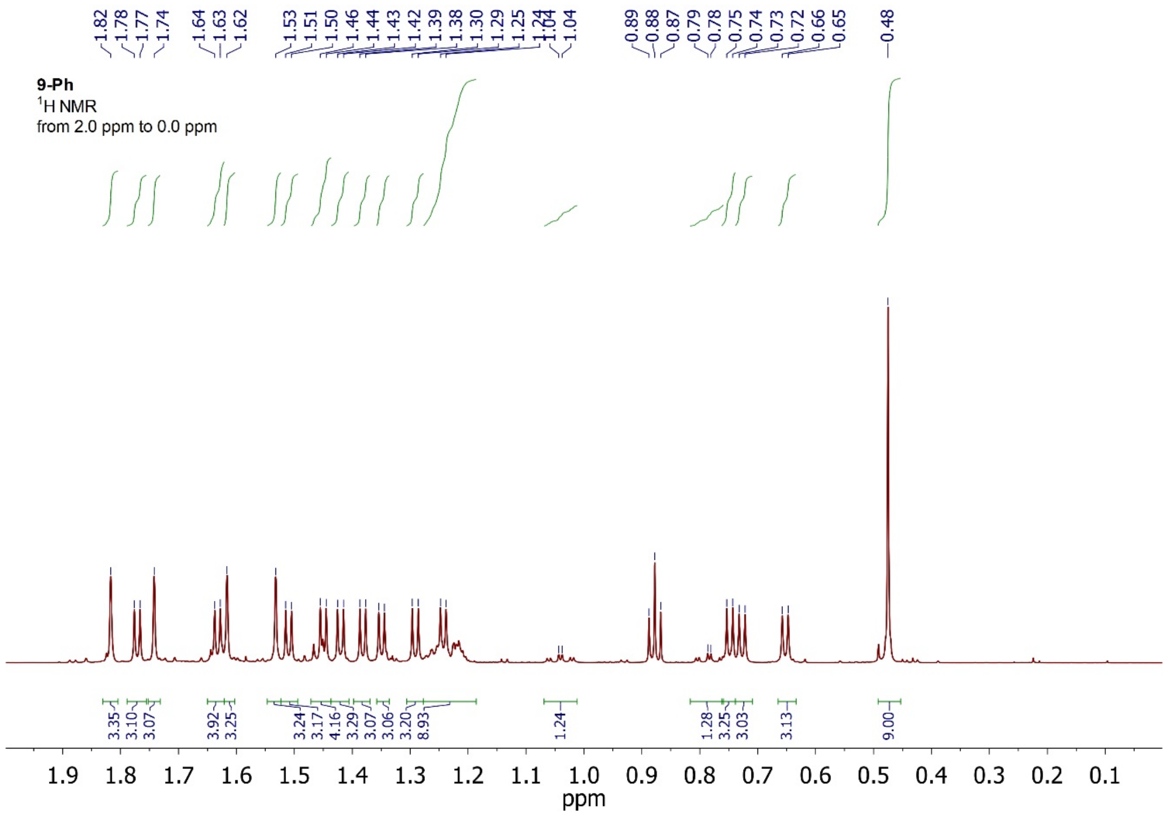

Figure S33. Zoom of the ^{1}H NMR spectrum of **9-Ph** dissolved in C_6D_6, from 0.0 ppm to2.0 ppm, at 298K.

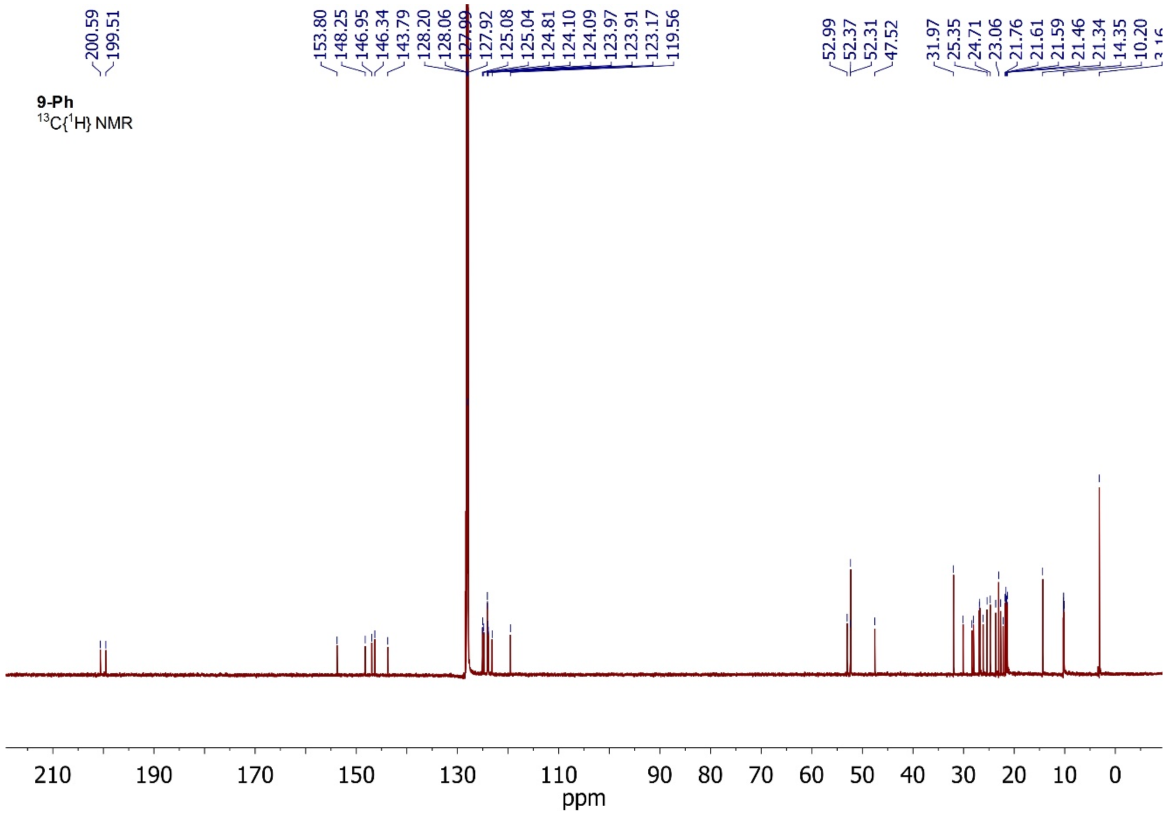

Figure S34. ^{13}C{^{1}H} NMR spectrum of **9** dissolved in C_6D_6, at 298K.

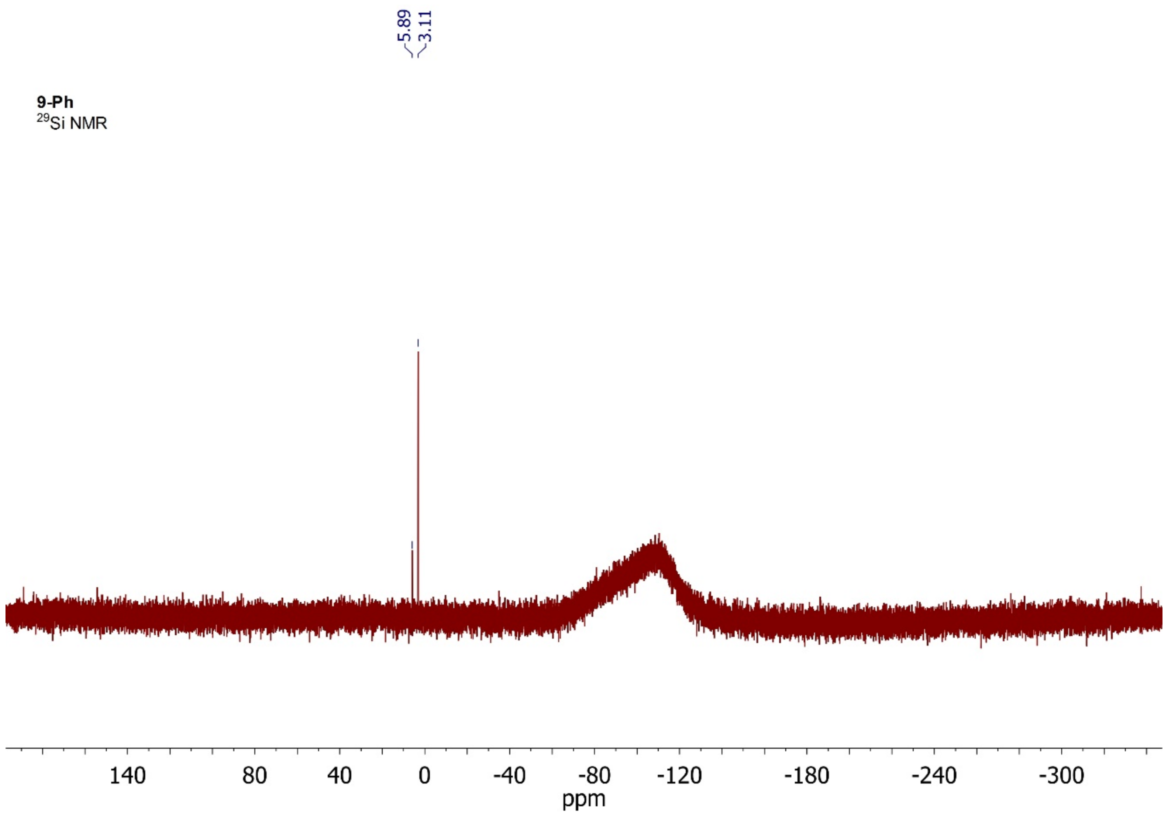

Figure S35. $^{29}Si\{^1H\}$ NMR spectrum of **9-Ph** dissolved in C_6D_6, at 298K.

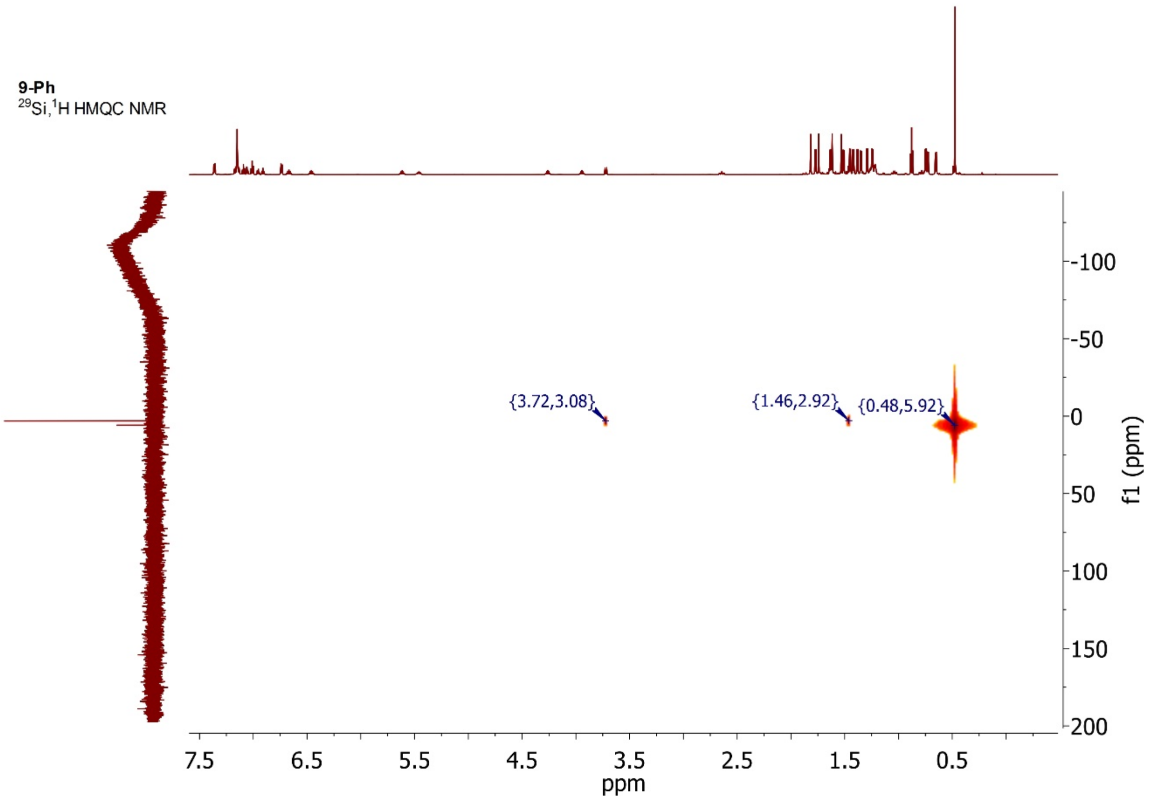

Figure S36. $^{29}Si,^1H$ HMQC NMR spectrum of **9-Ph** dissolved in C_6D_6, at 298K.

Synthesis of 10. A solution of **1** (0.15 g, 0.21 mmol) in toluene (10 mL) was cooled to -78 ^{o}C, and 2-butyne added *via* pipette (50 µL, 0.62 mmol). The reaction initially became bright yellow-orange, and slowly became pale yellow after stirring with warming to ambient temperature overnight. All volatiles were subsequently removed *in vacuo*, the reaction mixture extracted in hexane (15mL), and concentrated to ~2 mL, whereupon colorless crystals began to form. Storage of this sample at ambient temperature overnight allowed for the formation of large colorless crystals of **10** suitable for an X-ray diffraction analysis. (35 mg, 41 %). ^{1}H NMR (C_6D_6, 200 MHz, 298 K): δ = 0.24 (s, 9H, Si*Me*$_3$), 1.20 (d, $^{3}J_{HH}$ = 6.8 Hz, 6H, Dipp-Pr^{i}-C*H*$_3$), 1.29 (d, $^{3}J_{HH}$ = 6.8 Hz, 6H, Dipp-Pr^{i}-C*H*$_3$), 1.53 (br s, 6H, Si{C(Me)C(*Me*)}$_2$), 1.63 (br s, 6H, Si{C(*Me*)C(Me)}$_2$), 3.63 (sept, $^{3}J_{HH}$ = 6.8 Hz, 2H, Dipp-Pr^{i}-C*H*), 7.05 (m, 3H, Ar-C*H*); ^{13}C{^{1}H} NMR (C_6D_6, 75.5 MHz, 298 K): δ = 1.8 (Si*Me*$_3$), 13.2 and 13.7 (Si{C(*Me*)C(*Me*)}$_2$)), 24.9 and 25.0 (Dipp-Pr^{i}-*C*H$_3$), 27.9 (Dipp-Pr^{i}-*C*H), 125.6 and 126.2 (Si{*C*(Me)*C*(Me)}$_2$)), 123.9, 147.6, and 149.6 (Ar-*C*H); ^{29}Si{^{1}H} NMR (C_6D_6, 80 MHz, 298 K): δ = 7.7 (*Si*Me$_3$), -5.9 (*Si*{C(Me)C(Me)}$_2$)); anal. calcd. for $C_{23}H_{38}ClNSi_2$: C, 65.75 %; H, 9.12 %; N, 3.33 %; found: C, 65.46 %; H, 9.18 %; N, 3.32 %.

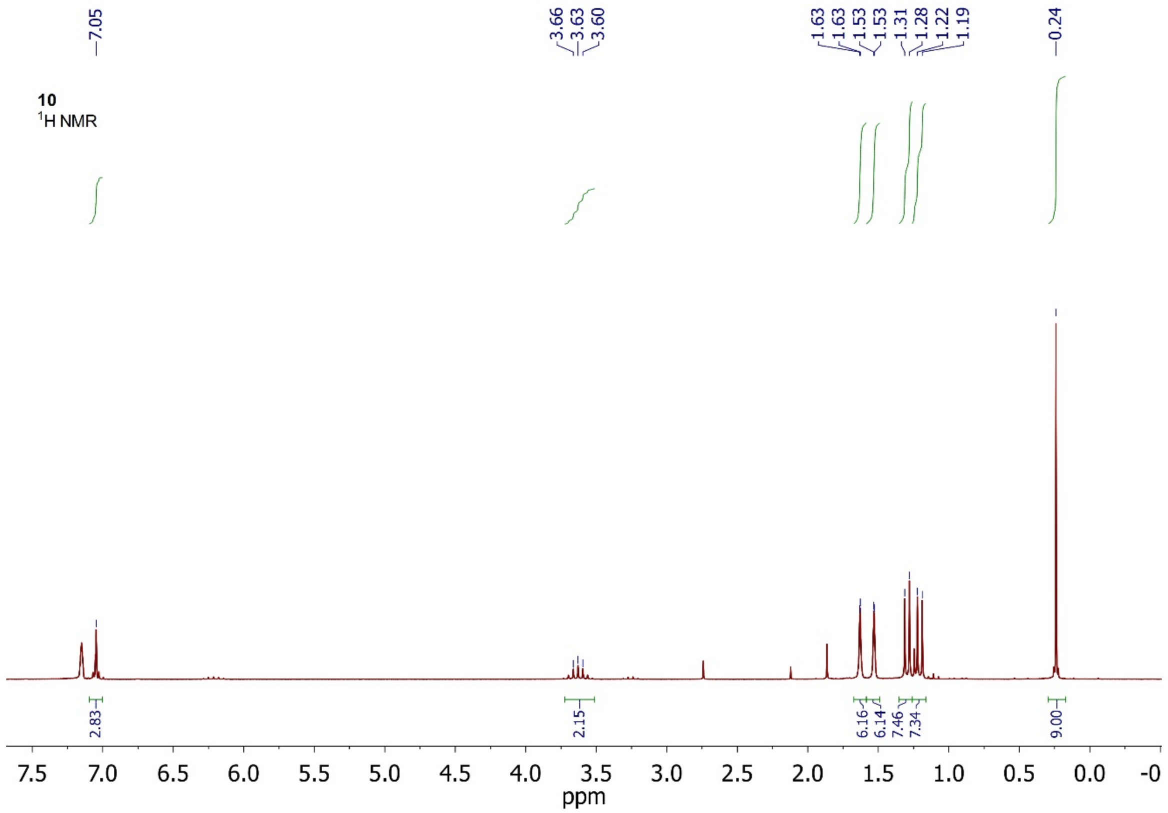

Figure S37. ^{1}H NMR spectrum of **10** dissolved in C_6D_6, at 298K.

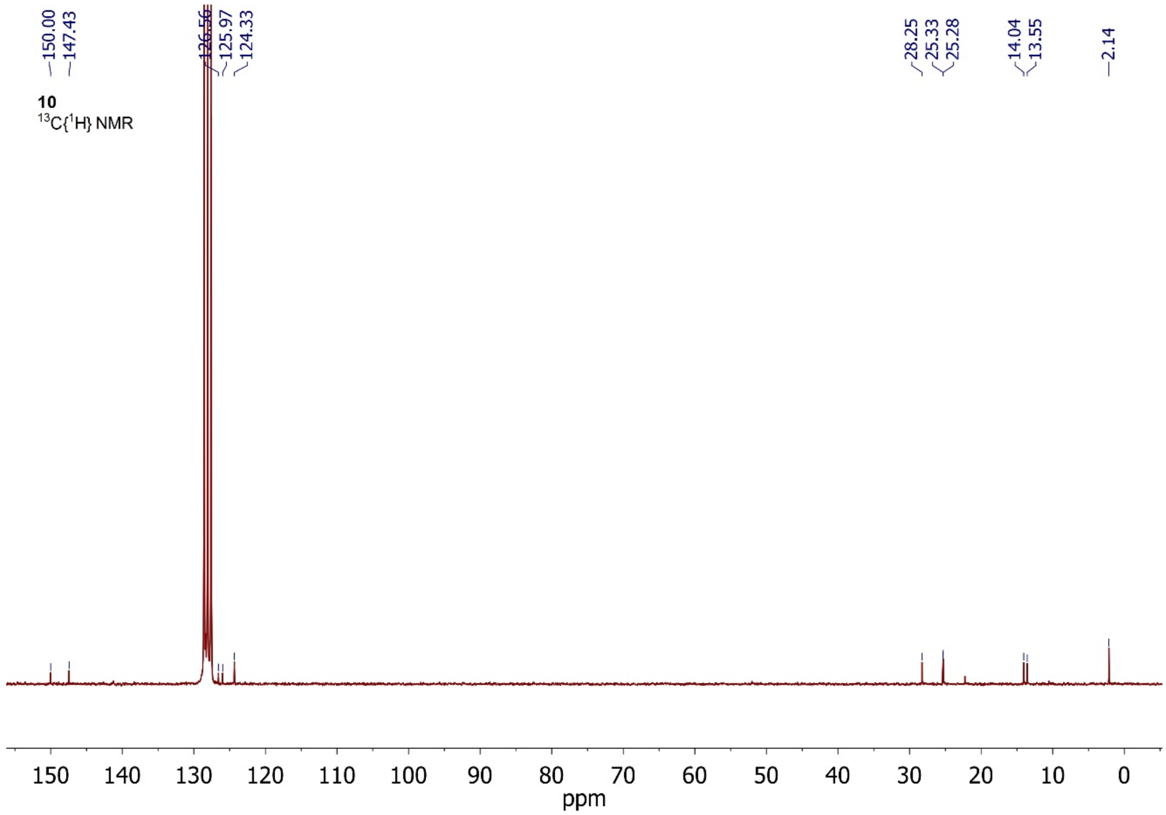

Figure S38. $^{13}C\{^1H\}$ NMR spectrum of **10** dissolved in C_6D_6, at 298K.

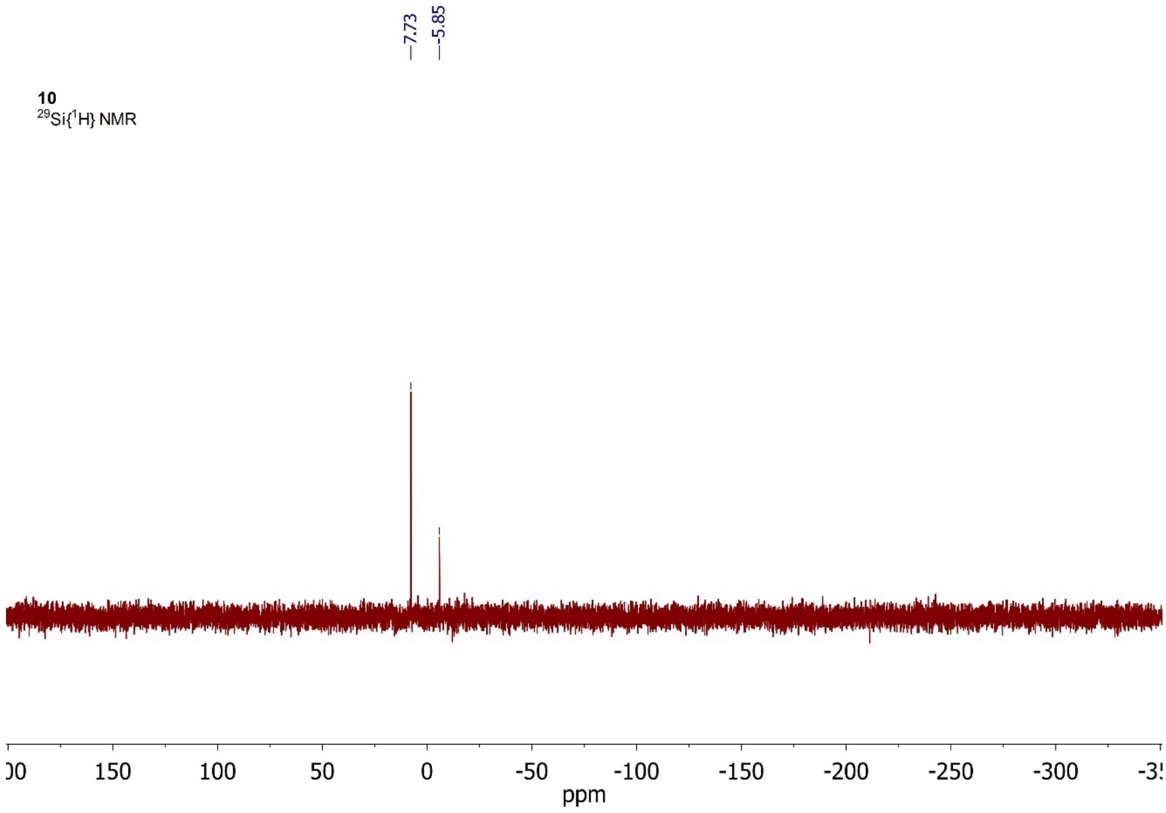

Figure S39. $^{29}Si\{^1H\}$ NMR spectrum of **10** dissolved in C_6D_6, at 298K.

2. X-Ray crystallographic data

Crystals were mounted on a glass capillary in perfluorinated oil and measured in a cold N_2 flow. The data was collected on an Oxford Diffraction SuperNova Atlas at 150 K (Cu-Kα radiation, λ= 1.54184 Å). The structures were solved by direct methods or using the SHELXT program[2] and refined on F^2 with the SHELX-2014 software package.[3] The positions of the H atoms were calculated and considered isotropically according to a riding model, aside from the Ni-H moiety found in **3**, which was located and freely refined. Full crystal data, refinement and data collection details for all crystallographically characterized compounds can be found in their CIFs.

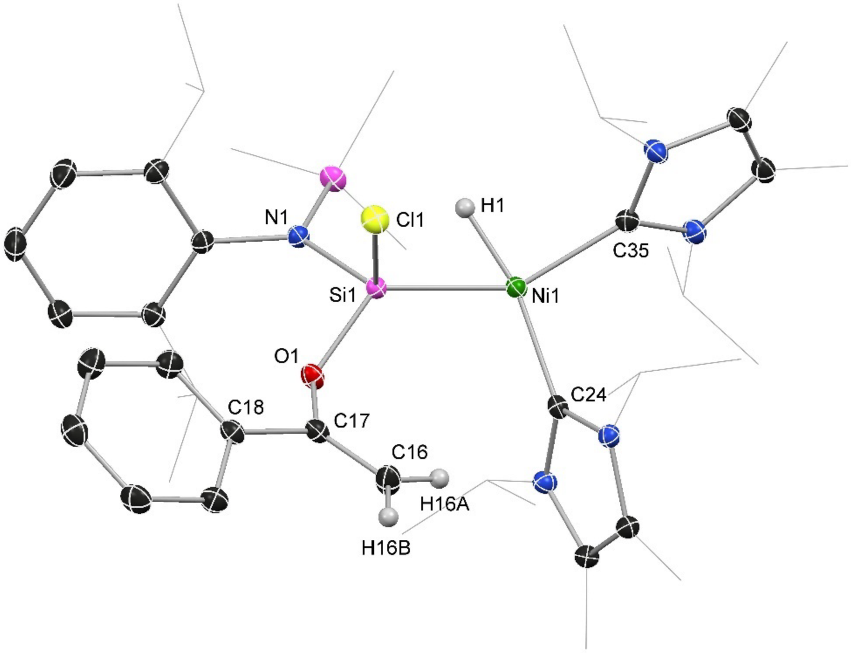

Figure S40. The molecular structure of **3**, with thermal ellipsoids at 30% probability.

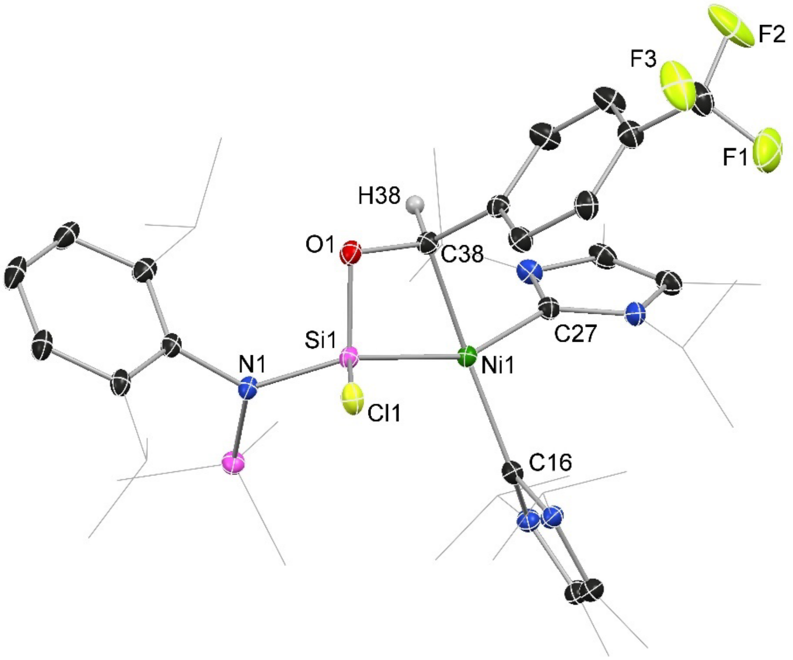

Figure S41. The molecular structure of **4**, with thermal ellipsoids at 30% probability.

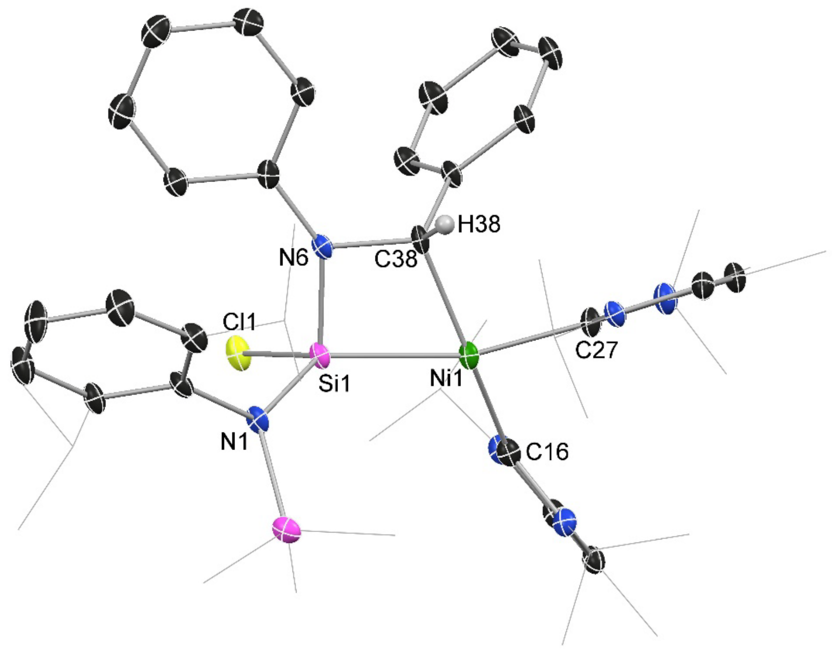

Figure S42. The molecular structure of **5**, with thermal ellipsoids at 30% probability.

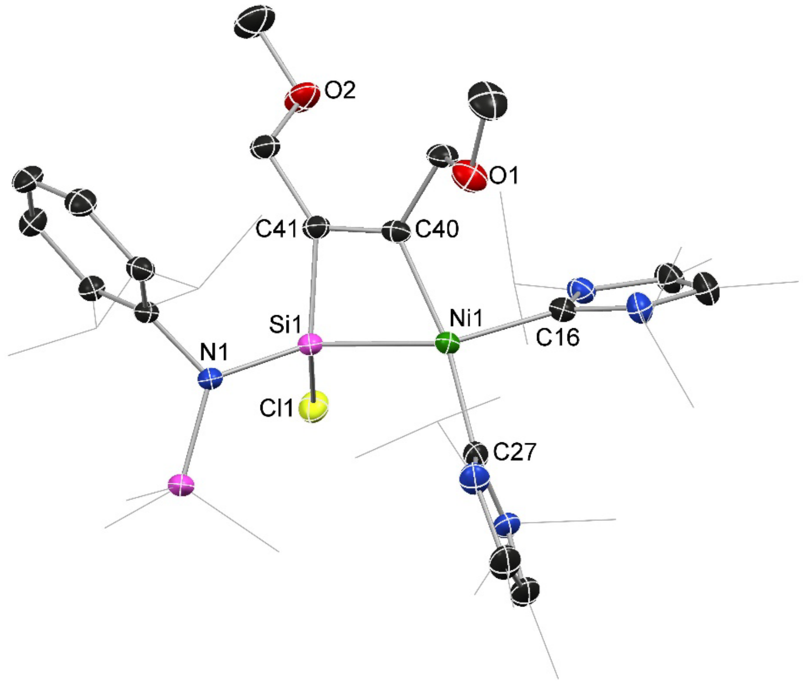

Figure S43. The molecular structure of **6-OMe**, with thermal ellipsoids at 30% probability.

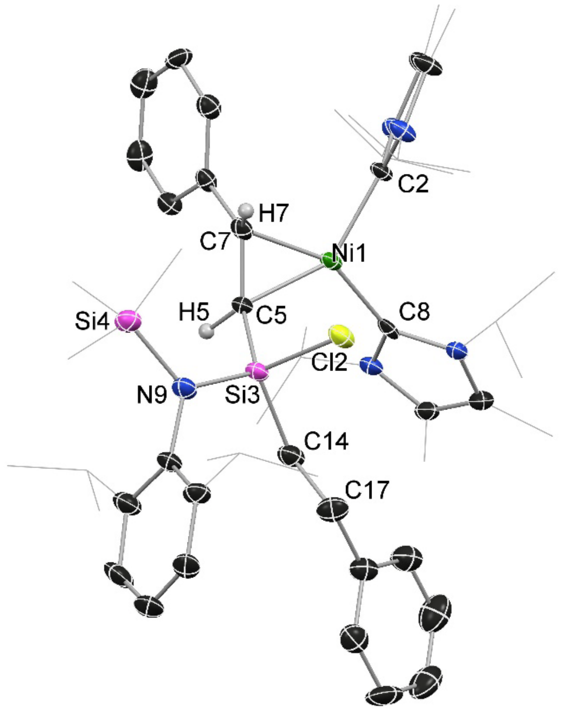

Figure S44. The molecular structure of **9-Ph**, with thermal ellipsoids at 30% probability.

Table S1. Summary of crystallographic data for compounds **3**, **4**, **5**, **6**, and **6-OMe**.

	**3**	**4**	**5**	**6**	**6-OMe**
empirical form.	$C_{45}H_{74}ClN_5NiOSi_2$	$C_{45}H_{71}ClF_3N_5NiOSi_2$	$C_{50}H_{77}ClN_6NiSi_2$	$C_{39}H_{68}ClN_5NiSi_2$	$C_{43}H_{76}ClN_5NiO_2Si_2$
formula wt	851.43	905.40	912.51	757.32	845.42
crystal syst.	triclinic	orthorhombic	orthorhombic	orthorhombic	monoclinic
space group	*P-1*	$P2_12_12_1$	$Pna2_1$	*Pbca*	$P2_1/n$
a (Å)	10.0535(3)	13.12070(10)	25.1576(5)	18.5613(6)	10.11490(10)
b (Å)	12.7880(4)	18.32810(10)	12.7701(3)	18.5497(5)	14. 27.8799(4)
c (Å)	20.3697(5)	19.8222(2)	15.4377(4)	24.7704(6)	17.5039(2)
α (deg.)	75.549(2)	90	90	90	90
β (δεγ)	87.147(2)	90	90	90	100.7680(10)
γ (deg.)	70.938(3)	90	90	90	90
vol (Å^3)	2395.46(13)	4766.79(7)	4959.6(2)	8528.6(4)	4849.22(10)
Z	2	4	4	8	4
ρ(calc) (g.cm^{-3})	1.180	1.262	1.222	1.180	1.158
μ (mm^{-1})	1.854	1.988	1.817	2.003	1.842
F(000)	920	1936	1968	3280	1832
T (K)	150(2)	150(2)	150(2)	150(2)	150(2)
reflns collect.	16804	19407	20162	60903	34731
unique reflns	9032	8502	8535	8086	9195
R_{int}	0.0221	0.0199	0.0672	0.0480	0.0379
R1 [$I>2\sigma(I)$]	0.0230	0.0242	0.0713	0.0253	0.0300
*w*R2 (all data)	0.0904	0.0846	0.2176	0.1082	0.1044
CCDC No.	1935411	1935412	1935413	1935414	1935415

Table S2. Summary of crystallographic data for compounds **7**, **9**, **9-Ph**, and **10**.

	**7**	**9**	**9-Ph**·1(hexane)	**10**
empirical form.	$C_{39}H_{70}ClN_5NiSi_2$	$C_{41}H_{74}ClN_5NiSi_2$	$C_{56}H_{89}ClN_5NiSi_2$	$C_{23}H_{38}ClNSi_2$
formula wt	759.34	787.39	982.66	420.17
crystal syst.	orthorhombic	monoclinic	monoclinic	monoclinic
space group	*Pbca*	$P2_1/c$	$P2_1/c$	*C2/c*
a (Å)	18.6232(5)	10.7905(2)	10.3865(2)	15.5485(3)
b (Å)	18.4205(6)	25.2369(5)	22.2433(6)	9.5907(2)
c (Å)	24.8498(6)	16.9414(4)	25.0818(5)	33.9484(7)
α (deg.)	90	90	90	90
β (δεγ)	90	92.780(2)	99.863(2)	104.255(2)
γ (deg.)	90	90	90	90
vol ($Å^3$)	8524.7(4)	4608.03(17)	5709.0(2)	4906.54(18)
Z	8	4	4	8
ρ(calc) ($g.cm^{-3}$)	1.183	1.135	1.143	1.138
μ (mm^{-1})	2.004	1.870	1.604	2.355
F(000)	3296	1712	2132	1824
T (K)	150(2)	150(2)	150(2)	150(2)
reflns collect.	33492	18612	22039	10172
unique reflns	8002	8692	10306	4624
R_{int}	0.0456	0.0416	0.1013	0.0220
R1 [$I > 2\sigma(I)$]	0.0334	0.0539	0.1296	0.0246
*w*R2 (all data)	0.1170	0.1324	0.2945	0.0908
CCDC No.	1935416	1935417	1935418	1935419

3. Computational methods and data

DFT calculations were performed at the B97-D/cc-pVTZ//B97-D/6-31G(d)[Ni: cc-pVTZ] level of theory,[4] with the GAUSSIAN 09.D01 software.[5] Stationary points were verified by harmonic vibrational frequency calculations. Transition states were analyzed by intrinsic reaction coordinate (IRC) calculations.

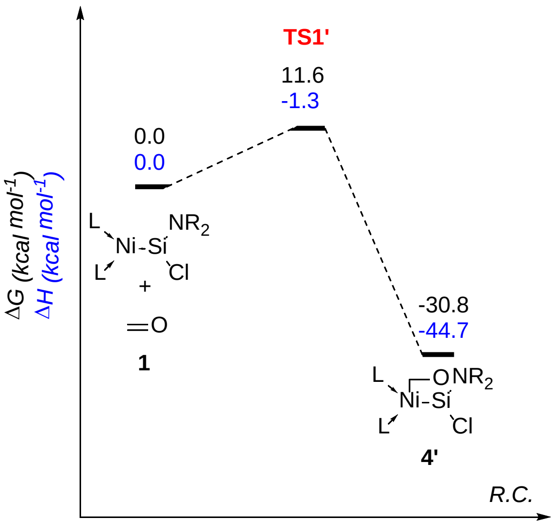

Figure S45. Reaction pathway for the addition of H_2CO to 1. L = NHC, NR_2 = ^{TMS}L.

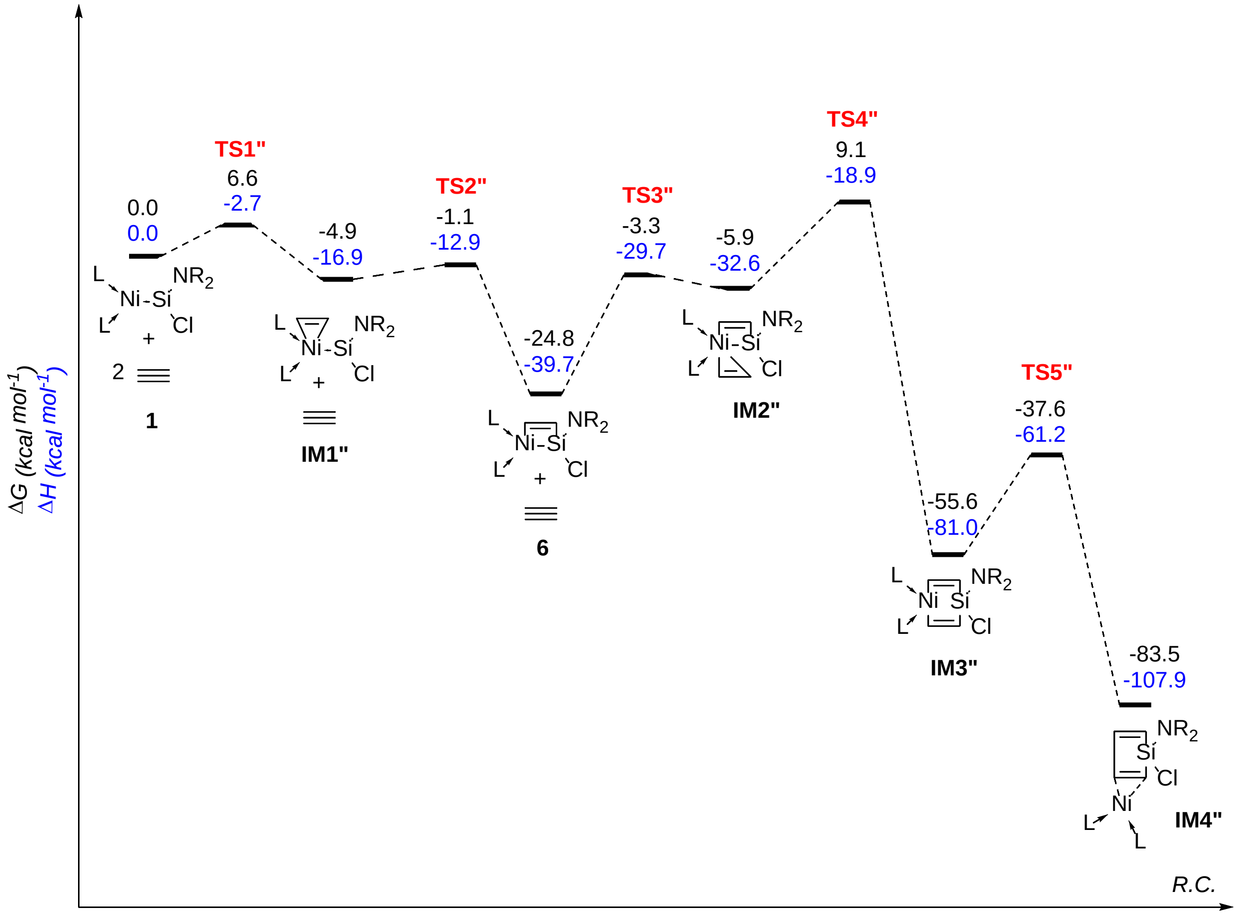

Figure S46. Reaction pathway for the addition of 2 equiv. C_2H_2 to **1**. L = NHC, NR_2 = ^{TMS}L.

Frontier orbitals of compound 6

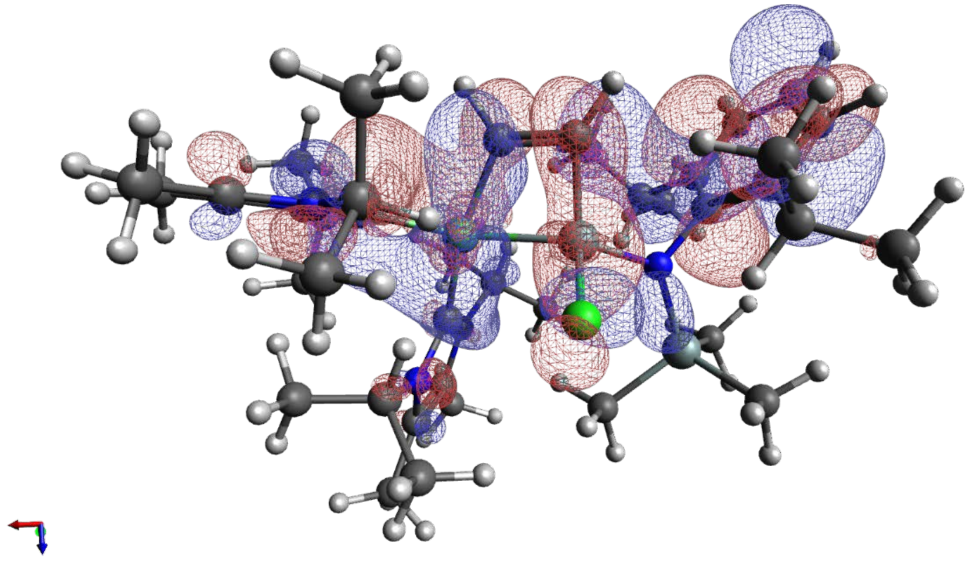

Figure S47. LUMO+1 (-0.181 eV).

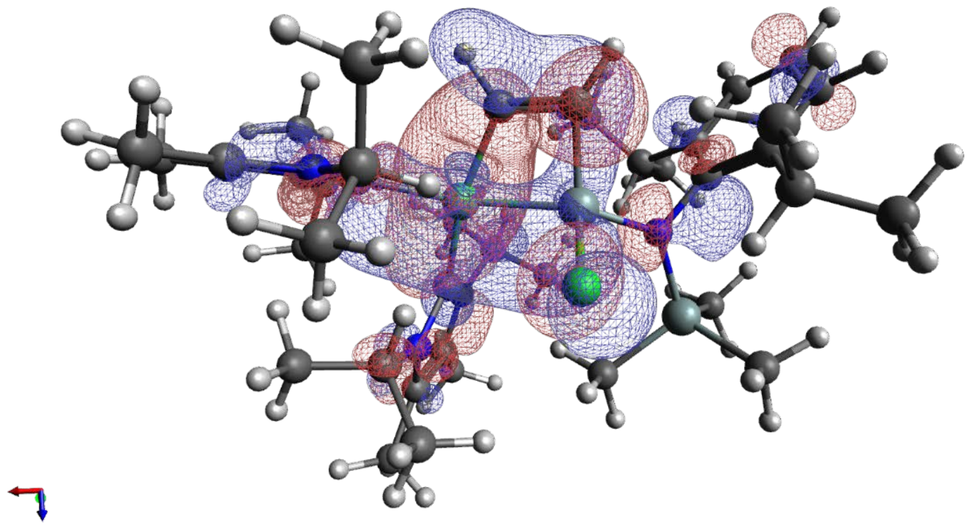

Figure S48. LUMO (-0.594 eV).

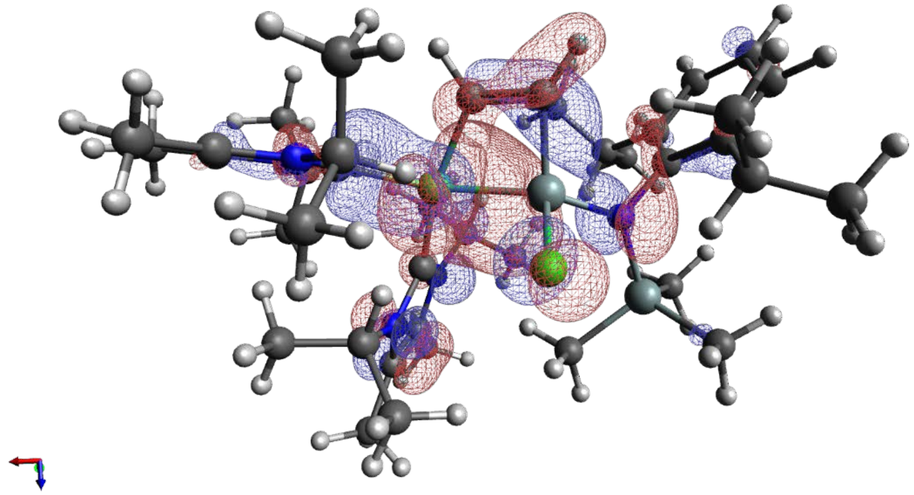

Figure S49. HOMO (-3.300 eV)

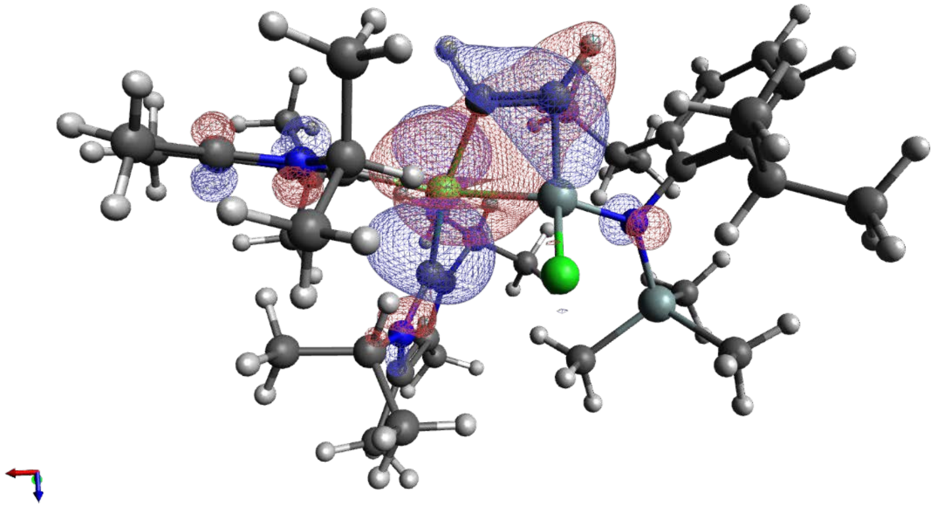

Figure S50. HOMO-2 (-3.408 eV).

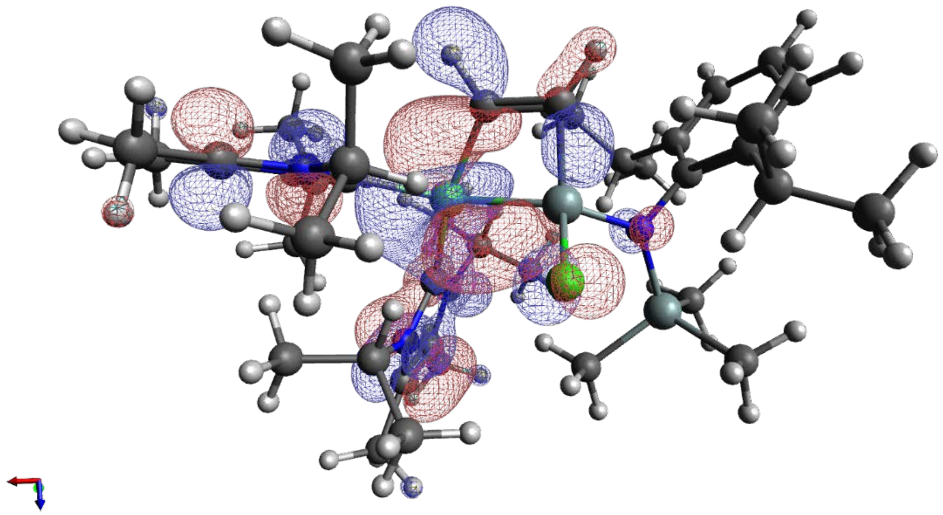

Figure S51. HOMO-3 (-3.812 eV).

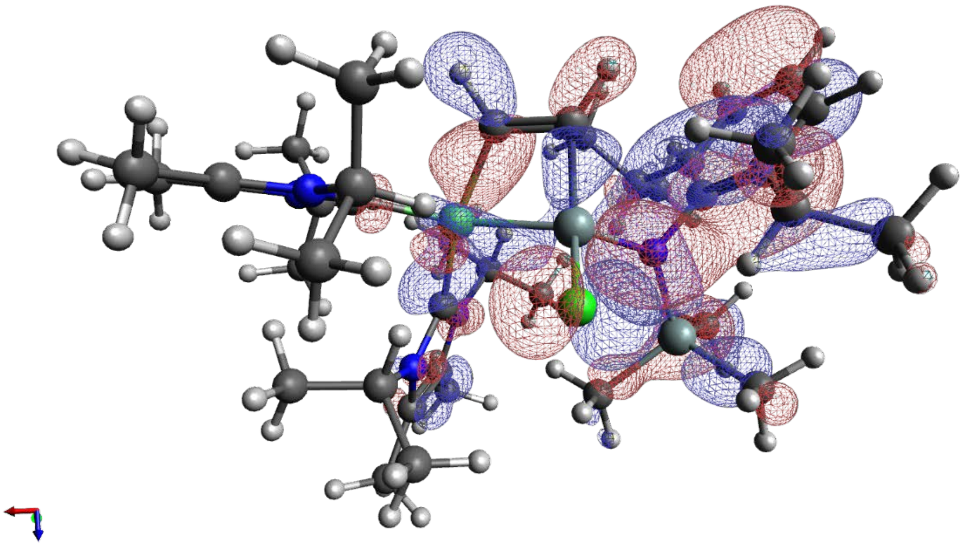

Figure S52. HOMO-5 (4.667 eV).

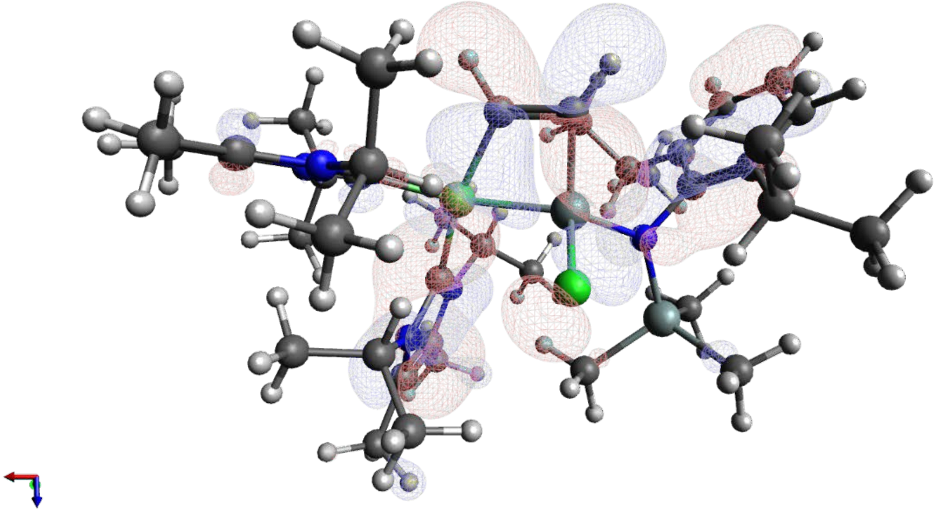

Figure S53. HOMO-7 (-4.846 eV).

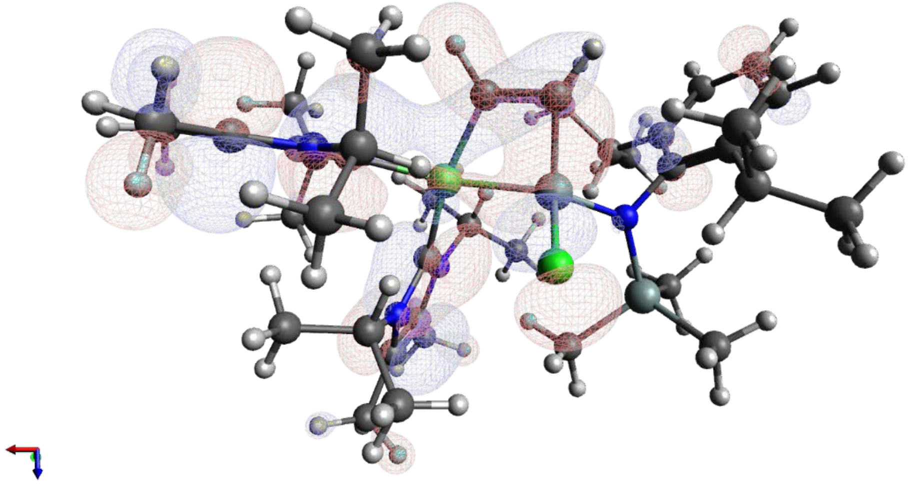

Figure S54. HOMO-9 (-5.144 eV).

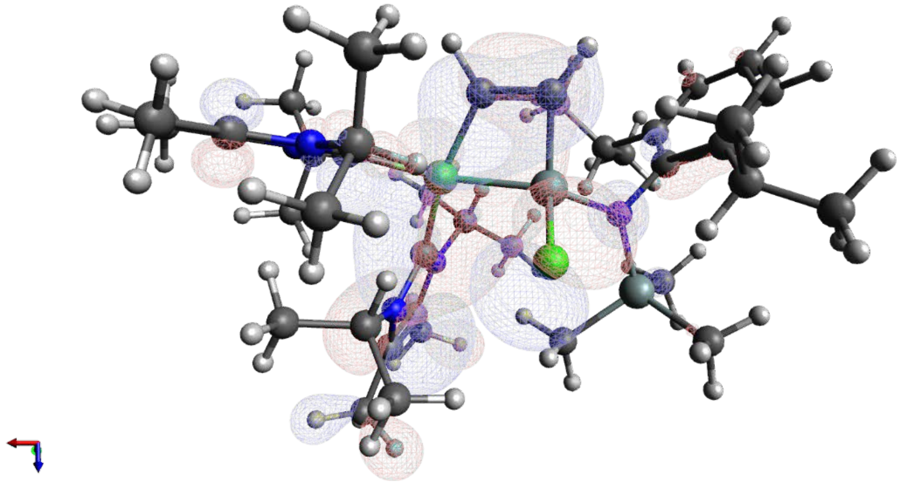

Figure S55. HOMO-10 (-5.487 eV).

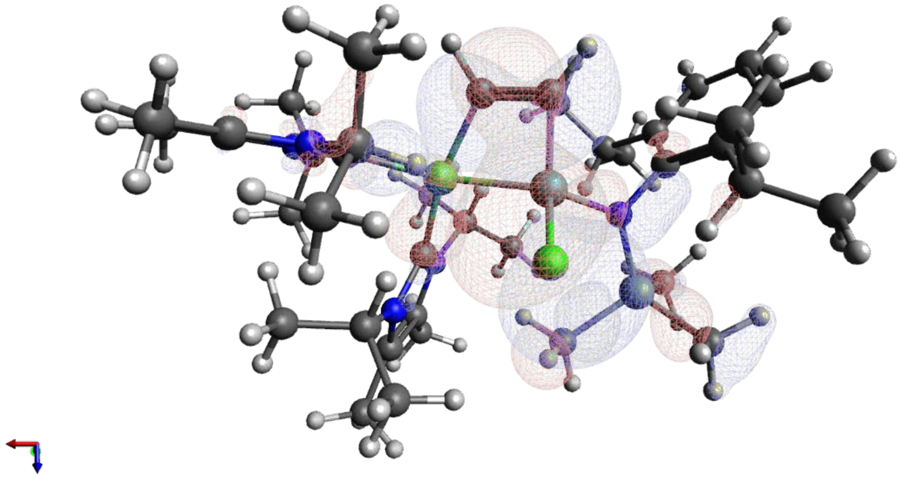

Figure S56. HOMO-12 (-6.031 eV).

Frontier orbitals of compound 7

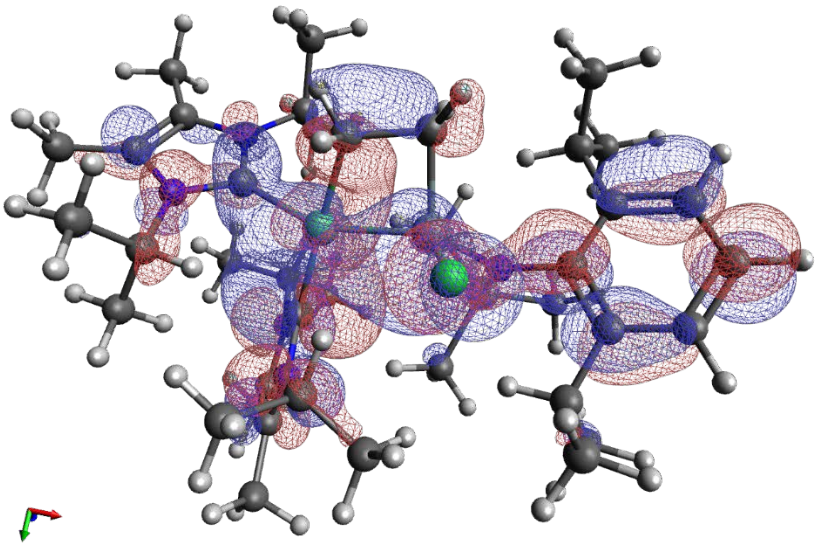

Figure S57. LUMO+1 (-0.120 eV).

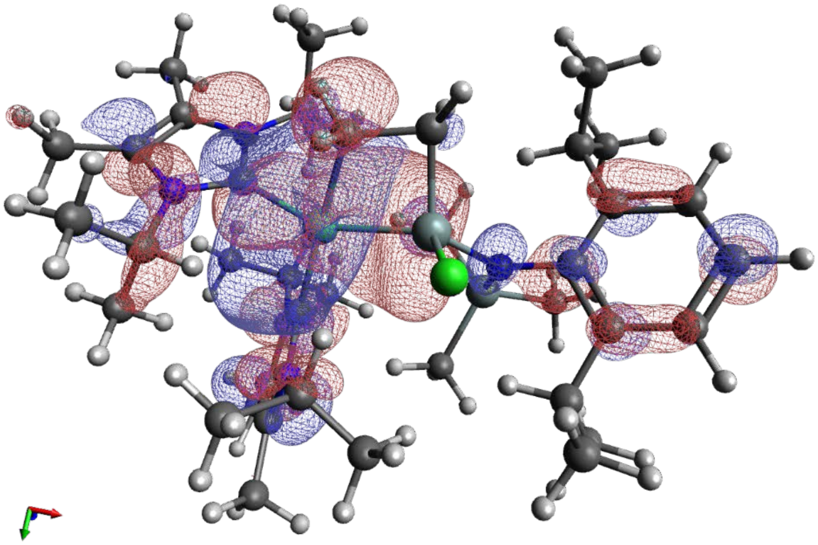

Figure S58. LUMO (-0.431 eV).

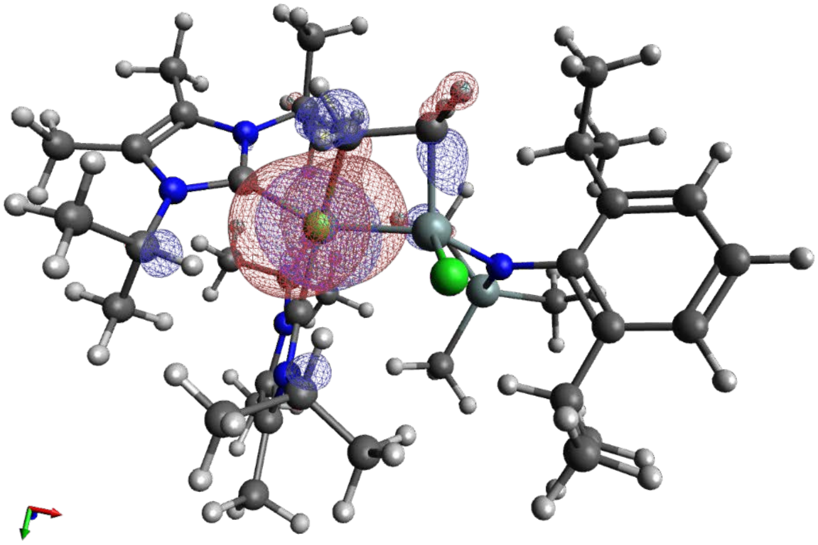

Figure S59. HOMO (-3.211 eV).

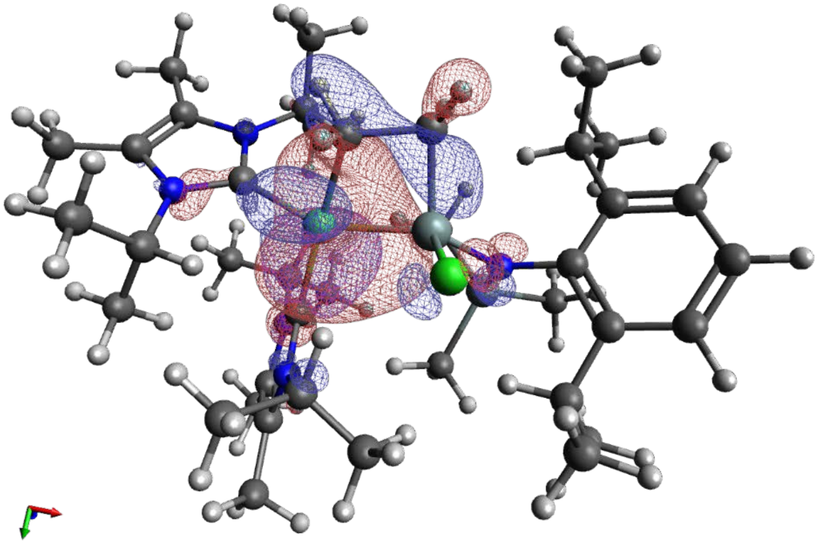

Figure S60. HOMO-1 (-3.466 eV).

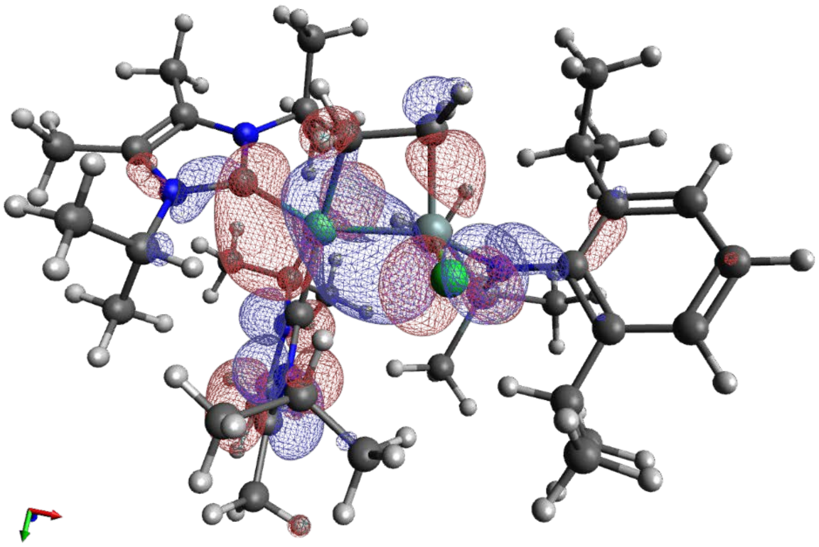

Figure S61. HOMO-2 (-3.579 eV).

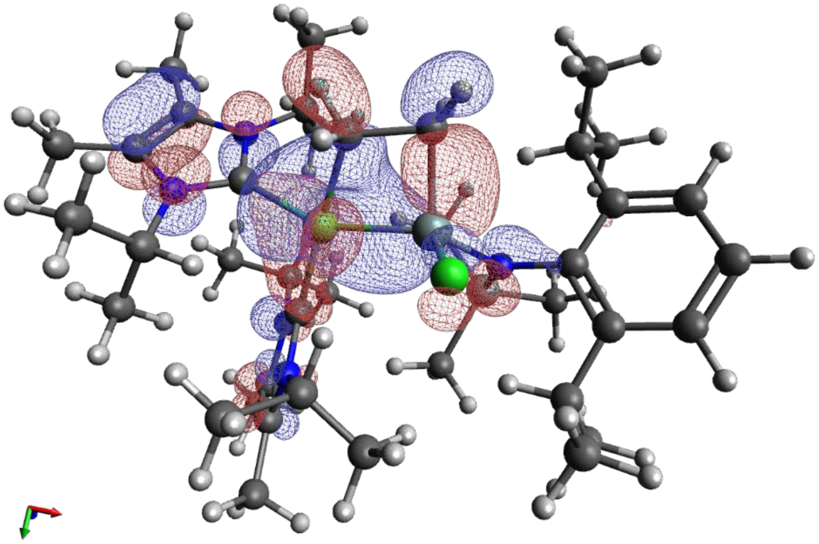

Figure S62. HOMO-3 (-3.699 eV).

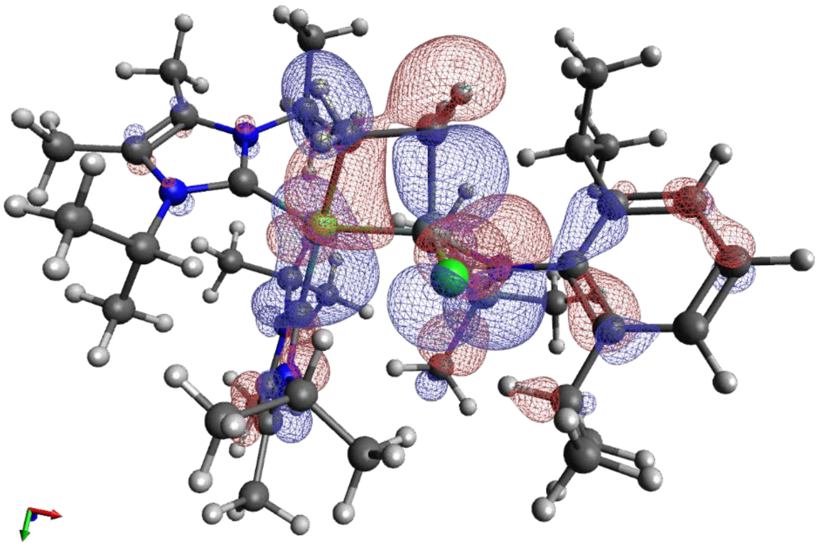

Figure S63. HOMO-4 (-4.190 eV).

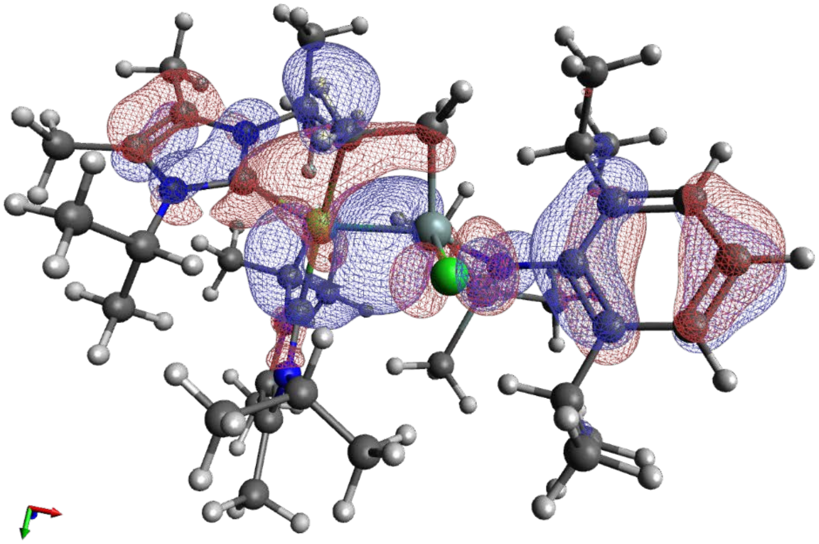

Figure S64. HOMO-5 (-4.331 eV).

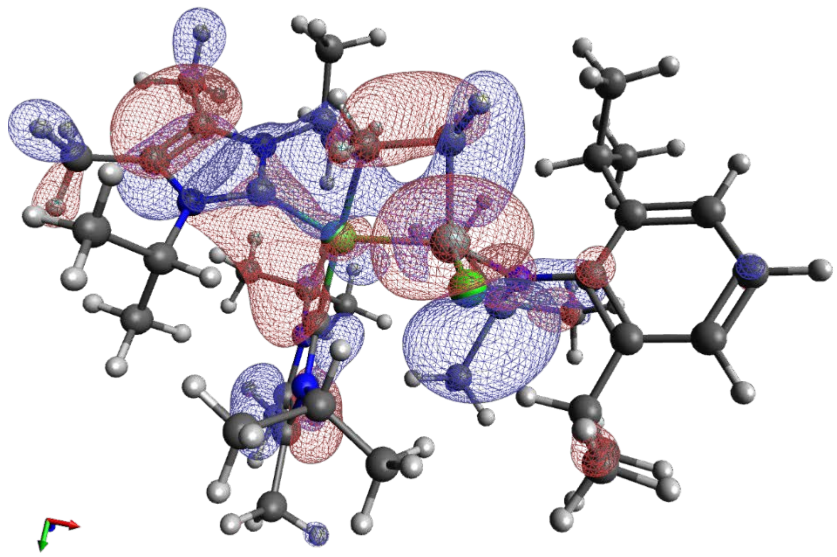

Figure S65. HOMO-11 (-5.692 eV).

Cartesian coordinates and energies

1

E(RB97D) = -3371.856956 E_h
Sum of electronic and thermal Enthalpies = -3371.41674 E_h
Sum of electronic and thermal Free Energies = -3371.41674 E_h
Number of imaginary frequencies 0

Ni	-0.75050	-0.04473	0.26808
Cl	1.29840	-0.80101	3.38397
Si	0.98035	-0.84012	1.20508
Si	2.83774	-0.63112	-1.16196
N	2.61456	-1.03843	0.56473
N	-3.68669	-0.53064	0.13594
N	-0.49037	2.39073	-1.38472
N	-0.29826	2.85649	0.71563
N	-2.60971	-2.22019	-0.66114
C	-2.39711	-0.96579	-0.11821
C	-0.52584	1.78699	-0.13643
C	-0.12405	4.05165	0.02503
C	-0.23287	3.75666	-1.30360
C	-3.96018	-2.54200	-0.73250
C	-0.63463	1.64135	-2.62354
H	-1.11429	2.27789	-3.38285
C	-1.52394	-3.09321	-1.08853
H	-0.64855	-2.85646	-0.46484
C	-4.64263	-1.47558	-0.21837
C	4.46378	-1.42228	-1.76338
H	5.34479	-1.01761	-1.23867
H	4.59853	-1.22701	-2.84153
H	4.46021	-2.51616	-1.61977
C	1.41572	-1.32123	-2.21863
H	1.38010	-2.42145	-2.15463

H	1.55810	-1.04612	-3.27954
H	0.43872	-0.93271	-1.88524
C	-3.98110	0.74584	0.77529
H	-3.12810	1.41280	0.58411
C	-0.28222	2.72279	2.16707
H	0.03922	1.70129	2.41201
C	2.94957	1.25686	-1.37835
H	2.01933	1.73592	-1.03548
H	3.12094	1.54065	-2.43228
H	3.78141	1.67194	-0.78324
H	-1.82600	-4.14196	-0.94914
H	-1.26847	-2.92044	-2.14586
H	-4.10464	0.62045	1.86309
H	-4.90443	1.16690	0.34789
H	-5.70481	-1.31264	-0.07138
H	-4.31130	-3.48423	-1.13892
H	0.42424	3.45086	2.59262
H	-1.28500	2.89685	2.59127
H	0.05378	4.99271	0.53396
H	-0.14497	4.38375	-2.18402
H	0.34435	1.29828	-2.99275
H	-1.26113	0.76334	-2.40792
C	3.86401	-1.27173	1.32562
H	4.51721	-0.37853	1.31545
H	3.63708	-1.50676	2.37352
H	4.43367	-2.11233	0.89509

TS1

E(RB97D) = -3450.389516 E_h
Sum of electronic and thermal Enthalpies = -3449.894436 E_h
Sum of electronic and thermal Free Energies = -3450.001962 E_h
Number of imaginary frequencies 1, v = -38.8 cm^{-1}

Ni	-0.97793	0.04723	-0.21208
Cl	0.61521	0.95255	2.87226
Si	0.73311	-0.37889	1.06516
Si	3.50952	-1.38663	0.07212
N	-3.29045	-1.15654	1.25534
N	0.64259	2.13338	-1.58153
N	2.41547	-0.07781	0.54480
N	-3.78448	-0.83667	-0.81938
N	-0.68438	2.92467	-0.06855
C	-2.74224	-0.68922	0.07824
C	-4.60481	-1.57873	1.09036
C	-0.30715	1.72769	-0.65882
C	-1.65398	3.01877	1.01774
H	-2.31594	2.14434	0.94430
C	-4.91911	-1.37896	-0.22468
C	-0.81998	-2.59033	-1.71375
H	-0.49512	-2.86156	-0.70857
H	-1.85243	-2.83009	-1.97712
C	0.83993	3.50874	-1.55864
C	0.01006	-1.98358	-2.57848
H	-0.30946	-1.69832	-3.58473

H	1.03824	-1.74761	-2.30078
C	-2.54962	-1.18686	2.51520
H	-1.62310	-1.76440	2.38062
C	0.00736	4.00646	-0.59690
C	1.37049	1.19834	-2.42846
H	0.70220	0.75900	-3.18353
C	-3.67115	-0.48465	-2.22828
H	-2.81294	0.19486	-2.32972
C	4.16372	-1.08030	-1.69861
H	3.37283	-1.22005	-2.45501
H	4.98050	-1.78312	-1.93933
H	4.56324	-0.05884	-1.81906
C	5.00428	-1.42430	1.25265
H	5.54464	-0.46251	1.25176
H	5.72381	-2.21135	0.96658
H	4.67798	-1.62023	2.28806
C	2.62497	-3.06794	0.10934
H	2.23974	-3.30504	1.11417
H	3.34026	-3.86032	-0.17660
H	1.77812	-3.11493	-0.59275
C	3.05128	1.25454	0.67895
H	3.61552	1.34818	1.62289
H	3.74677	1.44308	-0.16039
H	2.28835	2.04634	0.66295
H	-2.26563	-0.16896	2.81436
H	-3.18326	-1.64754	3.28623
H	-4.59299	0.01634	-2.56200
H	-3.49779	-1.38024	-2.84691
H	-5.83492	-1.56366	-0.77548
H	-5.19451	-1.96729	1.91343
H	-1.14344	2.99646	1.99138
H	-2.23015	3.95043	0.90684
H	-0.15565	5.02055	-0.24973
H	1.53883	4.00221	-2.22481
H	1.77751	0.40316	-1.79227
H	2.18991	1.73536	-2.92665

IM1

E(RB97D) = -3450.404154 E_h
Sum of electronic and thermal Enthalpies = -3449.906798 E_h
Sum of electronic and thermal Free Energies = -3450.00789 E_h
Number of imaginary frequencies 0

Ni	0.62083	0.08553	-0.70345
Cl	-0.71625	-3.02181	0.90321
Si	-1.02860	-0.81685	0.62142
Si	-3.82606	0.47381	0.31421
N	0.91448	2.95589	0.25856
N	2.47630	-2.25886	-0.33127
N	-2.68060	-0.82834	-0.06074
N	2.77732	2.15087	-0.45473
N	2.56611	-0.90170	1.33731
C	1.44879	1.81262	-0.29077
C	1.87153	3.94669	0.45598
C	1.91212	-1.09357	0.13582

C	2.27742	0.19251	2.26019
H	2.84898	1.09540	1.99478
C	3.05435	3.43648	0.00303
C	-0.49501	0.66449	-2.28690
H	-1.50608	0.96538	-2.00516
H	0.05025	1.39442	-2.89553
C	3.43436	-2.76626	0.53785
C	-0.18497	-0.71877	-2.37596
H	0.63190	-1.04508	-3.03094
H	-0.96641	-1.46911	-2.24118
C	-0.49546	3.09576	0.60021
H	-1.02027	2.23779	0.16648
C	3.49267	-1.90601	1.59578
C	2.02744	-2.94004	-1.54103
H	2.37715	-2.40919	-2.43883
C	3.78627	1.23628	-0.98053
H	3.27710	0.49417	-1.60639
C	-3.88125	1.80370	-1.05688
H	-2.95830	2.40470	-1.09548
H	-4.72663	2.49547	-0.89226
H	-4.01526	1.34477	-2.05133
C	-5.57679	-0.27050	0.44658
H	-5.93864	-0.65866	-0.51972
H	-6.29141	0.49989	0.78510
H	-5.60354	-1.09928	1.17396
C	-3.41250	1.30158	1.97792
H	-3.54159	0.58584	2.80669
H	-4.09480	2.15306	2.15339
H	-2.37917	1.67385	2.02672
C	-3.22446	-1.86794	-0.96711
H	-3.50250	-1.43534	-1.94735
H	-2.47621	-2.65355	-1.13906
H	-4.11896	-2.35311	-0.53977
H	-0.63296	3.08927	1.69276
H	-0.88936	4.03685	0.18655
H	4.30422	0.71194	-0.16174
H	4.51502	1.80378	-1.57798
H	4.04542	3.87331	-0.05572
H	1.62650	4.91592	0.87647
H	1.20429	0.41404	2.20479
H	2.54405	-0.12775	3.27756
H	4.08343	-1.92797	2.50463
H	3.96591	-3.68920	0.33574
H	0.93261	-2.96839	-1.53675
H	2.43125	-3.96193	-1.53625

TS2

E(RB97D) = -3450.376761 E_h
Sum of electronic and thermal Enthalpies = -3449.881862 E_h
Sum of electronic and thermal Free Energies = -3449.98274 E_h
Number of imaginary frequencies 1, v = -314.6 cm^{-1}

Ni	-1.03650	-0.09591	-0.42886
Cl	1.62986	2.74993	-0.03655
Si	1.12360	0.58971	-0.07774

Si	3.16180	-1.64449	0.56281
N	-2.14774	-2.75339	-0.04126
N	-2.02623	2.62404	0.38291
N	2.69891	-0.20359	-0.33429
N	-3.70933	-1.34035	-0.52903
N	-1.89045	1.27993	2.06426
C	-2.34267	-1.42238	-0.35473
C	-3.34488	-3.46078	-0.02110
C	-1.75195	1.31349	0.69769
C	-1.53082	0.09848	2.83965
H	-2.09990	-0.76657	2.47226
C	-4.33103	-2.57125	-0.33571
C	-0.19473	-0.84303	-2.05398
H	0.50483	-1.68279	-1.95122
H	-0.99704	-1.06628	-2.77406
C	-2.26515	3.38900	1.52118
C	0.44631	0.50300	-2.08771
H	-0.18239	1.31115	-2.48551
H	1.42346	0.54932	-2.59499
C	-0.84213	-3.33646	0.25322
H	-0.10179	-2.53226	0.16253
C	-2.17905	2.53743	2.58545
C	-1.89727	3.15957	-0.96796
H	-2.12857	2.35302	-1.67703
C	-4.40039	-0.12966	-0.95925
H	-3.83381	0.73226	-0.58841
C	2.91365	-3.21303	-0.50649
H	1.85621	-3.34882	-0.78873
H	3.24589	-4.12655	0.01850
H	3.49433	-3.14058	-1.44264
C	5.01692	-1.55587	1.00856
H	5.66322	-1.55316	0.11496
H	5.30929	-2.42557	1.62275
H	5.24060	-0.64280	1.58591
C	2.17845	-1.82595	2.18375
H	2.23629	-0.90100	2.78072
H	2.61039	-2.64851	2.78330
H	1.11401	-2.04461	2.01485
C	3.60625	0.19933	-1.43033
H	3.48060	-0.43093	-2.33336
H	3.41838	1.24665	-1.71328
H	4.66199	0.13225	-1.11697
H	-0.82895	-3.74575	1.27556
H	-0.61430	-4.13882	-0.46521
H	-5.41647	-0.12597	-0.53785
H	-4.45921	-0.08558	-2.05886
H	-5.40331	-2.69787	-0.43521
H	-3.38656	-4.51621	0.22356
H	-0.45676	-0.10287	2.69753
H	-1.76104	0.28021	3.89902
H	-2.30898	2.70627	3.64860
H	-2.48104	4.45003	1.46960
H	-0.86607	3.50201	-1.13862
H	-2.60489	3.99182	-1.09956

7

E(RB97D) = -3450.412696 E_h
Sum of electronic and thermal Enthalpies = -3449.916141 E_h
Sum of electronic and thermal Free Energies = -3450.017285 E_h
Number of imaginary frequencies 0

Ni	0.65383	0.00460	-0.51682
Cl	-2.16011	-2.65393	-1.05293
Si	-1.41434	-0.57491	-1.15015
Si	-2.98629	1.07513	1.04078
N	2.11387	2.54252	-0.04937
N	1.31862	-2.71015	0.40771
N	-2.76867	0.40382	-0.56413
N	3.48113	0.99666	-0.66978
N	1.52694	-1.31756	2.04199
C	2.15196	1.20508	-0.38088
C	3.37104	3.13604	-0.12953
C	1.16429	-1.36991	0.70900
C	1.62297	-0.09135	2.82421
H	1.69558	0.74663	2.12180
C	4.23635	2.15975	-0.53265
C	-0.01576	0.94078	-2.20467
H	-0.46738	1.92417	-1.99267
H	0.89326	1.11236	-2.81098
C	1.73188	-3.45091	1.50837
C	-1.04831	-0.01523	-2.90866
H	-0.51620	-0.79014	-3.48501
H	-1.79093	0.46935	-3.56787
C	0.89777	3.22200	0.38855
H	0.04371	2.61461	0.06642
C	1.85865	-2.57371	2.54478
C	1.17831	-3.27566	-0.93221
H	0.81388	-2.48301	-1.59571
C	3.99783	-0.28264	-1.14363
H	3.30482	-1.06433	-0.80865
C	-3.16665	2.97485	0.91996
H	-2.25277	3.43274	0.50383
H	-3.35759	3.43140	1.90748
H	-4.00362	3.25778	0.25869
C	-4.56731	0.37717	1.84675
H	-5.46373	0.60686	1.24638
H	-4.72736	0.79391	2.85675
H	-4.50133	-0.72045	1.93619
C	-1.49016	0.66786	2.12890
H	-1.37303	-0.41984	2.25460
H	-1.59485	1.13096	3.12680
H	-0.56284	1.03596	1.66499
C	-3.98989	0.40449	-1.40429
H	-3.72626	0.33593	-2.47176
H	-4.65628	-0.44379	-1.16332
H	-4.56189	1.34137	-1.26957
H	0.88017	3.32525	1.48580
H	0.84610	4.21964	-0.07197
H	4.99843	-0.45506	-0.71906
H	4.05814	-0.29619	-2.24359
H	5.30091	2.18865	-0.73769
H	3.53419	4.18167	0.10730

H	0.73615	0.04426	3.45951
H	2.52797	-0.13393	3.44976
H	2.14516	-2.72406	3.57980
H	1.89526	-4.52140	1.45333
H	0.44763	-4.09481	-0.92284
H	2.15718	-3.64034	-1.28588

TS3

E(RB97D) = -3528.932355 E_h
Sum of electronic and thermal Enthalpies = -3528.378246 E_h
Sum of electronic and thermal Free Energies = -3528.479471 E_h
Number of imaginary frequencies 1, v = -109.3 cm^{-1}

Ni	0.45059	-0.22640	-0.67947
Cl	-2.36725	-0.42585	1.96722
Si	-1.76621	-0.47047	-0.20392
Si	-3.52739	1.96972	-1.22706
N	2.83568	-2.14742	-0.33106
N	0.60842	2.49019	0.80142
N	-3.15760	0.24509	-1.06181
N	2.17245	-1.22273	1.49653
N	2.33882	2.06544	-0.40337
C	1.93138	-1.21505	0.13902
C	3.59496	-2.70863	0.69266
C	0.06268	0.77860	-2.88944
H	-0.97344	0.44672	-2.94054
H	0.22239	1.82262	-2.62996
C	1.10643	1.52626	-0.05775
C	1.09133	0.00542	-3.33243
H	2.10791	0.38772	-3.41471
H	0.91863	-1.00826	-3.69599
C	3.31324	1.45517	-1.29942
H	3.07175	0.39257	-1.38742
C	3.17353	-2.12089	1.84914
C	-0.35474	-2.00395	-1.33688
H	-0.58753	-1.81819	-2.39772
H	0.39934	-2.80490	-1.28027
C	1.48435	3.55670	0.97250
C	-1.65894	-2.32186	-0.55586
H	-1.43951	-2.86476	0.37741
H	-2.41747	-2.89078	-1.12341
C	2.98654	-2.51693	-1.73177
H	2.37473	-1.82599	-2.31959
C	2.57703	3.29173	0.20486
C	-0.64587	2.40775	1.54253
H	-1.43955	2.05497	0.88313
C	1.44143	-0.39344	2.45339
H	0.38490	-0.37110	2.16107
C	-4.29883	2.65364	0.38175
H	-5.21824	2.10110	0.63953
H	-4.56902	3.71974	0.27630
H	-3.61659	2.55331	1.24133
C	-2.01099	3.02259	-1.68749
H	-1.10005	2.75058	-1.13980
H	-2.23607	4.08395	-1.48161

H	-1.79166	2.92934	-2.76286
C	-4.80726	2.19865	-2.62463
H	-4.47966	1.69496	-3.55024
H	-4.92830	3.27234	-2.85189
H	-5.80065	1.80258	-2.35801
C	-4.40474	-0.56924	-1.00024
H	-4.99774	-0.35985	-0.08959
H	-5.03883	-0.37409	-1.88162
H	-4.16303	-1.64179	-0.99530
H	4.04511	-2.43730	-2.02732
H	2.63651	-3.54682	-1.90092
H	1.84249	0.63127	2.45785
H	1.54427	-0.83733	3.45358
H	3.48031	-2.26549	2.87907
H	4.34661	-3.46786	0.50712
H	3.27400	1.92578	-2.29413
H	4.32119	1.58419	-0.87619
H	3.48575	3.85642	0.02879
H	1.24516	4.39566	1.61586
H	-0.89569	3.41169	1.91273
H	-0.56212	1.70712	2.38193

IM2

E(RB97D) = -3528.939723 E_h
Sum of electronic and thermal Enthalpies = -3528.384341 E_h
Sum of electronic and thermal Free Energies = -3528.485495 E_h
Number of imaginary frequencies 0

Ni	0.38869	-0.27001	-0.88078
Cl	-2.38453	0.50321	2.10105
Si	-1.76964	-0.32557	0.11054
Si	-3.94425	1.15358	-1.73000
N	2.45553	-2.30871	0.04155
N	1.05218	2.61186	0.05147
N	-3.24600	-0.22395	-0.88844
N	1.89952	-0.94439	1.61215
N	2.77410	1.53667	-0.65909
C	1.63515	-1.22670	0.28976
C	3.19399	-2.67583	1.16236
C	-0.32794	0.38675	-2.71269
H	-1.31715	-0.04190	-2.87689
H	-0.28244	1.47720	-2.76177
C	1.40505	1.40078	-0.50095
C	0.83669	-0.36484	-2.96531
H	1.76429	0.14857	-3.23049
H	0.74284	-1.39288	-3.32594
C	3.68793	0.49386	-1.11977
H	3.11394	-0.23214	-1.69802
C	2.84348	-1.80890	2.15504
C	-0.60994	-2.06888	-1.01370
H	-1.25087	-2.01534	-1.90996
H	0.12454	-2.87266	-1.16468
C	2.14606	3.44763	0.24181
C	-1.44051	-2.18646	0.28727
H	-0.77026	-2.36845	1.14295
H	-2.23374	-2.95490	0.28157

C	2.62711	-2.94434	-1.25929
H	2.10488	-2.32748	-1.99690
C	3.23784	2.76631	-0.20647
C	-0.28920	2.98814	0.48349
H	-0.95882	2.17225	0.20766
C	1.32242	0.17591	2.35326
H	0.33377	0.39477	1.94107
C	-5.67701	1.54729	-1.02806
H	-6.37299	0.69710	-1.12324
H	-6.13109	2.40598	-1.55402
H	-5.60775	1.80132	0.04324
C	-2.90235	2.73003	-1.55101
H	-2.91879	3.09699	-0.51293
H	-3.33629	3.51706	-2.19265
H	-1.85539	2.58700	-1.85082
C	-4.13967	0.74037	-3.58503
H	-3.15760	0.62792	-4.07458
H	-4.69699	1.52989	-4.11982
H	-4.68933	-0.20636	-3.72580
C	-4.16129	-1.39163	-0.81766
H	-3.69283	-2.29219	-1.25076
H	-4.45154	-1.62129	0.22260
H	-5.09179	-1.20970	-1.38304
H	3.70056	-3.00120	-1.50040
H	2.19992	-3.95872	-1.25746
H	1.96554	1.06552	2.26292
H	1.22747	-0.10932	3.41024
H	3.15860	-1.73870	3.19010
H	3.88048	-3.51516	1.15233
H	4.47011	0.95362	-1.74236
H	4.15118	-0.01558	-0.26028
H	4.28524	3.04190	-0.25963
H	2.04207	4.44228	0.66042
H	-0.59579	3.91795	-0.01564
H	-0.30908	3.12019	1.57523

TS4

E(RB97D) = -3528.912393 E_h
Sum of electronic and thermal Enthalpies = -3528.359667 E_h
Sum of electronic and thermal Free Energies = -3528.462209 E_h
Number of imaginary frequencies 1, v = -191.9 cm^{-1}

Ni	0.60671	-0.31410	-0.85850
Cl	-2.34167	0.24824	1.94957
Si	-1.98089	-0.29408	-0.21336
Si	-4.57751	1.34248	-0.93018
N	2.46933	-2.21410	0.38999
N	1.23630	2.58206	-0.06019
N	-3.63600	-0.14306	-0.86281
N	1.74798	-0.79857	1.85351
N	2.97146	1.46139	-0.66106
C	1.56460	-1.17527	0.53943
C	3.17220	-2.46067	1.56560
C	-0.99346	0.60337	-2.13278
H	-1.85020	0.07329	-2.56462

H	-1.20323	1.66599	-1.98736
C	1.60537	1.34174	-0.52702
C	0.29850	0.27886	-2.74758
H	0.92676	1.12047	-3.05642
H	0.31260	-0.56215	-3.44821
C	3.82571	0.38326	-1.14458
H	3.22989	-0.21716	-1.84417
C	2.71698	-1.56609	2.48847
C	-0.29710	-2.12214	-1.25995
H	-0.66702	-2.11381	-2.29920
H	0.35247	-2.99862	-1.13538
C	2.32945	3.43095	0.09524
C	-1.45897	-2.13435	-0.25543
H	-1.08928	-2.39451	0.75094
H	-2.26721	-2.84590	-0.51252
C	2.80823	-2.86312	-0.87416
H	2.31375	-2.30350	-1.67696
C	3.43018	2.71846	-0.28182
C	-0.13757	2.98590	0.21502
H	-0.75033	2.08051	0.27321
C	1.10965	0.35710	2.47406
H	0.27839	0.66339	1.83727
C	-5.99941	1.29028	0.33745
H	-6.65238	0.41530	0.17704
H	-6.63235	2.19331	0.27714
H	-5.59260	1.21972	1.35973
C	-3.48973	2.85795	-0.55607
H	-3.01515	2.77371	0.43384
H	-4.12217	3.76308	-0.55173
H	-2.70380	3.00823	-1.31275
C	-5.32266	1.53972	-2.67752
H	-4.52526	1.64328	-3.43323
H	-5.97005	2.43219	-2.74089
H	-5.93627	0.66747	-2.96120
C	-4.42172	-1.38371	-1.05291
H	-3.90810	-2.08213	-1.73572
H	-4.60963	-1.91350	-0.10049
H	-5.40443	-1.16342	-1.50589
H	3.90130	-2.83997	-1.00857
H	2.46021	-3.90683	-0.88374
H	1.83651	1.17986	2.57031
H	0.72019	0.07988	3.46370
H	2.97656	-1.41733	3.53066
H	3.91439	-3.24803	1.63633
H	4.69877	0.81743	-1.65400
H	4.15935	-0.25584	-0.31304
H	4.47975	2.98770	-0.32202
H	2.22041	4.45236	0.44237
H	-0.52147	3.62870	-0.59068
H	-0.17887	3.52981	1.17003

8

E(RB97D) = -3528.969959 E_h
Sum of electronic and thermal Enthalpies = -3528.415458 E_h
Sum of electronic and thermal Free Energies = -3528.51941 E_h
Number of imaginary frequencies 0

Ni	0.81215	-0.17976	-0.74447
Cl	-2.31267	0.66431	1.76841
Si	-2.56186	-0.16135	-0.24482
Si	-5.36352	0.77581	-1.24922
N	2.59748	-2.44480	0.04175
N	1.70904	2.54062	0.16875
N	-4.27954	-0.39978	-0.47291
N	1.97629	-1.27271	1.73938
N	3.30848	1.40990	-0.72208
C	1.83281	-1.34603	0.37272
C	3.19955	-3.01948	1.15989
C	-1.86010	1.14806	-1.42954
H	-2.55108	1.24830	-2.28828
H	-1.92996	2.10790	-0.89148
C	1.96665	1.31993	-0.41401
C	-0.39107	0.92685	-1.91728
H	0.04428	1.92354	-2.11302
H	-0.41691	0.39774	-2.88651
C	4.05609	0.31624	-1.33020
H	3.38320	-0.21732	-2.01239
C	2.80473	-2.27565	2.23472
C	-0.40186	-1.72039	-1.19520
H	-0.76980	-1.61629	-2.23276
H	0.13008	-2.68853	-1.14397
C	2.84244	3.34777	0.22518
C	-1.61333	-1.80004	-0.23230
H	-1.27033	-2.02985	0.79151
H	-2.31240	-2.61246	-0.52257
C	2.79623	-2.90593	-1.32670
H	2.14174	-2.30784	-1.97236
C	3.85743	2.63056	-0.33909
C	0.39559	2.93853	0.66192
H	-0.27702	2.08532	0.54649
C	1.37913	-0.20571	2.53945
H	0.43212	0.08994	2.07350
C	-7.14625	0.42336	-0.67099
H	-7.50537	-0.56420	-1.00500
H	-7.82922	1.18310	-1.08886
H	-7.23637	0.45914	0.42746
C	-4.91860	2.56094	-0.76128
H	-4.78725	2.64312	0.33040
H	-5.72427	3.25354	-1.06315
H	-3.98643	2.90282	-1.23643
C	-5.30931	0.59396	-3.14457
H	-4.29818	0.77080	-3.54664
H	-5.99443	1.31079	-3.63138
H	-5.61301	-0.42273	-3.44740
C	-4.90034	-1.46826	0.34723
H	-4.17829	-2.27827	0.53942
H	-5.24817	-1.09062	1.32614
H	-5.76256	-1.91558	-0.17313
H	3.84817	-2.76886	-1.62749
H	2.52928	-3.97025	-1.41257
H	2.05166	0.66590	2.58161
H	1.19159	-0.57914	3.55640

H	3.01769	-2.38014	3.29300
H	3.82876	-3.90025	1.09213
H	4.91079	0.72790	-1.88675
H	4.41420	-0.38728	-0.56227
H	4.89938	2.87716	-0.51178
H	2.82067	4.34755	0.64480
H	0.01295	3.79332	0.08297
H	0.45813	3.21448	1.72600

TS1'

E(RB97D) = -3486.289415 E_h
Sum of electronic and thermal Enthalpies = -3485.817662 E_h
Sum of electronic and thermal Free Energies = -3485.919982 E_h
Number of imaginary frequencies 1, v = -213.0 cm^{-1}

Ni	-0.74596	0.26002	0.03469
Si	0.70603	-1.12356	0.75645
Si	2.98676	-0.36448	-1.02264
Cl	0.44631	-1.79968	2.79661
O	0.47430	-3.34084	-0.08044
N	-3.54264	-0.57530	0.35258
C	-0.17741	2.02426	0.37250
N	0.16243	3.00277	-0.54703
N	-3.08070	-0.49090	-1.74951
N	0.03463	2.66257	1.58110
N	2.40293	-1.25902	0.40325
C	-2.38678	-0.23628	-3.00504
H	-1.30767	-0.30064	-2.81804
C	-0.25897	2.04744	2.86889
H	-0.11395	0.96402	2.77633
C	-2.52765	-0.22343	-0.51353
C	-0.55281	-2.62895	-0.37538
H	-0.72997	-2.28337	-1.41845
C	0.57798	4.18131	0.06845
C	0.16965	2.76874	-1.98206
H	-0.62545	2.04493	-2.20901
C	-4.67969	-1.01382	-0.31784
C	-4.38982	-0.95894	-1.65120
C	1.71332	-0.46912	-2.43808
H	1.52507	-1.52581	-2.69162
H	2.06275	0.05217	-3.34779
H	0.75244	-0.02600	-2.12617
C	0.48416	3.96904	1.41280
C	3.33625	1.45219	-0.57855
H	2.44569	1.93431	-0.14968
H	3.65693	2.03564	-1.46061
H	4.14242	1.51252	0.17263
C	-3.39861	-0.47393	1.80291
H	-2.41501	-0.87032	2.09117
C	4.60192	-1.17411	-1.62156
H	5.41416	-1.08331	-0.88164
H	4.94191	-0.68630	-2.55165
H	4.45983	-2.24626	-1.83498
C	3.24678	-2.35751	0.93601
H	4.30195	-2.04402	0.98516
H	2.92259	-2.61945	1.95249

H	3.16605	-3.25954	0.30747
H	-1.49110	-2.70580	0.21848
H	-4.19442	-1.06006	2.28403
H	-3.46844	0.57839	2.12091
H	-5.57068	-1.34195	0.20607
H	-4.97931	-1.22361	-2.52202
H	-2.67672	-0.99527	-3.74649
H	-2.63300	0.76599	-3.39503
H	-1.30010	2.24960	3.17136
H	0.42636	2.45012	3.62911
H	0.68922	4.62103	2.25462
H	0.90473	5.04592	-0.49842
H	-0.02191	3.71649	-2.50740
H	1.13517	2.35191	-2.30684

4’

E(RB97D) = -3486.348472 E_h
Sum of electronic and thermal Enthalpies = -3485.874081 E_h
Sum of electronic and thermal Free Energies = -3485.972521 E_h
Number of imaginary frequencies 0

Ni	-0.62421	-0.12206	0.59016
Si	1.43935	0.04333	1.35374
Si	2.77882	-0.89280	-1.26736
Cl	2.22288	1.96607	2.02909
O	1.17995	-0.84503	2.76505
N	-3.59967	-0.31599	0.48776
C	-0.79763	1.30476	-0.67049
N	-0.98369	1.27248	-2.03725
N	-2.70894	-2.22019	0.02243
N	-0.83488	2.65555	-0.39123
N	2.78551	-0.67497	0.47422
C	-1.72831	-3.26973	-0.24255
H	-0.77054	-2.94542	0.17677
C	-0.79066	3.21647	0.95649
H	-0.55803	2.40480	1.65450
C	-2.37321	-0.92848	0.36112
C	-0.17303	-1.16191	2.24293
H	-0.26331	-2.25847	2.14167
C	-1.10375	2.54830	-2.58162
C	-0.99711	0.04344	-2.81825
H	-1.37751	-0.76049	-2.17642
C	-4.65200	-1.18687	0.22169
C	-4.08756	-2.39381	-0.07634
C	1.39119	-2.11844	-1.72363
H	1.60916	-3.11000	-1.29092
H	1.25253	-2.23986	-2.81290
H	0.44332	-1.76607	-1.28616
C	-1.01886	3.42191	-1.53713
C	2.51182	0.74942	-2.20022
H	1.61242	1.27239	-1.84189
H	2.41126	0.59332	-3.29000
H	3.36976	1.42320	-2.03518
C	-3.74930	1.10221	0.80074
H	-2.90806	1.40187	1.43611

C	4.44658	-1.63084	-1.82047
H	5.29324	-0.97342	-1.56133
H	4.45241	-1.76726	-2.91611
H	4.63416	-2.61634	-1.36191
C	4.01237	-0.90182	1.27694
H	4.64618	-1.67367	0.81291
H	4.60917	0.02217	1.38419
H	3.74156	-1.25358	2.28592
H	-0.92346	-0.81166	2.97917
H	-4.69788	1.25682	1.33485
H	-3.73612	1.70750	-0.11914
H	-5.69011	-0.88027	0.28837
H	-4.53424	-3.35003	-0.32516
H	-2.05426	-4.20579	0.23465
H	-1.61332	-3.43120	-1.32617
H	-1.76552	3.66720	1.20679
H	0.00080	3.97500	1.02316
H	-1.07214	4.50433	-1.50366
H	-1.22820	2.71328	-3.64601
H	-1.65725	0.17674	-3.68815
H	0.01714	-0.21374	-3.15362

TS1”

E(RB97D) = -3449.127882 E_h
Sum of electronic and thermal Enthalpies = -3448.657474 E_h
Sum of electronic and thermal Free Energies = -3448.763281 E_h
Number of imaginary frequencies 1, v = -97.0 cm^{-1}

Ni	0.98489	0.06614	0.26550
Cl	-0.47732	-0.23868	-2.98490
Si	-0.70394	-0.81834	-0.82379
Si	-3.49203	-1.35753	0.38612
N	3.26508	-1.61785	-0.78963
N	-0.82717	2.36656	0.85336
N	-2.38686	-0.30969	-0.52011
N	3.86556	-0.40388	0.88774
N	0.50976	2.72112	-0.80819
C	2.75956	-0.70501	0.11361
C	4.61655	-1.86802	-0.58117
C	0.18509	1.75779	0.13377
C	1.50015	2.51937	-1.86178
H	2.19992	1.74717	-1.51298
C	4.99722	-1.10222	0.48453
C	-1.10710	3.64322	0.38266
C	2.46875	-2.21446	-1.86056
H	1.54579	-2.63395	-1.43489
C	-0.26879	3.86269	-0.67338
C	-1.50477	1.73291	1.97507
H	-0.98397	1.94473	2.92193
C	3.82310	0.52497	2.01108
H	2.98471	1.21471	1.84090
C	-4.66276	-0.28325	1.44533
H	-4.09925	0.35897	2.14167
H	-5.32521	-0.92911	2.04756
H	-5.30713	0.36688	0.83060

C	-4.55765	-2.40183	-0.79914
H	-5.09992	-1.76255	-1.51700
H	-5.30717	-3.00136	-0.25279
H	-3.92661	-3.09287	-1.38330
C	-2.51794	-2.50175	1.55319
H	-1.82930	-3.16557	1.00559
H	-3.22004	-3.13784	2.12166
H	-1.92225	-1.92532	2.27950
C	-3.01657	0.84396	-1.20183
H	-3.63178	0.53179	-2.06530
H	-3.66232	1.40345	-0.50179
H	-2.24730	1.53480	-1.57323
H	2.18122	-1.45215	-2.59709
H	3.06297	-3.00395	-2.34215
H	4.77138	1.08200	2.05957
H	3.65441	-0.01226	2.95643
H	5.95624	-0.99142	0.97850
H	5.18043	-2.55011	-1.20781
H	1.01364	2.16311	-2.78112
H	2.02857	3.46729	-2.04661
H	-0.16064	4.71600	-1.33353
H	-1.86398	4.27266	0.83741
H	-1.50903	0.65321	1.79859
H	-2.53618	2.10890	2.02550
C	0.65319	-1.02169	2.50108
H	0.46325	-1.88759	1.89362
C	0.78806	-0.30717	3.49123
H	0.94952	0.47739	4.20296

IM1”

E(RB97D) = -3449.152765 E_h
Sum of electronic and thermal Enthalpies = -3448.680076 E_h
Sum of electronic and thermal Free Energies = -3448.781605 E_h
Number of imaginary frequencies 0

Ni	0.81794	0.04337	-0.89110
Cl	-0.64004	-3.20270	0.24155
Si	-0.95057	-0.98911	0.51996
Si	-3.78182	0.23991	0.56363
N	0.39807	2.92397	-0.14675
N	2.80730	-2.02740	-0.09183
N	-2.55916	-0.77370	-0.21846
N	2.49275	2.43230	-0.22491
N	2.43362	-0.67936	1.54730
C	1.24522	1.86465	-0.38385
C	1.08840	4.09141	0.16343
C	2.04060	-0.93700	0.24944
C	1.89518	0.39749	2.37358
H	2.44188	1.33778	2.20336
C	2.41665	3.78021	0.11719
C	-0.10344	0.25446	-2.54376
C	3.63066	-2.43602	0.95011
C	0.09299	-0.99946	-2.30726
C	-1.04855	2.85500	-0.32525
H	-1.32201	1.80101	-0.44590
C	3.39780	-1.58026	1.98749

C	2.66134	-2.75655	-1.34942
H	2.54700	-2.03579	-2.16795
C	3.74628	1.70054	-0.38300
H	4.10000	1.30868	0.58352
C	-4.40837	1.55380	-0.67382
H	-3.61102	2.24468	-0.99298
H	-5.22530	2.15674	-0.23965
H	-4.80340	1.06938	-1.58321
C	-5.27863	-0.82408	1.07721
H	-5.73950	-1.33746	0.21691
H	-6.06026	-0.20361	1.55058
H	-4.97313	-1.59821	1.80094
C	-3.10542	1.07848	2.13420
H	-2.87059	0.33182	2.90985
H	-3.87568	1.75909	2.54112
H	-2.19098	1.66364	1.95702
C	-3.02000	-1.44300	-1.45702
H	-2.72369	-0.85481	-2.34296
H	-2.56857	-2.44240	-1.53940
H	-4.11732	-1.57289	-1.47153
H	-1.55855	3.27870	0.55165
H	-1.34553	3.41660	-1.22544
H	4.50445	2.37430	-0.80787
H	3.56719	0.85907	-1.06152
H	3.30007	4.38643	0.28534
H	0.58074	5.02614	0.37448
H	0.83811	0.53266	2.11372
H	1.98451	0.10630	3.42983
H	3.81690	-1.53433	2.98638
H	4.28645	-3.29485	0.86367
H	1.76433	-3.38969	-1.30141
H	3.55852	-3.37307	-1.50450
H	-0.51261	0.99961	-3.21919
H	-0.07258	-2.02419	-2.62367

TS2”

E(RB97D) = -3449.144831 E_h
Sum of electronic and thermal Enthalpies = -3448.673712 E_h
Sum of electronic and thermal Free Energies = -3448.775502 E_h
Number of imaginary frequencies 1, v = -133.8 cm^{-1}

Ni	0.91409	0.06192	-0.85554
Cl	-1.16607	-3.14035	-0.03310
Si	-1.10441	-0.89695	-0.06822
Si	-3.72463	0.57529	0.66683
N	0.98686	2.89507	-0.16468
N	2.48081	-2.32676	0.02973
N	-2.79640	-0.45180	-0.41993
N	2.98723	2.08404	-0.28958
N	2.22980	-0.92841	1.65254
C	1.66531	1.73204	-0.46225
C	1.85555	3.91891	0.19724
C	1.96499	-1.08647	0.31325
C	1.73490	0.20972	2.42226

H	2.26968	1.12897	2.14324
C	3.11919	3.40765	0.12256
C	0.02089	0.33896	-2.44855
C	3.01234	-2.93579	1.16091
C	-0.47436	-0.84154	-2.09951
C	-0.46424	3.04330	-0.25954
H	-0.90986	2.04102	-0.22625
C	2.85539	-2.04980	2.18865
C	2.31498	-2.98551	-1.26343
H	2.19509	-2.20597	-2.02682
C	4.10416	1.15912	-0.45333
H	4.38985	0.71655	0.51396
C	-4.26037	2.16664	-0.25056
H	-3.38565	2.77673	-0.53514
H	-4.92328	2.79458	0.37079
H	-4.80629	1.92357	-1.17838
C	-5.30417	-0.32720	1.24338
H	-5.92990	-0.64534	0.39254
H	-5.92496	0.32152	1.88655
H	-5.04349	-1.23119	1.81902
C	-2.71766	1.06829	2.20638
H	-2.36782	0.18186	2.75935
H	-3.35686	1.66563	2.88258
H	-1.83178	1.67048	1.95242
C	-3.50729	-0.97041	-1.60889
H	-3.16937	-0.45704	-2.52733
H	-3.31819	-2.05055	-1.72516
H	-4.60091	-0.83538	-1.52630
H	-0.82535	3.64527	0.58703
H	-0.73965	3.53522	-1.20563
H	4.96083	1.69982	-0.88219
H	3.77999	0.35704	-1.12628
H	4.08627	3.85918	0.31363
H	1.50061	4.90868	0.46153
H	0.66358	0.33115	2.19753
H	1.88413	0.00366	3.49121
H	3.12963	-2.11238	3.23576
H	3.44410	-3.92970	1.13306
H	1.40958	-3.60992	-1.24611
H	3.20346	-3.59851	-1.47728
H	0.04156	0.99656	-3.31833
H	-0.72536	-1.71845	-2.70941

6

E(RB97D) = -3449.190864 E_h
Sum of electronic and thermal Enthalpies = -3448.716416 E_h
Sum of electronic and thermal Free Energies = -3448.813325 E_h
Number of imaginary frequencies 0

Ni	0.61829	0.13142	-0.66883
Cl	-2.29946	-2.05317	-1.91381
Si	-1.51064	-0.07235	-1.36802
Si	-2.73347	0.96533	1.26893
N	2.70008	2.21453	-0.18545
N	0.81340	-2.63341	0.33216
N	-2.83968	0.65794	-0.45579

N	3.60187	0.26577	-0.35833
N	0.98343	-1.23507	1.96456
C	2.37837	0.89418	-0.41288
C	4.06575	2.39115	0.01825
C	0.77775	-1.28153	0.60140
C	1.01221	0.00113	2.73462
H	1.68982	-0.12539	3.59199
C	4.63800	1.15644	-0.09476
C	0.17531	1.17401	-2.25475
C	1.01984	-3.38885	1.48114
C	-1.02504	0.89855	-2.85592
C	1.71607	3.29351	-0.11020
H	0.77983	2.92559	-0.54478
C	1.11842	-2.50630	2.51708
C	0.77385	-3.20306	-1.01245
H	0.56014	-2.39323	-1.71891
C	3.76658	-1.17647	-0.51707
H	3.62969	-1.69369	0.44515
C	-4.35314	1.77332	1.87243
H	-4.56112	2.72059	1.34654
H	-4.27914	2.00121	2.95032
H	-5.22474	1.11234	1.73115
C	-2.46955	-0.63829	2.27041
H	-3.36123	-1.28278	2.18626
H	-2.30085	-0.43829	3.34450
H	-1.61459	-1.21453	1.88587
C	-1.30646	2.18833	1.59307
H	-0.38246	1.81759	1.12113
H	-1.11290	2.35317	2.66825
H	-1.54484	3.16212	1.13173
C	-4.12779	0.76068	-1.17691
H	-3.95747	1.10438	-2.21262
H	-4.65338	-0.21040	-1.22790
H	-4.79354	1.49124	-0.69061
H	1.54010	3.58195	0.93804
H	2.08435	4.16369	-0.67376
H	4.77271	-1.38173	-0.91013
H	3.00874	-1.53308	-1.22338
H	5.67346	0.83994	-0.03354
H	4.50147	3.36631	0.20545
H	1.37835	0.80055	2.07963
H	0.00493	0.26077	3.08786
H	1.25708	-2.66197	3.58109
H	1.07482	-4.47141	1.45600
H	-0.02569	-3.95218	-1.08149
H	1.74661	-3.66625	-1.24836
H	0.91221	1.86529	-2.71074
H	-1.38077	1.27143	-3.82626

TS3”

E(RB97D) = -3526.442159 E_h
Sum of electronic and thermal Enthalpies = -3525.936815 E_h
Sum of electronic and thermal Free Energies = -3526.038558 E_h
Number of imaginary frequencies 1, v = -101.0 cm^{-1}

Ni	-0.19619	-0.51072	0.63646

Cl	2.21959	-0.36009	-2.10636
Si	1.95299	-0.22793	0.08814
Si	2.80512	2.09457	1.98996
N	-1.76211	-3.17294	0.06295
N	-0.88981	2.16563	-0.74999
N	3.04441	1.11455	0.53861
N	-1.68691	-1.85083	-1.62897
N	-2.56989	1.24181	0.23085
C	-1.35766	-1.90102	-0.29017
C	-2.30061	-3.87874	-1.00902
C	-0.42795	0.08463	2.79966
C	-1.20947	1.05960	0.01837
C	-1.38671	-0.61247	3.16263
C	-3.44010	0.35705	0.99769
H	-3.04008	-0.65964	0.92580
C	-2.25280	-3.03723	-2.08095
C	1.09148	-1.87681	1.23120
C	-1.99232	2.97218	-1.00174
C	2.38668	-1.84766	0.81080
C	-1.62887	-3.74855	1.39508
H	-1.42299	-2.93419	2.09606
C	-3.05425	2.39296	-0.37594
C	0.41353	2.47091	-1.34012
H	1.21508	2.27387	-0.62280
C	-1.47616	-0.68719	-2.49135
H	-0.49055	-0.26479	-2.27686
C	4.17534	3.41873	2.08912
H	5.17161	2.98779	2.28082
H	3.95105	4.11647	2.91500
H	4.23498	4.01008	1.15937
C	1.14827	3.03502	1.93345
H	1.22663	3.89352	1.24499
H	0.90361	3.43381	2.93380
H	0.30900	2.41329	1.59447
C	2.92938	1.02519	3.56966
H	2.33466	0.10157	3.48612
H	2.60861	1.56716	4.47707
H	3.97821	0.71604	3.72291
C	4.46760	0.85367	0.18303
H	5.05547	1.78378	0.23510
H	4.93965	0.10626	0.85033
H	4.53730	0.47800	-0.84908
H	-2.56504	-4.26016	1.66950
H	-0.79640	-4.46766	1.42599
H	-2.25404	0.07080	-2.31576
H	-1.51392	-1.02030	-3.53789
H	-2.55761	-3.17407	-3.11253
H	-2.65407	-4.89947	-0.91351
H	-3.46882	0.66197	2.05338
H	-4.45196	0.40133	0.56729
H	-4.09251	2.69428	-0.29363
H	-1.91499	3.87716	-1.59317
H	0.42225	3.53674	-1.60927
H	0.58829	1.86084	-2.23544
H	-2.22319	-1.27067	3.26900
H	0.32932	0.81270	3.02168

H	3.18146	-2.56925	1.02942
H	0.68315	-2.64192	1.91436

IM2”

E(RB97D) = -3526.447465 E_h
Sum of electronic and thermal Enthalpies = -3525.941487 E_h
Sum of electronic and thermal Free Energies = -3526.042673 E_h
Number of imaginary frequencies 0

Ni	0.91582	-0.22900	-0.86837
Cl	-1.32542	-0.55041	2.23659
Si	-1.05206	-0.91403	0.08744
Si	-4.15677	-0.09786	0.20969
N	3.22704	-1.92526	0.15911
N	0.30276	2.68201	-0.04725
N	-2.61347	-0.39048	-0.61576
N	2.61904	-0.51415	1.66361
N	2.40178	2.30997	-0.32878
C	2.32633	-0.90567	0.37475
C	4.03762	-2.15854	1.26470
C	0.59210	0.32056	-2.78266
C	1.17670	1.67305	-0.36816
C	1.80668	-0.04308	-2.78198
C	3.69391	1.67775	-0.58298
H	3.53540	0.83654	-1.26321
C	3.65624	-1.26007	2.21636
C	0.24314	-2.07978	-1.17572
C	0.95347	3.88438	0.20518
C	-0.67252	-2.64700	-0.34145
C	3.30683	-2.71004	-1.06821
H	2.93561	-2.08608	-1.88862
C	2.28458	3.64951	0.02661
C	-1.14506	2.52414	0.07852
H	-1.40443	2.16473	1.08299
C	1.99148	0.59447	2.37944
H	0.98936	0.74593	1.97631
C	-4.71076	-1.59031	1.25457
H	-4.79725	-2.49477	0.62778
H	-5.69659	-1.40731	1.71870
H	-3.98849	-1.80454	2.05774
C	-4.12704	1.46355	1.30330
H	-3.39324	1.38829	2.11913
H	-5.12321	1.61452	1.75651
H	-3.89529	2.36301	0.70779
C	-5.49055	0.20929	-1.12412
H	-5.25704	1.08469	-1.75398
H	-6.45785	0.40509	-0.62919
H	-5.62627	-0.65692	-1.79233
C	-2.71430	-0.70576	-2.06388
H	-3.25372	-1.65308	-2.25550
H	-3.23726	0.09907	-2.60763
H	-1.71298	-0.81272	-2.50356
H	4.35506	-2.99579	-1.24243
H	2.68215	-3.61195	-0.99052
H	2.58530	1.51531	2.26293

H	1.91792	0.33195	3.44383
H	4.01160	-1.09021	3.22656
H	4.79074	-2.93858	1.27495
H	4.36618	2.42085	-1.03603
H	4.13410	1.30314	0.35428
H	3.14516	4.30569	0.09391
H	0.41238	4.78618	0.46805
H	-1.49872	1.78867	-0.65011
H	-1.62027	3.49941	-0.09988
H	2.73554	-0.15589	-3.32801
H	-0.28488	0.72122	-3.27521
H	-1.06022	-3.67239	-0.37797
H	0.71078	-2.57166	-2.04706

TS4”

E(RB97D) = -3526.424408 E_h
Sum of electronic and thermal Enthalpies = -3525.919584 E_h
Sum of electronic and thermal Free Energies = -3526.018804 E_h
Number of imaginary frequencies 1, v = -212.6 cm^{-1}

Ni	0.47283	-0.59914	-0.56728
Cl	-2.22518	-0.32972	1.82801
Si	-1.94094	-0.51192	-0.36544
Si	-4.53434	1.42397	-0.12382
N	2.54885	-2.55743	0.01678
N	0.93249	2.29215	0.23305
N	-3.34644	0.40348	-0.95458
N	2.07414	-1.37509	1.76697
N	2.75314	1.31471	-0.37230
C	1.70403	-1.56139	0.45847
C	3.42359	-2.95955	1.02059
C	-0.62168	0.30084	-2.06547
C	1.41187	1.07505	-0.17993
C	0.39939	-0.27687	-2.65696
C	3.68620	0.32347	-0.90213
H	3.14343	-0.28471	-1.63716
C	3.12676	-2.21199	2.12400
C	-0.47742	-2.33350	-0.70788
C	1.93730	3.25215	0.29533
C	-1.83323	-2.30276	-0.71295
C	2.60344	-3.04286	-1.36133
H	1.97184	-2.37752	-1.97168
C	3.09240	2.63292	-0.08535
C	-0.47389	2.56182	0.52534
H	-0.82227	1.90941	1.33222
C	1.46932	-0.37635	2.64521
H	0.41479	-0.27052	2.36842
C	-5.74114	0.39725	0.93159
H	-6.25142	-0.36167	0.31318
H	-6.51907	1.03371	1.39035
H	-5.20538	-0.12960	1.73655
C	-3.73715	2.77963	0.95206
H	-3.10391	2.36747	1.75025
H	-4.53454	3.37822	1.42756
H	-3.12969	3.46812	0.34080

C	-5.55026	2.33834	-1.45918
H	-4.90244	2.92691	-2.13143
H	-6.25066	3.03978	-0.97288
H	-6.14645	1.65304	-2.08350
C	-3.75679	-0.02629	-2.32056
H	-4.69354	-0.61507	-2.30028
H	-3.90946	0.84113	-2.98466
H	-2.98200	-0.65880	-2.78218
H	3.64714	-3.01586	-1.71234
H	2.22497	-4.07428	-1.42135
H	1.97972	0.59292	2.53253
H	1.55419	-0.72272	3.68517
H	3.54433	-2.21433	3.12438
H	4.15628	-3.74302	0.86370
H	4.52248	0.84970	-1.38393
H	4.07014	-0.32538	-0.10077
H	4.10597	3.00395	-0.18627
H	1.73703	4.27379	0.59736
H	-1.08770	2.37599	-0.36535
H	-0.57327	3.61310	0.82598
H	0.84861	0.05246	-3.60255
H	-1.22032	1.20391	-2.23128
H	-2.50059	-3.16019	-0.86571
H	0.10493	-3.26181	-0.80324

IM3”

E(RB97D) = -3526.526266 E_h
Sum of electronic and thermal Enthalpies = -3526.018505 E_h
Sum of electronic and thermal Free Energies = -3526.12184 E_h
Number of imaginary frequencies 0

Ni	1.26032	-0.07141	-0.66024
Cl	-1.60655	-0.23232	2.25891
Si	-2.00459	-0.54775	0.11948
Si	-4.91660	0.31520	-0.47418
N	3.50334	-2.04875	-0.64632
N	1.96307	2.49519	0.74045
N	-3.71649	-0.89058	0.02292
N	2.96809	-1.41660	1.34223
N	3.41342	1.97849	-0.76544
C	2.65019	-1.20820	0.02619
C	4.33033	-2.75363	0.22741
C	-1.48824	0.95935	-0.83716
C	2.23065	1.54521	-0.21379
C	-0.20318	1.01434	-1.28419
C	4.11680	1.24769	-1.81252
H	3.37630	0.65074	-2.35851
C	3.98849	-2.35196	1.48706
C	0.27520	-1.70541	-0.95890
C	2.95378	3.47238	0.79088
C	-0.95952	-1.96985	-0.45710
C	3.51131	-2.19386	-2.09798
H	2.62787	-1.67168	-2.48677

C	3.87332	3.14676	-0.16418
C	0.79509	2.46226	1.62138
H	1.06856	2.06120	2.60918
C	2.28736	-0.72750	2.43849
H	1.23248	-0.59074	2.16886
C	-6.65784	-0.38502	-0.14214
H	-6.85555	-1.29886	-0.72723
H	-7.41850	0.36244	-0.42714
H	-6.80887	-0.62678	0.92310
C	-4.71302	1.92243	0.52894
H	-4.86699	1.72411	1.60325
H	-5.44123	2.69130	0.21463
H	-3.70201	2.34617	0.41833
C	-4.74640	0.67514	-2.33499
H	-3.70535	0.91983	-2.59876
H	-5.38931	1.51689	-2.64822
H	-5.03666	-0.21151	-2.92444
C	-4.16656	-2.11251	0.72749
H	-4.38091	-1.92326	1.79593
H	-5.07583	-2.52273	0.26034
H	-3.39037	-2.89451	0.67974
H	4.42557	-1.75278	-2.52730
H	3.46022	-3.25929	-2.36747
H	2.75284	0.25357	2.62112
H	2.35824	-1.34384	3.34550
H	4.36203	-2.65276	2.45948
H	5.06278	-3.47344	-0.12047
H	4.60380	1.96034	-2.49444
H	4.87343	0.57529	-1.37754
H	4.78498	3.64137	-0.48052
H	2.90232	4.30839	1.47942
H	0.03703	1.82091	1.16183
H	0.40194	3.48287	1.73617
H	0.05382	1.86206	-1.94640
H	-2.20713	1.73947	-1.12331
H	-1.38323	-2.98408	-0.52169
H	0.80992	-2.55583	-1.42167

TS5”

E(RB97D) = -3526.491612 E_h
Sum of electronic and thermal Enthalpies = -3525.986943 E_h
Sum of electronic and thermal Free Energies = -3526.09313 E_h
Number of imaginary frequencies 1, v = -439.7 cm^{-1}

Ni	-0.90877	0.42336	-0.65321
Cl	2.30905	-0.01202	1.96609
Si	2.54422	0.03794	-0.18715
Si	5.00271	-1.61821	-1.02263
N	-2.11816	3.19574	-0.60627
N	-1.87391	-2.06464	0.75499
N	4.25040	-0.09340	-0.51126
N	-1.61918	2.47377	1.35976
N	-3.42061	-1.14902	-0.43088
C	-1.64612	2.07175	0.04257
C	-2.36083	4.24939	0.27293

C	1.52782	-1.22330	-1.09365
C	-2.09424	-0.98683	-0.07738
C	0.41006	-0.60198	-1.62493
C	-4.10757	-0.24720	-1.34583
H	-3.67607	0.75372	-1.21141
C	-2.03657	3.79121	1.51818
C	0.61556	1.26032	-1.54868
C	-3.01331	-2.84772	0.91762
C	1.79536	1.58140	-0.91966
C	-2.32294	3.25907	-2.04674
H	-2.03398	2.28470	-2.46173
C	-3.99170	-2.27412	0.15711
C	-0.61553	-2.27969	1.46617
H	-0.64053	-1.79943	2.45733
C	-1.09978	1.62065	2.42624
H	-0.00724	1.71462	2.50357
C	6.88762	-1.46332	-0.79789
H	7.32462	-0.69524	-1.45777
H	7.37333	-2.42394	-1.04226
H	7.15483	-1.20707	0.24085
C	4.36799	-3.05264	0.05649
H	4.66776	-2.90644	1.10787
H	4.77732	-4.02014	-0.28448
H	3.26876	-3.12402	0.03702
C	4.61396	-1.96024	-2.85290
H	3.52605	-1.91686	-3.02326
H	4.97759	-2.95246	-3.17398
H	5.08241	-1.20025	-3.50163
C	5.09264	1.04804	-0.09088
H	5.41667	0.95820	0.96253
H	5.99230	1.12552	-0.72161
H	4.53748	1.99609	-0.19139
H	-3.38109	3.46405	-2.27965
H	-1.69563	4.04760	-2.49149
H	-1.34139	0.58129	2.16873
H	-1.57567	1.90147	3.37791
H	-2.06879	4.27680	2.48759
H	-2.74669	5.20759	-0.05785
H	-3.96933	-0.56409	-2.39250
H	-5.18184	-0.23469	-1.10701
H	-5.02128	-2.56399	-0.02241
H	-3.02414	-3.72741	1.55190
H	0.19490	-1.84444	0.87096
H	-0.45163	-3.36056	1.58983
H	-0.07360	-1.05918	-2.50727
H	1.84974	-2.20749	-1.44058
H	2.32016	2.51857	-1.12856
H	0.25069	1.90630	-2.36700

IM4”

E(RB97D) = -3526.570116 E_h
Sum of electronic and thermal Enthalpies = -3526.061362 E_h
Sum of electronic and thermal Free Energies = -3526.166306 E_h
Number of imaginary frequencies 0

Ni	-0.75482	0.00509	-0.62643
Cl	2.38389	1.41048	1.13400
Si	2.38973	-0.36789	-0.08965
Si	5.19034	-1.62302	0.04536
N	-0.44412	2.84108	-1.48554
N	-2.77307	-1.18469	1.21758
N	4.02412	-0.51112	-0.69229
N	-0.89206	2.67951	0.61547
N	-3.67918	-0.41230	-0.58058
C	-0.74061	1.90505	-0.51471
C	-0.38171	4.13169	-0.96825
C	1.75529	-1.85786	0.83207
C	-2.44580	-0.56242	0.02526
C	0.70970	-2.33854	0.08714
C	-3.83750	0.18533	-1.90013
H	-2.99815	0.87810	-2.05228
C	-0.65754	4.02673	0.36426
C	0.30507	-1.60935	-1.13582
C	-4.14312	-1.40939	1.33510
C	1.04395	-0.38296	-1.38533
C	-0.16200	2.50269	-2.87409
H	-0.38184	1.43641	-3.00375
C	-4.71550	-0.92674	0.19393
C	-1.79293	-1.52880	2.24154
H	-2.03220	-1.01339	3.18628
C	-1.12824	2.11401	1.93898
H	-0.17894	1.77402	2.37821
C	6.92616	-1.21149	-0.62689
H	6.99966	-1.36590	-1.71666
H	7.67541	-1.86676	-0.14963
H	7.21099	-0.16738	-0.41445
C	5.20212	-1.41442	1.93841
H	5.45014	-0.37555	2.21313
H	5.94361	-2.08291	2.41109
H	4.21722	-1.64271	2.37650
C	4.74965	-3.41990	-0.39254
H	3.70308	-3.62678	-0.11585
H	5.39962	-4.14277	0.13197
H	4.84975	-3.59505	-1.47746
C	4.45991	0.53745	-1.63725
H	4.77003	1.46599	-1.12135
H	5.30532	0.18684	-2.24942
H	3.63816	0.79615	-2.32925
H	-0.79126	3.10634	-3.54772
H	0.89967	2.68401	-3.10617
H	-1.80551	1.25890	1.82813
H	-1.58801	2.88128	2.57966
H	-0.71184	4.77825	1.14402
H	-0.16261	4.99482	-1.58704
H	-3.81868	-0.58529	-2.68811
H	-4.79283	0.73036	-1.94414
H	-5.75005	-0.90228	-0.13089
H	-4.58040	-1.88015	2.20886
H	-0.80452	-1.21759	1.88274
H	-1.78696	-2.61659	2.41475
H	0.13831	-3.22945	0.37946

H	2.05905	-2.31305	1.77538
H	1.19995	-0.04517	-2.41557
H	-0.16304	-2.18863	-1.94139

1 T. J. Hadlington, T. Szilvasi, M. Driess, *Angew. Chem. Int. Ed.* **2017**, *56*, 7470-7474.

2 G. M. Sheldrick, Acta Cryst., 2015, A71, 3-8

3 G. M. Sheldrick, Acta Cryst., 2015, C71, 3-8

4 (a) Frisch, M. J.; Pople, J. A.; Binkley, V. J. Chem. Phys., 1984, 80, 3265-3269; (b) A. D. Becke, J. Chem. Phys., 1997, 107, 8554-8560; (c) S. Grimme J. Comput. Chem., 2006, 27, 1787-1799.

5 Gaussian 09, Revision D.01, Frisch, M. J.; Trucks, G. W.; Schlegel, H. B.; Scuseria, G. E.; Robb, M. A.; Cheeseman, J. R.; Scalmani, G.; Barone, V.; Mennucci, B.; Petersson, G. A.; Nakatsuji, H.; Caricato, M.; Li, X.; Hratchian, H. P.; Izmaylov, A. F.; Bloino, J.; Zheng, G.; Sonnenberg, J. L.; Hada, M.; Ehara, M.; Toyota, K.; Fukuda, R.; Hasegawa, J.; Ishida, M.; Nakajima, T.; Honda, Y.; Kitao, O.; Nakai, H.; Vreven, T.; Montgomery, J. A.; Jr., J. E. P.; Ogliaro, F.; Bearpark, M.; Heyd, J. J.; Brothers, E.; Kudin, K. N.; Staroverov, V. N.; Kobayashi, R.; Normand, J.; Raghavachari, K.; Rendell, A.; Burant, J. C.; Iyengar, S. S.; Tomasi, J.; Cossi, M.; Rega, N.; Millam, J. M.; Klene, M.; Knox, J. E.; Cross, J. B.; Bakken, V.; Adamo, C.; Jaramillo, J.; Gomperts, R.; Stratmann, R. E.; Yazyev, O.; Austin, A. J.; Cammi, R.; Pomelli, C.; Ochterski, J. W.; Martin, R. L.; Morokuma, K.; Zakrzewski, V. G.; Voth, G. A.; Salvador, P.; Dannenberg, J. J.; Dapprich, S.; Daniels, A. D.; Farkas, Ö.; Foresman, J. B.; Ortiz, J. V.; Cioslowski, J.; Fox, D. J.; Gaussian, Inc., Wallingford CT, 2009.
